# Systematic functional analysis of kinases in the fungal pathogen *Cryptococcus neoformans*

**DOI:** 10.1038/ncomms12766

**Published:** 2016-09-28

**Authors:** Kyung-Tae Lee, Yee-Seul So, Dong-Hoon Yang, Kwang-Woo Jung, Jaeyoung Choi, Dong-Gi Lee, Hyojeong Kwon, Juyeong Jang, Li Li Wang, Soohyun Cha, Gena Lee Meyers, Eunji Jeong, Jae-Hyung Jin, Yeonseon Lee, Joohyeon Hong, Soohyun Bang, Je-Hyun Ji, Goun Park, Hyo-Jeong Byun, Sung Woo Park, Young-Min Park, Gloria Adedoyin, Taeyup Kim, Anna F. Averette, Jong-Soon Choi, Joseph Heitman, Eunji Cheong, Yong-Hwan Lee, Yong-Sun Bahn

**Affiliations:** 1 Department of Biotechnology, College of Life Science and Biotechnology, Yonsei University, Seoul 03722, Korea; 2 Department of Agricultural Biotechnology, Seoul National University, Seoul 08826, Korea; 3 Biological Disaster Analysis Group, Korea Basic Science Institute, Daejeon 34133, Korea; 4 Departments of Molecular Genetics and Microbiology, Medicine, and Pharmacology and Cancer Biology, Duke University Medical Center, Durham, North Carolina 27710, USA

## Abstract

*Cryptococcus neoformans* is the leading cause of death by fungal meningoencephalitis; however, treatment options remain limited. Here we report the construction of 264 signature-tagged gene-deletion strains for 129 putative kinases, and examine their phenotypic traits under 30 distinct *in vitro* growth conditions and in two different hosts (insect larvae and mice). Clustering analysis of *in vitro* phenotypic traits indicates that several of these kinases have roles in known signalling pathways, and identifies hitherto uncharacterized signalling cascades. Virulence assays in the insect and mouse models provide evidence of pathogenicity-related roles for 63 kinases involved in the following biological categories: growth and cell cycle, nutrient metabolism, stress response and adaptation, cell signalling, cell polarity and morphology, vacuole trafficking, transfer RNA (tRNA) modification and other functions. Our study provides insights into the pathobiological signalling circuitry of *C. neoformans* and identifies potential anticryptococcal or antifungal drug targets.

## Abstract (web summary)

*Cryptococcus neoformans* is the leading cause of death by fungal meningoencephalitis. Here, the authors study the roles played by 129 putative kinases in the growth and virulence of *C. neoformans*, identifying potential targets for development of anticryptococcal drugs.

## Graphical abstract

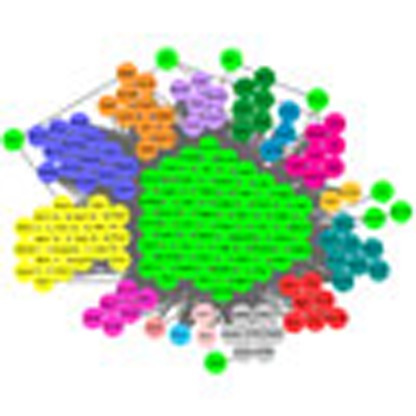

C*ryptococcus neoformans* is the leading cause of fungal meningoencephalitis and is responsible for over a million infections and 600,000 deaths annually on a global scale^1^. This pathogenic yeast is ubiquitously distributed in diverse natural environments. It is considered to be a cosmopolitan fungal pathogen that utilizes a wide variety of living hosts ranging from lower eukaryotes to animals, suggesting broad distribution in the environment under conditions that lead to the production of spores and desiccated yeast cells that are both documented to be infectious propagules^2^. Nevertheless, treatment of systemic cryptococcosis remains challenging because only limited therapeutic options are available^3^. In addition to its clinical importance, *C. neoformans* is regarded as an ideal fungal model system for basidiomycetes, which have been diverging for at least 500 million years from their last common ancestor shared with the ascomycetes, owing to the availability of completely sequenced and well-annotated genome databases, a classical genetic dissection method through sexual differentiation, efficient methods of reverse and forward genetics, and a variety of heterologous host model systems^4^.

Extensive research efforts have been made over several decades to understand the mechanisms underlying the pathogenicity of *C. neoformans*. Besides efforts to analyse the functions of individual genes and proteins, recent large-scale functional genetic analyses have provided comprehensive insights into the overall biological circuitry of *C. neoformans*^5^,^6^,^7^. However, a complete picture of the pathobiological networks of *C. neoformans* remains elusive, mainly because functions of kinases, which have a central role in signalling and metabolic pathways, have not been fully characterized on a genome-wide scale. In general, kinases have pivotal roles in growth, cell cycle control, differentiation, development, the stress response and many other cellular functions, affecting ∼30% of cellular proteins by phosphorylation^8^. Furthermore, kinases are considered to be the second largest protein class for drug targets in clinical trials as their inhibition is readily possible by small molecules or antibodies^9^. The systematic functional profiling of kinases in human fungal pathogens is in high demand to identify virulence-related kinases that could be further developed as antifungal drug targets.

In this study, we performed systematic functional profiling of the kinome networks in *C. neoformans* by constructing a high-quality library of 264 signature-tagged gene-deletion strains through homologous recombination methods for 129 putative kinases, and examining their phenotypic traits under 30 distinct *in vitro* conditions, including growth, differentiation, stress responses, antifungal resistance and virulence-factor production. Furthermore, we investigated their virulence and infectivity potential in insect and murine host models. This phenotypic data set is freely accessible to the public through the *Cryptococcus* kinome database (http://kinase.cryptococcus.org). The data presented in this study provides insights into the pathobiological signalling circuitry of *C. neoformans* and may help development of novel therapies for cryptococcosis targeting unique virulence-related kinases.

## Results

### Identification of kinases in *C. neoformans*

**Figure 1:**
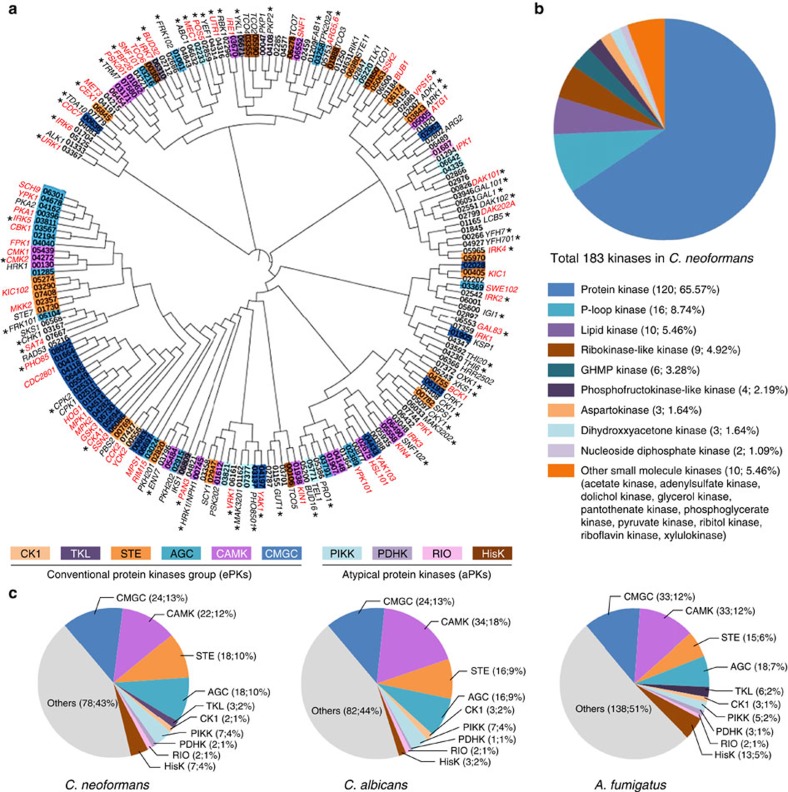
Phylogenetic correlation among kinases in *Cryptococcus neoformans* and kinase distribution in human fungal pathogens. (**a**) Protein sequence-based alignment was performed using ClustalX2 windows interface program run by University College Dublin. Using this alignment data, the phylogenetic tree was illustrated by a web-based drawing application named Interactive Tree Of Life (http://itol.embl.de). Among the 183 kinases found in *C. neoformans*, we constructed signature-tagged gene deletion strains for 129 kinases genes. Asterisks indicate the kinases that were first functionally characterized by this study and named based on the published nomenclature rules for *C. neoformans* genes^70^. The different colour codes represent the different classes of protein kinases predicted by Kinomer 1.0 (http://www.compbio.dundee.ac.uk/kinomer)^12^. Red marked genes indicate the 63 pathogenicity-related kinases discovered in this study. (**b**) Pie-chart for the classification of 183 kinases in *C. neoformans*. The description of the Broad institute database was grouped by using previously reported classification methods^10^. (**c**) Pie-chart for the kinase classes predicted by Kinomer 1.0 to reveal the relative portion of protein kinase classes in human fungal pathogens, *C. neoformans*, *Candida albicans* and *Aspergillus fumigatus*.

To select the putative kinase genes in the genome of *C. neoformans* var. *grubii* (H99 strain), we surveyed a curated annotation of kinases in the H99 genome database provided by the Broad Institute (http://www.broadinstitute.org/annotation/genome/cryptococcus_neoformans) and the JEC21 genome database within the database of the National Center for Biotechnology Information. For each gene that had a kinase-related annotation, we performed protein domain analyses using Pfam (http://pfam.xfam.org/) to confirm the presence of kinase domains and to exclude the genes with annotations such as phosphatases or kinase regulators. Through this analysis, we retrieved 183 putative kinase genes in *C. neoformans* (Supplementary Data 1). The phylogenetic relationship is shown in Fig. 1a.

The 183 putative kinases were classified into 19 different families based on their sequence and structure similarities (Fig. 1b), as suggested previously^10^. The protein kinase family is the most dominant one (120) followed by P-loop kinase (16), lipid kinase (10), ribokinase-like kinase (9), GHMP kinase (6) and other small molecule kinases. Eukaryotic protein kinase superfamilies are further classified into eight conventional protein kinase groups (ePKs) and five atypical groups (aPKs)^11^. ePKs include the protein kinase A, G, and C group (AGC), the calcium and calmodulin-regulated kinase group (CAMK), the casein kinase 1 group (CK1), a group of cyclin-dependent kinases (CDKs), mitogen-activated protein kinases (MAPK), glycogen synthase kinases and CDK-like kinases (CMGC), the receptor guanylate cyclase group (RGC), a group of sterile kinase (STE), the tyrosine kinase group (TK) and the tyrosine kinase-like group (TKL). The aPKs include the phosphatidylinositol 3-kinase-related kinase group (PIKK), the ribosome biogenesis kinase group (RIO), the pyruvate dehydrogenase kinase group (PDHK), and histidine kinase (HisK). To classify the *C. neoformans* protein kinases based on these criteria, we queried their amino acid sequences in the Kinomer v.1.0 database (http://www.compbio.dundee.ac.uk/kinomer/), which systematically classifies eukaryotic protein kinases based on a hidden Markov model^12^. Although seven hybrid HisKs (Tco1–7) were not classified by the Kinomer database, we classified them here as HisK in aPKs. The *C. neoformans* protein kinases consist of 87 ePKs (18 AGC, 22 CAMK, 2 CK1, 24 CMGC, 18 STE, 3 TKL) and 18 aPKs (2 PDHK, 7 PIKK, 2 RIO, 7 HisK) (Fig. 1c). Based on our kinase domain predictions, two other major human fungal pathogens, *Candida albicans* and *Aspergillus fumigatus*, appeared to contain 188 and 269 kinases, respectively (Supplementary Data 2). Among pathogenic fungal protein kinases, CMGC, CAMK, STE and AGC kinases appear to be the most common clades (Fig. 1c).

### Construction of the *C. neoformans* kinase mutant library

To gain insights into the biological functions of *Cryptococcus* kinome networks, we aimed to construct gene-deletion mutants for each kinase and to functionally characterize them. Among the kinases analysed here, mutants for 22 kinases (*TCO1, TCO2, TCO3, TCO4, TCO5, TCO7, SSK2, PBS2, HOG1, BCK1, MKK1/2, MPK1, STE11, STE7, CPK1, PKA1, PKA2, HRK1, PKP1, IRE1, SCH9,* and *YPK1*) constructed previously were functionally characterized in part by our prior reports^13^,^14^,^15^,^16^,^17^,^18^,^19^,^20^,^21^,^22^. For the remaining 161 kinases, we attempted to construct gene-deletion mutants by using large-scale homologous recombination and by analysing their *in vitro* and *in vivo* phenotypic traits. We successfully generated 264 gene deletion mutants representing 129 kinases (including those that were previously reported) (Supplementary Data 3).

For the remaining 54 kinases, we were not able to generate mutants even after repeated attempts at gene disruption (more than four attempts, Supplementary Data 1). In many cases, we either could not isolate a viable transformant, observed the retention of a wild-type allele along with the disrupted allele (potentially aneuploidy) or incorrect genotypes were obtained. Among these, 36 (67%) are orthologous to kinases that are essential for the growth of *Saccharomyces cerevisiae* (26 genes), *Schizosaccharomyces pombe* (32 genes) or *Neurospora crassa* (7 genes, Supplementary Data 1). Notably, two genes (*MPS1* and *PIK1*) that are known to be essential in *S. cerevisiae* were successfully deleted in *C. neoformans*, suggesting the presence of functional divergence in some kinases between ascomycete and basidiomycete fungi or the presence of functionally redundant kinases in *C. neoformans*.

### Systematic phenotypic profiling of *C. neoformans* kinome

With this high-quality library of kinase mutants, we performed *in vitro* phenotypic analyses under 30 distinct growth conditions covering five major phenotypic classes: growth, differentiation, stress responses and adaptations, antifungal drug resistance and production of virulence factors (Supplementary Data 4).

**Figure 2:**
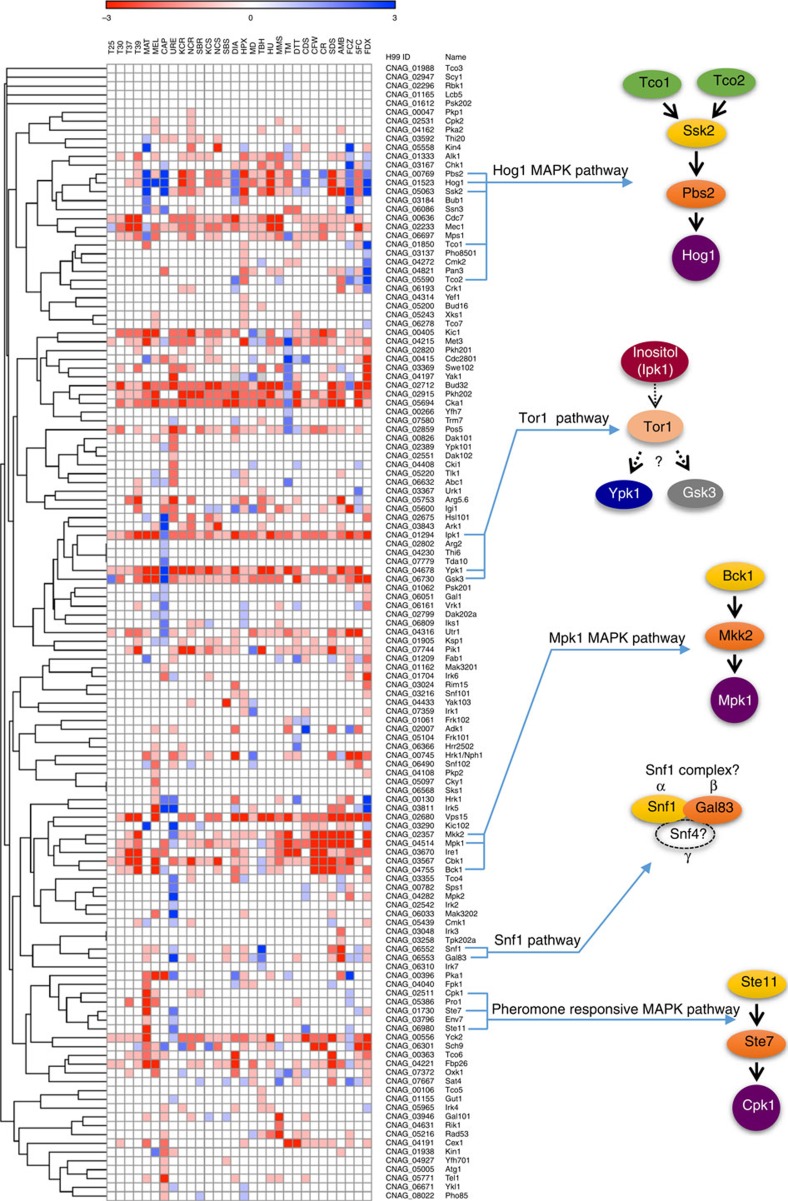
Phenotypic clustering of kinases in *Cryptococcus neoformans*. The phenotypes were scored by seven grades (−3: strongly sensitive/reduced, −2: moderately sensitive/reduced, −1: weakly sensitive/reduced, 0: wild-type like, +1: weakly resistant/increased, +2: moderately resistant/increased, +3: strongly resistant/increased). The excel file containing the phenotype scores of each kinase mutant was loaded by Gene-E software (http://www.broadinstitute.org/cancer/software/GENE-E/) and then the kinase phenome clustering was drawn using one minus Pearson correlation. T25, 25 °C; T30, 30 °C; T37, 37 °C; T39, 39 °C; CAP, capsule production; MEL, melanin production; URE, urease production; MAT, mating; HPX, hydrogen peroxide; TBH, *tert*-butyl hydroperoxide; MD, menadione; DIA, diamide; MMS, methyl methanesulphonate; HU, hydroxyurea; 5FC, 5-flucytosine; AMB, amphotericin B; FCZ, fluconazole; FDX, fludioxonil; TM, tunicamycin; DTT, dithiothreitol; CDS, cadmium sulphate; SDS, sodium dodecyl sulphate; CR, Congo red; CFW, calcofluor white; KCR, YPD+KCl; NCR, YPD+NaCl; SBR, YPD+sorbitol; KCS, YP+KCl; NCS, YP+NaCl; SBS, YP+sorbitol.

To gain insights into the functional and regulatory connectivity among kinases, we attempted to group kinases by phenotypic clustering through Pearson correlation analysis (Fig. 2). The rationale behind this was that a group of kinases in a given signalling pathway tended to cluster together in terms of shared phenotypic traits. For example, mutants in three-tier MAPK cascades should cluster together because they exhibit almost identical phenotypic traits. In fact, we found that the three-tier kinase mutants in the cell wall integrity MAPK, the high osmolarity glycerol response (HOG) MAPK, and the pheromone-responsive MAPK pathways were clustered together based on their shared functions (Fig. 2). Therefore, groups of kinases clustered together by this analysis are highly likely to function in the same or related signalling cascades.

We identified several hitherto uncharacterized kinases that are functionally correlated with these known signalling pathways. First, we identified CNAG_06553, encoding a protein orthologous to yeast Gal83 that is one of three possible β-subunits of the Snf1 kinase complex controlling the transcriptional changes under glucose derepression in *S. cerevisiae*^23^,^24^. In *C. neoformans*, Snf1 functions have been previously characterized^25^. Here we provide several lines of experimental evidence showing that Gal83 is likely to function in association with Snf1 in *C. neoformans*. First, the *in vitro* phenotypic traits of the *gal83*Δ mutant were almost equivalent to those of the *snf1*Δ mutant (Fig. 2; Supplementary Fig. 1). Both *snf1*Δ and *gal83*Δ mutants exhibited increased susceptibility to fludioxonil and increased resistance to organic peroxide. Second, growth defects in the *snf1*Δ mutant in media containing alternative carbon sources (for example, potassium acetate, sodium acetate and ethanol) were also observed in *gal83*Δ mutants (Supplementary Fig. 1). Therefore, Gal83 is likely to be one of the possible β-subunits of the Snf1 kinase complex in *C. neoformans*.

We also identified several kinases that potentially work upstream or downstream of the target of rapamycin (TOR) kinase complex. Although we were not able to disrupt Tor1 kinase, which has been suggested to be essential in *C. neoformans*^26^, we found three kinases (Ipk1, Ypk1 and Gsk3) that are potentially related to Tor1-dependent signalling cascades clustered together. Recently, Lev *et al.*^27^ proposed that Ipk1 could be involved in the production of inositol hexaphosphate (IP_6_) based on its limited sequence homology to *S. cerevisiae* Ipk1. In mammals, inositol polyphosphate multikinase produces IP_6_, a precursor of 5-IP_7_ that inhibits Akt activity and thereby decreases mTORC1-mediated protein translation and increases GSK3β-mediated glucose homeostasis and adipogenesis^28^. In *S. cerevisiae*, Ypk1 is the direct target of TORC2 by promoting autophagy during amino acid starvation^29^. In *C. neoformans*, Ypk1, which is a potential downstream target of Tor1, is involved in sphingolipid synthesis and deletion of *YPK1* resulted in a significant reduction in virulence^30^. Reflecting the essential role of Tor1, all of the mutants—*ipk1*Δ, *ypk1*Δ, and *gsk3*Δ—exhibited growth defects, particularly at high temperature (Fig. 2).

### Unravelling the pathogenic kinome networks in *C. neoformans*

**Figure 3:**
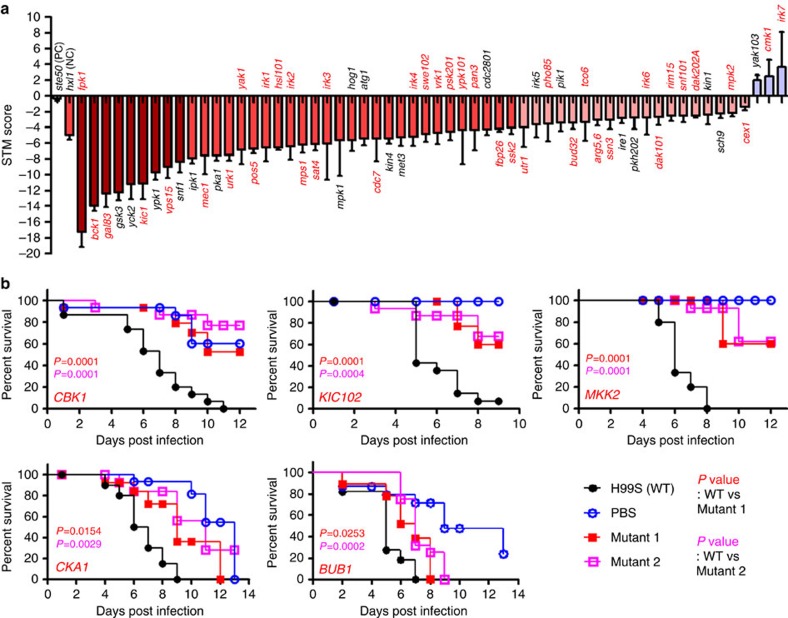
Pathogenicity-related kinases in *Cryptococcus neoformans*. (**a**) Results of the signature-tagged mutagenesis (STM)-based murine infectivity test. We used *ste50*Δ and *hxl1*Δ strains for the virulent (positive control, PC) and avirulent (negative control, NC) controls. STM scores were calculated by the quantitative PCR method, arranged numerically and coloured in gradient scales. Red marked letters show the novel infectivity-related kinases revealed by this study. Gene names for the 30 kinases that were co-identified by both insect killing and STM assays were depicted below the STM zero line. The *P*-value between control and mutant strains was determined by one-way analysis of variance employing Bonferroni correlation with three mice per each STM set. In addition, the STM score of the second independent strain was measured in another independent set with three mice. The *y* axis indicates the average value of the two independent STM scores for each kinase. This graph only shows kinases whose deletion reduces or increases STM score with statistically significant difference (*P*<0.05). The entire STM scoring was available in Supplementary Fig. 3. Error bars indicate s.e.m. (**b**) Five kinases were shown to be involved in virulence only by the wax moth killing assay. *P* values shown in the graph were calculated using the Log-rank test to measure statistical differences between the WT strain (H99S) and each kinase mutant strain.

We performed two large-scale *in vivo* animal studies: a wax moth-killing virulence assay and a signature-tagged mutagenesis (STM)-based murine infectivity assay. We found 35 virulence-related kinases in insect killing assays (Supplementary Fig. 2) and 58 infectivity-related kinases in STM-based murine infectivity assays (Supplementary Fig. 3). Among these kinases, 30 (denoted in the negative side of the STM graphs) were co-identified by both assays (Fig. 3a), whereas 5 and 28 kinases were specifically identified by the insect killing and STM-based murine assays, respectively (Fig. 3a,b). In total, we discovered 63 kinase mutants apparently involved in the pathogenicity of *C. neoformans*.

Supporting the quality of our data, 19 known virulence-related kinases were rediscovered by our study (kinases listed in black in Fig. 3a), including Mpk1 (refs 31, 32), Hog1 (ref. 15), Pka1 (ref. 33), Ire1 (ref. 22), Ypk1 (refs 17, 30) and Snf1 (ref. 25). Chang *et al.*^34^ demonstrated that Gsk3 is required for the virulence of serotype D *C. neoformans* (B3501A) in a murine model system. We found that Gsk3 is also required for the virulence of serotype A *C. neoformans* (H99S). Although not previously reported, deletion mutants of kinases functionally connected to these known virulence-related kinases were also found to be attenuated in virulence or infectivity. These include *bck1*Δ and *mkk1/2*Δ mutants (related to Mpk1) and the *gal83*Δ mutant (related to Snf1) (Fig. 2). Notably, 44 kinases have been for the first time identified to be involved in the pathogenicity of *C. neoformans*.

**Figure 4:**
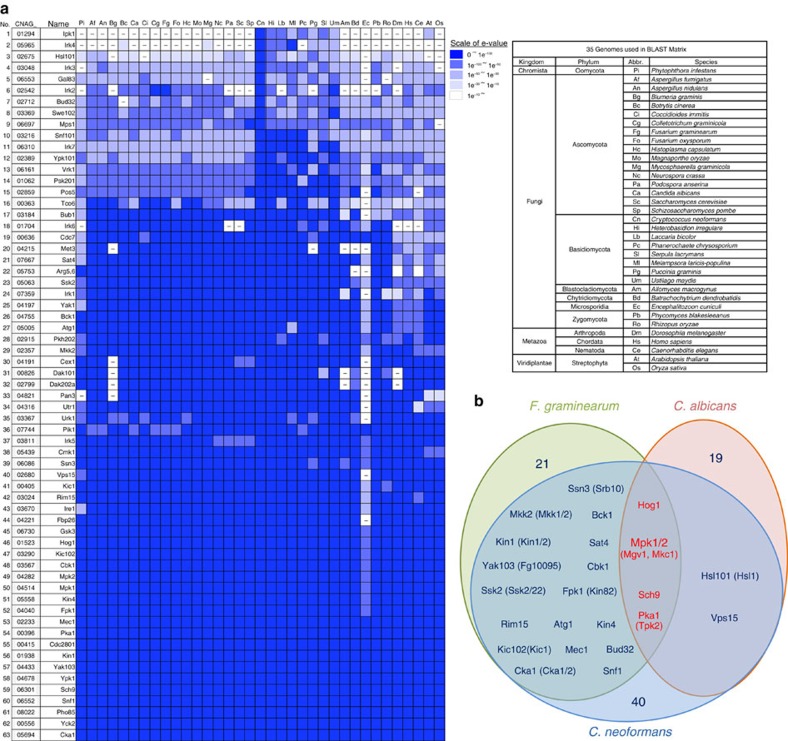
Phylogenetic relationships between pathogenicity-related kinases in *Cryptococcus neoformans* and other eukaryotic kinases. (**a**) BLAST matrix for the 63 pathogenicity-related kinases using the Comparative Fungal Genomics Platform (CFGP, http://cfgp.riceblast.snu.ac.kr) database. Using the pathogenicity-related 63 kinase protein sequence query, orthologue proteins were retrieved and matched from the genome database from the 35 eukaryotic species (Supplementary Data 7). (**b**) Correlation of the pathogenicity-related kinases in fungal pathogens. To determine the orthologue proteins among the indicated fungal pathogens, each protein sequence was analysed by BLAST and reverse-BLAST using genome databases (CGD; *Candida* genome database for *C. albicans,* Broad institute database for *Fusarium graminearum* and *C. neoformans*).

For the 63 pathogenicity-related kinases in *C. neoformans*, we analysed phylogenetic relationships among orthologs, if any, in fungal species and other eukaryotic kingdoms (Fig. 4a), and compared our large-scale virulence data of *C. neoformans* with those of other fungal pathogens. A large-scale kinome analysis was performed for the pathogenic fungus *Fusarium graminearum*, which causes scab in wheat plants, and 42 virulence-related protein kinases were identified^35^. Twenty-one were involved in the pathogenicity of both types of fungi (Fig. 4b). In another human fungal pathogen *C. albicans*, genome-wide pathogenic kinome analysis has not been performed and yet 25 kinases are known to be involved in the virulence of *C. albicans*, based on information from the *Candida* genome database (http://www.candidagenome.org/) (Supplementary Data 2). Six were involved in the pathogenicity of both *C. neoformans* and *C. albicans* (Fig. 4b). Notably, four kinases (Sch9, Pka1, Hog1 and Mpk1) appear to be core-virulence kinases as they are involved in the virulence of all three fungal pathogens.

*IPK1* (CNAG_01294), encoding a protein similar to inositol 1,3,4,5,6-pentakisphosphate 2-kinase from plants, is either not present or distantly related to those in ascomycete fungi and humans, and is considered a potential anti-cryptococcal target because inositol pyrophosphates and polyphosphates are critical for metabolic adaptation to host environments and the pathogenicity of *C. neoformans*^27^,^36^. Here we found that, in addition to lacking virulence, the *ipk1*Δ mutants exhibited pleiotropic phenotypes. Deletion of *IPK1* increased capsule production, but reduced melanin and urease production. Its deletion also rendered cells to be defective in sexual differentiation and hypersensitive to high temperature and multiple stresses, and enhances susceptibility to multiple antifungal drugs (Supplementary Fig. 4).

### Biological functions of pathogenicity-related kinases

**Figure 5:**
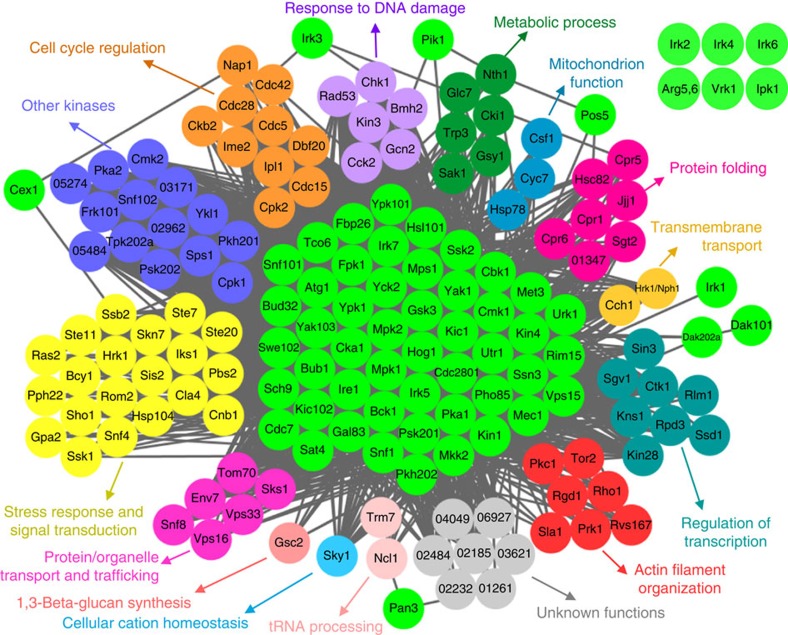
Network-based functional relationships among 63 pathogenicity-related kinases. Gene-function network analysis of the 63 pathogenicity-related kinases in *C. neoformans* by CryptoNet (http://www.inetbio.org/cryptonet)^17^. The functional correlation network was drawn based on 100 candidate genes that are predicted to have functional relationships with the 63 pathogenicity-related kinases by CryptoNet. Genes in this pathogenicity network were classified by the information of their Gene Ontology (GO) term or predicted biological functions listed in Supplementary Data 5. Six kinases (Arg5,6, Ipk1, Irk2, Irk4, Irk6, Vrk1) did not have any functionally related genes in CryptoNet.

We employed a genome-scale co-functional network for *C. neoformans*, CryptoNet (http://www.inetbio.org/cryptonet)^17^ to search for proteins functionally linked to the pathogenicity-related kinases, and thus construct a functional network of the pathogenicity-related kinome of *C. neoformans.* We did this by using previously reported information on *C. neoformans* and the Gene Ontology terms of corresponding kinase orthologs and its interacting proteins in *S. cerevisiae* and other fungi (Fig. 5). This analysis suggested that pathogenicity-related kinases have a role in a variety of different biological and physiological processes, as shown in Fig. 5 and Supplementary Data 5.

Among pathogenicity-related kinases, kinases involved in the cell cycle and growth control were identified most frequently. These include *CDC7*, *MPS1*, *PIK1*, *CDC2801*, *MEC1*, *BUD32*, and *CKA1*. Cdc7 is an essential catalytic subunit of the Dbf4-dependent protein kinase that is required for firing of the replication of origin throughout the S phase in *S. cerevisiae*^37^. Cdc7 in *C. neoformans* has a kinase domain at the C terminus (292–669 aa) and an extended N-terminal region (Supplementary Fig. 5a). Deletion of the whole-kinase domain was not feasible, suggesting that Cdc7 is likely to be an essential gene in *C. neoformans* (Supplementary Data 1). However, we were able to construct partial *CDC7* deletion mutants by disrupting the N-terminal region and part of the kinase domain (1–418 aa) (Supplementary Fig. 5b). Unexpectedly, the remaining kinase domain (419–669 aa) was still expressed in this strain (Supplementary Fig. 5c).

**Figure 6:**
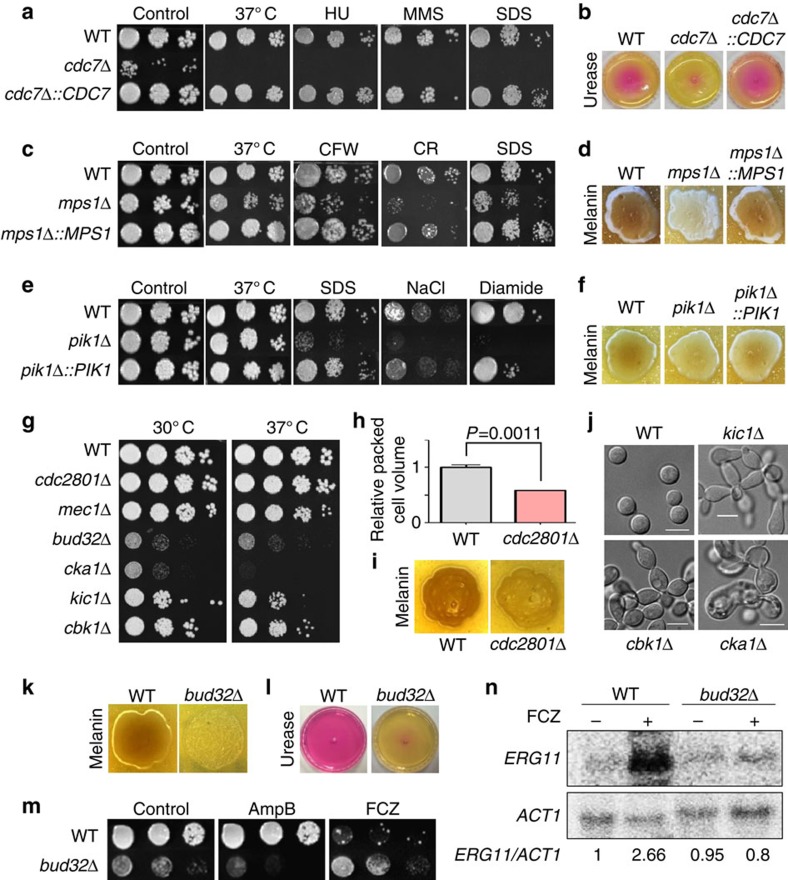
Kinases involved in growth and cell morphology of *Cryptococcus neoformans.* Various phenotypic tests were performed using the WT strain (H99S) and the following kinase mutants and complemented strains: (**a**,**b**) *cdc7*Δ (YSB2912), *cdc7*Δ*::CDC7* (YSB4356), (**c**,**d**) *mps1*Δ (YSB3632), *mps1*Δ*::MPS1* (YSB4351), (**e**,**f**) *pik1*Δ (YSB1493), *pik1*Δ*::PIK1* (YSB4360), (**g**–**n**) *cdc2801*Δ (YSB2370), *mec1*Δ (YSB3063), *cka1*Δ (YSB3051), *kic1*Δ (YSB2915), *cbk1*Δ (YSB2941), and *bud32*Δ (YSB1968) strains. (**a**,**c**,**e**,**m**) Cells were spotted on YPD medium containing the following chemicals at the indicated concentrations: 110 mM hydroxyurea (HU), 0.04% methyl methanesulphonate (MMS), 0.03% sodium dodecyl sulphate (SDS), 4 mg ml^−1^ calcofluor-white (CFW), 0.8% Congo red (CR), 1.5 M NaCl, 2 mM diamide, 1 μg ml^−1^ amphotericin B (AmpB), or 14 μg ml^−1^ fluconazole (FCZ). The cells were incubated at 30 °C and photographed after 3 days. For testing thermotolerance, each strain was spotted on YPD medium, incubated at the indicated temperature, and photographed after 3 days. (**b**,**l**) Urease production assay. Each strain was spotted on Christensen's agar media, incubated at 30 °C, and photographed after 3 days. (**d**,**i**,**k**) Melanin production assay. Each strain was spotted on Niger seed media containing 0.1% glucose, incubated at 37 °C, and photographed after 2–3 days. (**h**) The relative packed cell volume of *cdc2801*Δ mutants. The ratio was calculated from three biological replicates with three technical replicates normalized to the WT strain. The statistical significance was calculated by the Bonferroni's test for multiple comparison. Error bars indicate the s.e.m. (**j**) Cell morphology picture of WT H99S, *kic1*Δ, *cbk1*Δ and *cka1*Δ strains as observed by differential interference contrast microscopy at 16 h after inoculation in liquid YPD medium. Scale bars, 10 μm. (**n**) WT and *bud32*Δ strains grown at 30 °C to the logarithmic phase were treated with (+) or without (−) 10 μg ml^−1^ FCZ for 90 min, and total RNA was extracted. The expression levels were visualized by northern blotting and quantified using a phosphorimager (Fujifilm BAS-1500). The whole gel and phosphorimages were displayed on Supplementary Fig. 6d.

The partial *CDC7* deletion strain exhibited severe growth defects and high susceptibility to genotoxic and membrane-destabilizing agents (Fig. 6a) and was highly defective in urease production (Fig. 6b). All of these pleiotropic roles of Cdc7 are likely to affect the virulence of *C. neoformans*. Summarizing these findings, the whole-kinase domain of Cdc7 is essential for the growth and pathogenicity of *C. neoformans*.

Unexpectedly, two kinases that are essential in *S. cerevisiae*, Mps1 and Pik1, were successfully disrupted and shown to be involved in the virulence of *C. neoformans.* Mps1 is an essential cell cycle protein required for spindle pole body duplication and spindle checkpoint in *S. cerevisiae*^38^. The *C. neoformans mps1*Δ mutant exhibited growth defects (particularly at host temperature) and increased susceptibility to cell wall/membrane-perturbing agents (Fig. 6c), and was defective in melanin production (Fig. 6d). Collectively, these defects may cause the attenuated virulence in the *mps1*Δ mutants. Pik1 is a phosphatidylinositol 4-kinase that is known to control cytokinesis of *S. cerevisiae*^39^. Although the *pik1*Δ mutant did not exhibit growth defects, it exhibited increased susceptibility to cell membrane, osmotic and oxidative stresses (Fig. 6e) and defects in melanin production (Fig. 6f), which may affect the virulence of *C. neoformans*. To further validate the role of Cdc7, Mps1 and Pik1, we generated complemented strains by re-integrating the wild-type gene into its native locus, and confirmed that all of their mutant phenotypes were restored (Fig. 6a–f).

CNAG_00415, which was previously named Cdc2801 (ref. 5), is homologous to *S. cerevisiae* Cdc28 that is an essential CDK1 family protein (BLASTp score 226.5, e-value: 3e^−63^). However, the closest Cdc28 orthologue in *C. neoformans* is CNAG_01664 (BLASTp score 384.2, e-value: 1e^−110^), which was here named Cdc28. Given that we were not able to disrupt *CDC28* (Supplementary Data 1), Cdc28 could be an essential gene in *C. neoformans*. Although deletion of *CDC2801* did not affect the normal growth, the *cdc2801*Δ mutant was highly sensitive to flucytosine, which hampers RNA and DNA synthesis, suggesting that Cdc2801 might be involved in cell cycle control or DNA damage repair (Fig. 6g and Supplementary Fig. 5d). Furthermore, the *cdc2801*Δ mutant was highly defective in capsule and melanin production (Fig. 6h,i), which may affect infectivity.

Mec1 is required for cell cycle checkpoint, telomere maintenance and silencing and DNA damage repair in *S. cerevisiae*^40^. Reflecting these roles, deletion of *MEC1* increased cellular sensitivity to genotoxic agents in *C. neoformans* (Supplementary Fig. 5e). Deletion of *MEC1* did not cause any lethality or growth defects in *C. neoformans* (Fig. 6g), as was the case in *C. albicans*^41^. Cka1 and Cka2 are catalytic α-subunits of protein kinase CK2, which have essential roles in growth and proliferation of *S. cerevisiae*; deletion of both kinases causes lethality^42^. *C. neoformans* appears to have a single protein (*CKA1*) that is orthologous to both Cka1 and Cka2. Deletion of *CKA1* severely affected the growth of *C. neoformans* (Fig. 6g). Notably, the *cka1*Δ mutant showed elongated, swollen, abnormal cell morphology, which is comparable to, but rather distinct from that of two kinase mutants in the RAM pathway (*cbk1*Δ and *kic1*Δ) showing mostly elongated pseudohyphal growth (Fig. 6j). Cbk1 and Kic1 control the cellular polarity and morphology of *C. neoformans*^43^. Our insect-killing and STM-based murine infectivity assays revealed the involvement of Cbk1 and Kic1 in virulence and infectivity, respectively (Fig. 3).

Bud32 is also required for growth, potentially through involvement of tRNA modification. In *S. cerevisiae*, Bud32 is a component of the highly conserved KEOPS/EKC protein complex that is required for N^6^-threonylcarbamoyladenosine (t^6^A) tRNA modification^44^. The *bud32*Δ mutants exhibited defective growth under basal and most of the stress conditions (Supplementary Fig. 6a), produced smaller amounts of capsule, melanin and urease (Fig. 6k,l and Supplementary Fig. 6b), and were defective in mating (Supplementary Fig. 6c). However, the *bud32*Δ mutant exhibited an increased resistance to fluconazole (Fig. 6m). Interestingly, we found that deletion of *BUD32* abolished the induction of *ERG11* upon sterol depletion by fluconazole treatment (Fig. 6n), suggesting a potential role of Bud32 in ergosterol gene expression and sterol biosynthesis in *C. neoformans*.

Kinases involved in nutrient metabolism are also involved in the pathogenicity of *C. neoformans*. In *S. cerevisiae*, Arg5,6 is synthesized as a single protein and is subsequently processed into two separate enzymes for biosynthesis of ornithine, an arginine intermediate^45^. Consistent with this, we found that the *arg5,6*Δ mutant was auxotrophic for arginine (Supplementary Fig. 7a). In *S. cerevisiae*, *MET3* encodes an enzyme required for biosynthesis of homocysteine, cysteine and methionine^46^,^47^. Indeed, the *met3*Δ mutant was found to be auxotrophic for both methionine and cysteine (Supplementary Fig. 7b). Notably, *arg5,6*Δ and *met3*Δ mutants did not exhibit growth defects in nutrient-rich media, but exhibited severe growth defects at high temperature and under various stress conditions (Supplementary Fig. 7c), which may contribute to their virulence defects.

### Retrograde vacuole trafficking affects fungal pathogenicity

**Figure 7:**
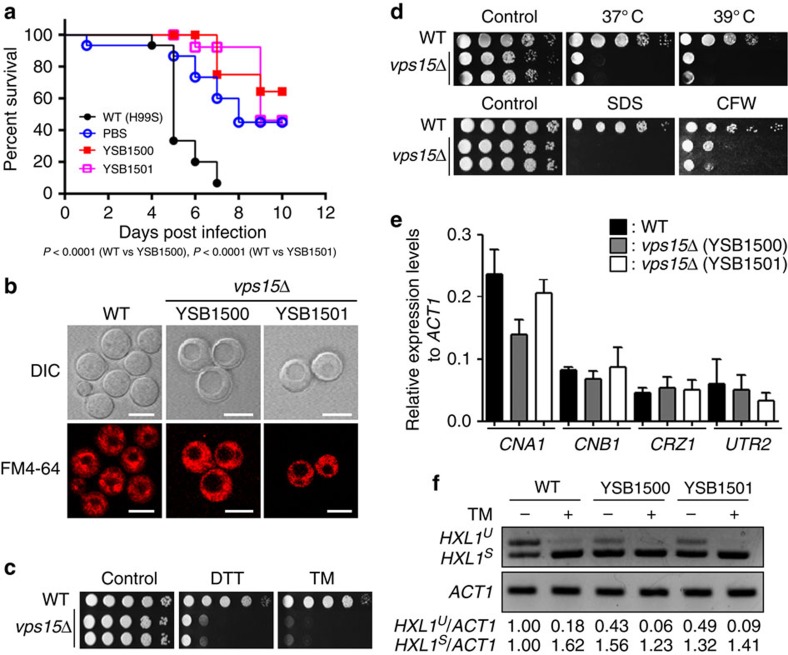
Retrograde vacuole trafficking controls the pathogenicity of *Cryptococcus neoformans*. Various tests were performed using WT strain (H99S) and *vps15*Δ mutants (YSB1500 and YSB1501) (**a**) Vps15 is required for virulence of *C. neoformans*. WT and PBS were used as positive and negative virulence controls, respectively. (**b**) *vps15*Δ mutants display enlarged vacuole morphology. Scale bars,10 μm. (**c**) *vps15*Δ mutants show significant growth defect under ER stresses. Overnight cultured cells were serially diluted tenfold (undiluted to 10^4^-fold dilution), spotted on the solid YPD medium containing 15 mM dithiothreitol (DTT) or 0.3 μg ml^−1^ tunicamycin (TM), further incubated at 30 °C for 3 days, and photographed. (**d**) *vps15*Δ mutants show significant growth defects at high temperature and under cell membrane/wall stresses. Overnight cultured cells were spotted on the YPD medium and further incubated at indicated temperature (upper panel) or the YPD medium containing 0.03% SDS or 5 mg ml^−1^ calcofluor white (CFW) and further incubated at 30 °C (lower panel). Plates were photographed after 3 days. (**e**) Vps15 is not involved in the regulation of the calcineurin pathway in *C. neoformans*. For quantitative RT–PCR (qRT–PCR), RNA was extracted from three biological replicates with three technical replicates of WT and *vps15*Δ mutants. *CNA1*, *CNB1*, *CRZ1*, *UTR2* expression levels were normalized by *ACT1* expression levels as controls. Error bars represent s.e.m. (**f**) Vps15 negatively regulates the *HXL1* splicing. For RT–PCR, total RNA was extracted from WT and *vps15*Δ mutants and cDNA was synthesized. *HXL1* and *ACT1*-specific primer pairs were used for RT–PCR. This experiment was repeated twice and one representative experiment is presented. The whole-gel images were displayed on Supplementary Fig. 6e.

In the rice blast fungus, *Magnaporthe oryzae*, retromer mediating retrograde protein trafficking is critical for autophagy-dependent plant infection^48^. In *C. neoformans*, the ESCRT complex-mediated vacuolar sorting process is involved in virulence because some virulence factors such as capsule and melanin need to be secreted^49^,^50^. However, the role of endosome-to-Golgi retrograde transport in the virulence of *C. neoformans* has not previously been characterized. Here we discovered that deletion of CNAG_02680, encoding a *VPS15* orthologue involved in the vacuolar sorting process, significantly reduced virulence (Fig. 7a). This result is consistent with the finding that mutation of *VPS15* also attenuates virulence of *C. albicans*^51^, strongly suggesting that the role of Vps15 in fungal virulence is evolutionarily conserved. In *S. cerevisiae*, Vps15 constitutes the vacuolar protein sorting complex (Vps15/30/34/38) that mediates endosome-to-Golgi retrograde protein trafficking^52^.

Similar to the *vps15*Δ null mutant in *C. albicans*^51^, the *C. neoformans vps15*Δ mutant also exhibited highly enlarged vacuole morphology (Fig. 7b). Defects in retrograde vacuole trafficking can cause extracellular secretion of an endoplasmic reticulum (ER)-resident chaperon protein, Kar2 (ref. 51). Supporting this, we found that *vps15*Δ mutants were highly susceptible to ER stress agents, such as dithiothreitol (DTT) and tunicamycin (TM) (Fig. 7c). Growth defects at 37 °C strongly attenuated the virulence and infectivity of the *vps15*Δ mutant (Fig. 7d). This high temperature sensitivity of the *vps15*Δ mutant may result from increased cell wall and membrane instability (Fig. 7d). In *C. albicans*, impaired retrograde transport in the *vps15*Δ mutant also causes cell wall stress, activating the calcineurin signalling pathway by transcriptionally upregulating *CRZ1*, *CHR1* and *UTR2* (ref. 51). In *C. neoformans*, however, we did not observe such activation of signalling components in the calcineurin pathway of the *vps15*Δ mutant (Fig. 7e). Expression levels of *CNA1*, *CNB1*, *CRZ1* and *UTR2* in the *vps15*Δ mutant were equivalent to those in the wild-type strain. In *C. neoformans*, cell wall integrity is also governed by the unfolded protein response (UPR) pathway^22^. Previously we demonstrated that activation of the UPR pathway through Ire1 kinase results in an unconventional splicing event in *HXL1* mRNA, which subsequently controls an ER stress response and adaptation^22^. Notably, we found that cells with the *VPS15* deletion were more enriched with spliced *HXL1* mRNA (*HXL1*^*s*^) under basal conditions than the wild-type strain, indicating that the UPR pathway may be activated instead of the calcineurin pathway in *C. neoformans* when retrograde vacuole trafficking is perturbed (Fig. 7f).

### Unknown pathogenicity-related kinases in *C. neoformans*

**Figure 8:**
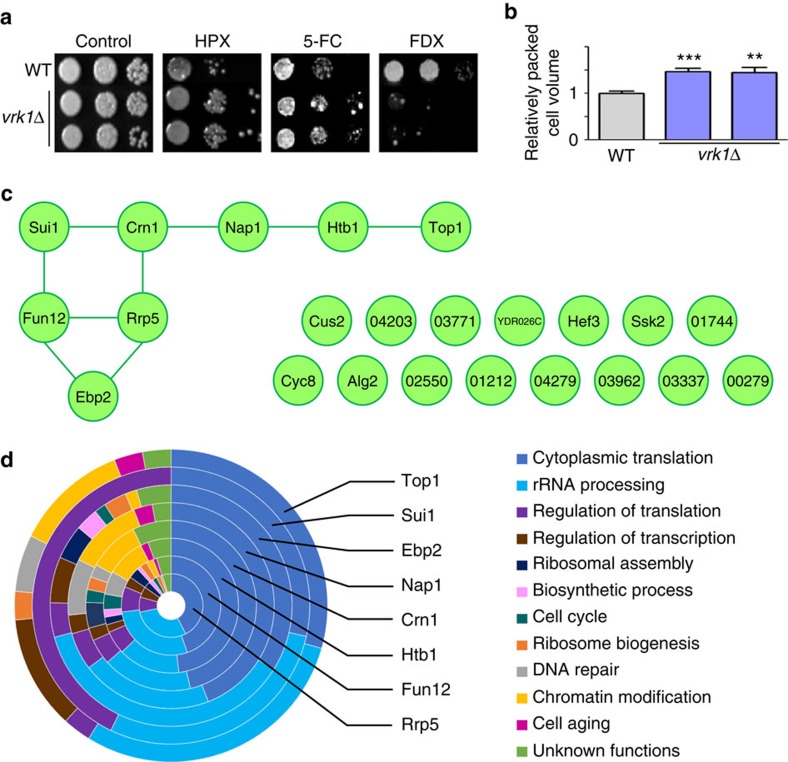
Phenotypic traits and phosphoproteomic analysis of the *vrk1*Δ mutant in *Cryptococcus neoformans*. (**a**) WT strain (H99S) and *vrk1*Δ mutants (YSB2216 and YSB2217) grown overnight were serially diluted tenfold (10^2^ to 10^4^-fold dilution), spotted on solid YPD medium containing 2.5 mM H_2_O_2_ (HPX), 600 μg ml^−1^ flucytosine (5-FC) or 1 μg ml^−1^ fludioxonil (FDX), further incubated for 3 days at 30 °C and were photographed. (**b**) *vrk1*Δ mutants show increased capsule production. Their relative packed cell volume ratio was calculated from three biological replicates with three technical replicates with normalization to that of WT strain. Based on the Bonferroni's multiple comparison test, double and triple asterisks indicate *P* values of 0.0038 and 0.0004, respectively. Error bars mean s.e.m. (**c**) Functional network relationship among the Vrk1 phospho-target proteins using CryptoNet. Significant differences were observed in 23 proteins by statistical analysis of the phosphopeptides qualified in WT and/or *vrk1*Δ mutant strains listed in Supplementary Data 6. The functional relationship of each protein was connected by a line. (**d**) Multi-layered pie-chart for summarizing gene-function network analysis of the Vrk1 target proteins by CryptoNet. The network analysis was based on 100 candidate genes that are predicted to have functional relationship with the indicated eight Vrk1-regulated target proteins. Genes were classified by the information of their Gene Ontology (GO) term or predicted biological functions listed in Supplementary Data 5.

Eight of the 63 pathogenicity-related kinases did not appear to have apparent orthologs in model yeasts and were thereby named virulence-related kinase (Vrk1) or infectivity-related kinase 1–7 (Irk1–7) (Figs 2,3). Particularly, we paid attention to Vrk1 (CNAG_06161) because its deletion reduced the virulence of *C. neoformans* in the insect host model (Supplementary Fig. 3) and diminished infectivity in the murine host model (Fig. 3a). Surprisingly, deletion of *VRK1* increased cellular resistance to hydrogen peroxide and capsule production (Fig. 8a,b), which may enhance the virulence potential of *C. neoformans*. In contrast, increased 5-flucytosine resistance and fludioxonil susceptibility were observed in the *vrk1*Δ mutant (Fig. 8a). Based on our phenome clustering data, Vrk1 was not clearly grouped with other kinases (Fig. 2), suggesting that Vrk1 may constitute a novel pathogenic kinase network in *C. neoformans*.

To gain further insight into the regulatory mechanism of Vrk1, we performed comparative phosphoproteomic analysis of the wild-type and *vrk1*Δ strains to identify Vrk1-specific phospho-target proteins. A total of 823 phosphopeptides corresponding to 380 non-redundant phosphorylated proteins were qualitatively identified by label-free liquid chromatography–tandem mass spectrometry (LC–MS/MS) (Supplementary Data 6). Among these, 29 phosphorylated peptides, which exhibited more than a 1.5-fold difference in the normalized relative phosphopeptide abundance between wild-type and *vrk1*Δ strains with statistical significance (*P*<0.05 by Student's *t*-test), were considered as potential Vrk1 substrate (Supplementary Data 6), corresponding to the 23 non-redundant proteins. Only 14 of these have known yeast orthologues (Supplementary Data 6 and Fig. 8c).

To gain further insight into Vrk1-dependent functional networks, we used CryptoNet to search for any proteins that were functionally linked to the 23 Vrk1-regulated target proteins and Vrk1 itself, and constructed the correlation networks for those proteins (Supplementary Data 5). Of these, 15 proteins did not have meaningful connections with any known proteins, but eight proteins (Ebp2, Fun12, Rrp5, Sui1, Crn1, Nap1, Htb1 and Top1) were found to be functionally connected. Among a variety of potential biological functions connected to Vrk1 and its substrates, translation and ribosomal RNA processing were mostly over-represented, suggesting that Vrk1 could be involved in the protein translation process, either directly or indirectly.

## Discussion

In this study, we attempted to functionally analyse the *C. neoformans* kinome web, which is predicted to consist of 183 kinases. We constructed 264 signature-tagged mutant strains representing 129 kinases to systematically analyse their *in vitro* and *in vivo* phenotypic traits in *C. neoformans*. The success level for mutant construction (129 out of 183 (70%)) was lower than that for transcription factors that we previously reported (155 out of 178 (87%))(ref. 6), probably because among fungi, kinases are generally much more evolutionarily conserved than transcription factors, and a greater number of essential or growth-related genes appeared to exist (Supplementary Data 7; Supplementary Fig. 8).

Our systematic analysis revealed that *C. neoformans* appears to have a significantly redundant set of essential kinases, but also contain a distinct set of essential kinases. This is not unexpected finding based on other systematic fungal kinome analyses. Even in two different very well validated strains of *S. cerevisiae* (S288C and Σ1278b), marked differences in which genes are essential are observed^53^. Furthermore, several kinases essential in *Aspergillus nidulans* (Wee1, NimA, Vps15 and Cka1) are not essential in *N. crassa*^54^,^55^. Among these, Vps15 and Cka1 were also not essential in *C. neoformans*, although deletion of these genes resulted in growth defects, while Wee1 (CNAG_03171) and NimA (CNAG_04148) orthologs appeared to be essential in *C. neoformans* (Supplementary Data 1). In addition, Ire1 is not essential in *C. neoformans*, *A. fumigatus*, *S. pombe* and *S. cerevisiae*, although it is essential in *A. nidulans*, *A. niger* and *F. graminearum*.

Here we showed that *CDC7* is likely to be essential in *C. neoformans*, but partial deletion of the gene region encoding the N terminus (1–418 aa) was feasible and led to the reduced virulence. *C. neoformans* Cdc7 has a kinase domain at the C terminus and an extended N-terminal region, which is distinct from *S. cerevisiae* Cdc7 having a similar kinase domain with a very short N-terminal region (Supplementary Fig. 5a). The extended N-terminal region of *C. neoformans* Cdc7 is longer than that of other fungal Cdc7 orthologues (Supplementary Fig. 5a), although its biological meaning remains unknown. At this point, it remains unclear how the truncated kinase domain of *CDC7* in the partial *CDC7* deletion strain is expressed. Due to the N-terminal deletion, the closest promoter region for expression of the C-terminal truncated kinase domain is the constitutively active *ACT1* promoter in the *NAT* selection marker (980 bp upstream; Supplementary Fig. 5b). In fact, our quantitative reverse transcription PCR (qRT–PCR) analysis showed that expression levels of the undeleted C-terminal region of *CDC7* in the partial *CDC7* deletion strain were higher than those in the wild-type strain (Supplementary Fig. 5c), suggesting that the distant *ACT1* promoter may induce expression of the C-terminally truncated kinase domain of *CDC7*. However, it needs to be further characterized whether such a truncated transcript of *CDC7* may be properly translated or not.

Mps1 and Pik1 do not appear to be universally essential in all fungi. Despite the essential role of Mps1 in *S. cerevisiae*^38^, Mps1 orthologue, Mph1, is not essential in *S. pombe* because it is required for the spindle assembly checkpoint, but not for spindle pole body duplication^56^. Therefore, Mps1 may also have non-essential roles in *C. neoformans* like in *S. pombe*. The Pik1 kinase is essential in *S. cerevisiae* and *S. pombe* by controlling cytokinesis as well as protein secretion and vacuole trafficking^57^. *PIK1* has been successfully deleted and shown to be involved in virulence of *C. neoformans*^58^. Pik1 orthologs are not found in *Aspergillus*, suggesting that the essential role of Pik1 may vary among fungi^54^. In *A. nidulans*, Stt4 is proposed to replace the essential role of Pik1 (ref. 54). *C. neoformans* contains a single ortholog (CNAG_04335) for Stt4 and we have failed to obtain its deletion mutants, suggesting that Stt4 may replace the essential role of Pik1 in *C. neoformans*.

Although the current study is the first genome-wide functional kinome study in a human fungal pathogen, similar studies have been performed in non-pathogenic filamentous fungal models (*N. crassa*^55^ and *A. nidulans*^54^,^59^) and a plant pathogenic filamentous fungus (*F. graminearum*^35^). One common phenotypic trait analysed in this and other studies is the role of developmental processes. We found that a total of 42 kinases were involved in the filamentous growth of *C. neoformans* during mating and that some of these have common roles in the developmental process in other fungi (Supplementary Fig. 9). Such common fungal developmental regulators include kinases in the HOG and pheromone-responsive MAPK pathways (Hog1, Ste7 and Ste11) and Kic102. The role of the two MAPK pathways in the fungal developmental process has already been reported^60^. However, we first reported here the role of Kic102 in the filamentous growth of *C. neoformans*. Deletion of *KIC102* significantly increased the filamentous growth of *C. neoformans* (Supplementary Fig. 10), suggesting that Kic102 is a major negative regulator of the developmental process of the pathogen. In contrast, Kic102 orthologues function as positive regulators in developmental processes of other fungi, including *A. nidulans*^54^,^59^, *F. graminearum*^35^ and *N. crassa*^55^. Therefore, Kic102 and its orthologues appear to play divergent roles in the developmental process among fungi.

Phenotypic clustering of kinases based on collected phenome database revealed several novel kinases. However, there are two major limitations in this phenotypic clustering analysis. First, kinases that are oppositely regulated in the same pathway cannot be clustered. Second, a kinase that regulates a subset of phenotypes governed by a signalling pathway may not be clustered with its upstream kinases; this is the case of the Hog1-regulated kinase 1 (CNAG_00130; Hrk1). Although we previously demonstrated that Hrk1 is regulated by Hog1 (ref. 19), Hrk1 and Hog1 are not clustered together as Hrk1 regulates only subsets of Hog1-dependent phenotypes. Phospholipid flippase kinase 1 (Fpk1) is another such example. In *S. cerevisiae*, Fpk1 is inhibited by direct phosphorylation by Ypk1 (ref. 61); as expected, Fpk1 and Ypk1 were not clustered together (Fig. 2). To address the functional correlation between Fpk1 and Ypk1, we performed epistatic analyses by constructing and analysing *FPK1* overexpression strains constructed in the *ypk1*Δ and wild-type strain backgrounds. Surprisingly, overexpression of *FPK1* partly restored normal growth, resistance to some stresses and amphotericin B in *ypk1*Δ mutants (Supplementary Fig. 10). These results suggest that Fpk1 could be one of the downstream targets of Ypk1 and may be positively regulated by Ypk1 in *C. neoformans*.

In addition, we identified 63 pathogenicity-related kinases, which cover a wide range of biological functions of *C. neoformans*. Current therapeutic options for treatment of cryptococcosis are highly limited owing to problems of incomplete drug efficacy, cytotoxicity and emergence of drug resistant strains for clinically available antifungal drugs^3^,^62^. Therefore, the development of novel therapeutic targets and agents is in high demand. From this perspective, pathogenicity-related kinases are very attractive because kinases are highly favoured ‘druggable' targets in past and on-going clinical trials^9^. Our comprehensive *in vitro* phenome data provide significant clues regarding how the pathogenicity-related kinases affect the virulence of *C. neoformans* (Supplementary Table 1). Of the 63 pathogenicity-related kinases, 30 were required for growth at the host's physiological temperature, 52 were involved in the production of at least one of three major virulence factors (capsule, melanin and urease), and 57 were are involved in stress responses and adaptation, suggesting that these phenotypic traits are highly correlated with the pathogenicity of *C. neoformans*. Notably, 28 of these play pleiotropic roles in thermotolerance, virulence factor production, and stress responses. However, the *in vitro* phenotypic traits of four kinases (Irk2, Irk3, Psk201 and Dak202A) did not reflect their roles in pathogenicity. The *irk2*Δ mutant showed increased urease production and the *irk3*Δ mutant did not exhibit any discernible *in vitro* phenotypes. The *psk201*Δ mutant showed increased capsule production. Furthermore, the *dak202A*Δ mutant displayed increased production of capsule and melanin and resistance to oxidative stresses. Therefore, the four kinases could have specific *in vivo* roles during host infection by *C. neoformans*.

Based on antifungal drug susceptibility analysis of the mutant kinase library, a total 46, 50 and 43 kinases exhibited either increased or decreased susceptibility to amphotericin B, fluconazole or flucytosine, respectively, which are clinically available antifungal drugs (Supplementary Table 2). Of the pathogenicity-related kinases, we identified 32 kinases whose deletion affected susceptibility to amphotericin B, 33 to fluconazole, and 31 to flucytosine, respectively. Particularly, the following eight kinases appeared to promote resistance to all three antifungal drugs: Ypk1, Vps15, Gsk3, Utr1, Ipk1, Ire1, Cbk1 and Cdc7. Interestingly, they all promoted the pathogenicity of *C. neoformans* (Fig. 3), suggesting that these eight kinases could be targeted for development of both monotherapy and combination therapy with known antifungal drugs. Therefore, our kinome analysis of *C. neoformans* reveals potential narrow- and broad-spectrum anticryptococcal and antifungal drug targets.

## Methods

### Ethics statement

Animal care and all experiments were conducted in accordance with the ethical guidelines of the Institutional Animal Care and Use Committee of Yonsei University. The Yonsei University Institutional Animal Care and Use Committee approved all of the vertebrate studies.

### Construction of the *C. neoformans* kinase mutant library

Kinase mutant strains were constructed in the *C. neoformans* serotype A H99S strain^63^,^64^ background. For gene-deletion through homologous recombination, gene-disruption cassettes containing the nourseothricin-resistance marker (*NAT*; nourseothricin acetyl transferase) with indicated signature-tagged sequences (Supplementary Data 8) were generated by using conventional overlap PCR or *NAT* split marker/double-joint (DJ) PCR strategies as previously reported^18^,^26^. All primers used in this study are listed in Supplementary Data 8. In the first round of PCR, the 5′- and 3′-flanking regions for the targeted kinase genes were amplified with primer pairs L1/L2 and R1/R2, respectively, by using H99S genomic DNA as a template. For the overlap PCR, the whole *NAT* marker was amplified with the primers M13Fe (M13 forward extended) and M13Re (M13 reverse extended) by using a pNAT-STM plasmid containing the *NAT* gene with each unique signature-tagged sequence. For the split marker/DJ-PCR, the split 5′- and 3′-regions of the *NAT* marker were amplified with primer pairs M13Fe/NSL and M13Re/NSR, respectively, with the plasmid pNAT-STM. In the second round of overlap PCR, the kinase gene-disruption cassettes were amplified with primers L1 and R2 by using the combined first round PCR products as templates. In the second round of split marker/DJ-PCR, the 5′- and 3′-regions of *NAT-*split gene-disruption cassettes were amplified with primer pairs L1/NSL and R2/NSR, respectively, by using combined corresponding first round PCR products as templates. For transformation, the H99S strain was cultured overnight at 30 °C in the 50 ml yeast extract-peptone-dextrose (YPD) medium, pelleted and re-suspended in 5 ml of distilled water. Approximately 200 μl of the cell suspension was spread on YPD solid medium containing 1 M sorbitol and further incubated at 30 °C for 3 h. The PCR-amplified gene disruption cassettes were coated onto 600 μg of 0.6 μm gold microcarrier beads (Bio-Rad) and biolistically introduced into the cells by using particle delivery system (PDS-100, Bio-Rad). The transformed cells were further incubated at 30 °C for recovery of cell membrane integrity and were scraped after 4 h. The scraped cells were transferred to the selection medium (YPD solid plate containing 100 μg ml^−1^ nourseothricin; YPD+NAT). Stable nourseothricin-resistant (NAT^r^) transformants were selected through more than two passages on the YPD+NAT plates. All NAT^r^ strains were confirmed by diagnostic PCR with each screening primer listed in Supplementary Data 8. To verify accurate gene deletion and the absence of any ectopic integration for each gene-disruption cassette, Southern blot analysis was performed for all of the kinase mutants. To validate a mutant phenotype and to exclude any unlinked mutational effects, we constructed more than two independent deletion strains for each kinase mutant. Some kinases that had been previously reported by others were independently deleted here with unique signature-tagged markers to perform parallel *in vitro* and *in vivo* phenotypic analysis (Supplementary Data 8). When two independent kinase mutants exhibited inconsistent phenotypes (known as inter-isolate inconsistency), we attempted to generate more than three mutants.

### Construction of mutant complemented strains

To confirm the phenotypes observed in the *pik1*Δ, *mps1*Δ and *cdc7*Δ mutants, the corresponding complemented strains were generated. To generate *mps1*Δ*::MPS1* complemented strains, a DNA fragment containing the promoter, terminator, and open reading frame (ORF) of *MPS1* was amplified using primer pair B7488/J343 and cloned into pGEM-T-easy vector to generate the plasmid pGEM-MPS1. After sequencing to identify a clone with no errors, the *MPS1* insert was subcloned into the plasmid pJAF12 containing the neomycin/G418-resistant marker (*NEO*) to generate the plasmid pJAF12-MPS1. The NdeI-digested linearized pJAF12-MPS1 was biolistically introduced into the *mps1*Δ mutants (YSB3632 and YSB3633). To generate the *cdc7*Δ*::CDC7* complemented strains, a genomic fragment of the *CDC7* gene containing the promoter, ORF, and terminator, was amplified by using the primer pair J331/J332. The amplified *CDC7* gene product was cloned into a plasmid pGEM-T-easy vector to generate the plasmid pGEM-CDC7. After sequencing to determine any errors, the *CDC7* gene insert was subcloned into the plasmid pJAF12 to generate the plasmid pJAF12-CDC7. The pJAF12-CDC7 was linearized by restriction digestion with NsiI and was biolistically introduced into the *cdc7*Δ mutants (YSB2911 and YSB2912). To construct the *pik1*Δ*::PIK1* complemented strains, a DNA fragment containing the promoter, terminator, and ORF of *PIK1* was cloned as follows. The promoter and 5′-exon region of *PIK1* was amplified using the primer pair B7507/J339. The 3′-exon region of *PIK1* and terminator was amplified using the primer pair J338/J340. Each amplified DNA fragment was cloned into plasmid pGEM-T-easy vector to generate the plasmids pGEM-PIK1L and pGEM-PIK1R, respectively. After sequencing, the KpnI-digested 3′-exon region of *PIK1* and terminator was subcloned into pGEM-PIK1L to produce the plasmid pGEM-PIK1LR. The *PIK1* gene insert was subcloned using NotI into the plasmid pJAF12 to generate the plasmid pJAF12-PIK1. The AatII-digested linearized pJAF12-PIK1 was then biolistically introduced into the *pik1*Δ mutants (YSB1493). A diagnostic PCR was then performed to confirm the targeted integration of each linearized plasmid into the corresponding native locus.

### Construction of *FPK1* overexpression strains

To construct the *FPK1* constitutive overexpression strain, the native promoter of *FPK1* was replaced with histone H3 promoter using an amplified homologous recombination cassette (Supplementary Fig. 10a). Primer pairs L1/OEL2 and OER1/PO were used for amplification of the 5′-flaking region and 5′-coding region, respectively, of *FPK1* in the first round of PCR. The *NEO-H3* promoter region was amplified with the primer pair B4017/B4018. At the second-round PCR, the 5′ region of the P_*H3*_:*FPK1* cassette was amplified by DJ-PCR using the mixed templates of the 5′-flaking region of *FPK1* and the *NEO-H3* promoter region with the primer pair L1/GSL (primers are listed in Supplementary Data 8). The 3′-region of the P_*H3*_:*FPK1* cassette was similarly amplified by using the mixed templates of the 3′-flaking region of *FPK1* and the *NEO-H3* promoter region with a primer pair GSR/PO. Then, the combined split P_*H3*_:*FPK1* cassettes were introduced into the wild-type strain H99S and the *ypk1*Δ mutant (YSB1736) by biolistic transformation. Stable transformants selected on YPD medium containing G418 were screened by diagnostic PCR with a primer pair (SO/B79). The correct genotype was verified by Southern blotting using a specific probe amplified by PCR with primers L1/PO. Overexpression of *FPK1* was verified by northern blot analysis using a specific northern blot probe amplified by PCR with primers NP1 and PO (Supplementary Fig. 10b,c).

### Growth and chemical susceptibility test

To analyse the growth and chemical susceptibility of the kinase mutant library, *C. neoformans* cells grown overnight at 30 °C were serially diluted tenfold (1 to 10^4^) and spotted on YPD plates containing the indicated concentrations of chemical agents as follows: sorbitol for osmotic stress and NaCl and KCl for cation/salt stresses under either glucose-rich (YPD) or glucose-starved (YPD without dextrose; YP) conditions; hydrogen peroxide (H_2_O_2_), *tert*-butyl hydroperoxide (an organic peroxide), menadione (a superoxide anion generator), diamide (a thiol-specific oxidant) for oxidative stress; cadmium sulphate (CdSO_4_) for toxic heavy metal stress; methyl methanesulphonate and hydroxyurea for genotoxic stress; sodium dodecyl sulphate (SDS) for membrane destabilizing stress; calcofluor white and Congo red for cell wall destabilizing stress; TM and DTT for ER stress and reducing stress; fludioxonil, fluconazole, amphotericin B, flucytosine for antifungal drug susceptibility. Cells were incubated at 30 °C and photographed post-treatment from day 2 to day 5. To test the growth rate of each mutant at distinct temperatures, YPD plates spotted with serially diluted cells were incubated at 25, 30, 37, and 39 °C, and photographed after 2–4 days.

### Mating assay

To examine the mating efficiency of each kinase mutant, the *MAT*α kinase mutant was co-cultured with serotype A *MAT***a** wild-type strain KN99**a** as a unilateral mating partner. Each α and **a** strain was cultured in YPD medium at 30 °C for 16 h, pelleted, washed and resuspended with distilled water. The resuspended α and **a** cells were mixed at equal concentrations (10^7^ cells per ml) and 5 μl of the mixture was spotted on V8 mating media (pH 5). The mating plate was incubated at room temperature in the dark for 7 to 14 days and was observed weekly.

### *In vitro* virulence-factor production assay

Capsule production was examined qualitatively by India ink staining as previously described^14^. To measure the capsule production levels quantitatively by Cryptocrit, each kinase mutant was grown overnight in YPD medium at 30 °C, spotted onto Dulbecco's Modified Eagle's solid medium, and incubated at 37 °C for 2 days for capsule induction. The cells were scraped, washed with phosphate buffered saline (PBS), fixed with 10% of formalin solution, and washed again with PBS. The cell concentration was adjusted to 3 × 10^8^ cells per ml for each mutant and 50 μl of the cell suspension was injected into microhaematocrit capillary tubes (Kimble Chase) in triplicates. All tubes were placed in an upright vertical position for 3 days to precipitate cells by gravity. The packed cell volume ratio was measured by calculating the ratio of the lengths of the packed cell phase to the total phase (cells plus liquid phases). The relative packed cell volume ratio was calculated by normalizing the packed cell volume ratio of each mutant with that of the wild-type strain. Statistical differences in relative packed cell volume ratios were determined by one-way analysis of variance tests employing the Bonferroni correction method by using the Prism 6 (GraphPad) software. To examine melanin production, each kinase mutant was grown overnight in YPD medium at 30 °C; 5 μl of each culture was spotted on Niger seed media containing 0.1% or 0.2% glucose, incubated at 37 °C and photographed after 3–4 days. For kinase mutants showing growth defects at 37 °C, the melanin and capsule production were assessed at 30 °C. To examine urease production, each kinase mutant was grown in YPD medium at 30 °C overnight, washed with distilled water, and an equal number of cells (5 × 10^4^) was spotted onto Christensen's agar media. The plates were incubated for 2–3 days at 30 °C and photographed.

### The insect-based *in vivo* virulence assay

For each tested *C. neoformans* strain, we randomly selected a group of 15 *Galleria mellonella* caterpillars in the final instar larval stage with a body weight of 200–300 mg, which arrived within 7 days from the day of shipment (Vanderhorst Inc.). Each *C. neoformans* strain was grown overnight at 30 °C in YPD liquid medium, washed three times with PBS, pelleted and resuspended in PBS at equal concentrations (10^6^ cells per ml). A total of 4,000 *C. neoformans* cells in a 4-μl volume per larva was inoculated through the second to last prolegs by using a 100-μl Hamilton syringe equipped with a 10 μl-size needle and a repeating dispenser (PB600-1, Hamilton). PBS was injected as a non-infectious control. Infected larvae were placed in petri dishes in a humidified chamber, incubated at 37 °C, and monitored daily. Larvae were considered dead when they showed a lack of movement upon touching. Larvae that pupated during experiments were censored for statistical analysis. Survival curves were illustrated using the Prism 6 software (GraphPad). The Log-rank (Mantel-Cox) test was used for statistical analysis. We examined two independent mutant strains for each kinase mutant.

### The STM-based murine infectivity assay

For the high-throughput murine infectivity test, a group of kinase mutant strains with the *NAT* selection marker containing 45 unique signature-tags (a total of four groups) was pooled. The *ste50*Δ and *hxl1*Δ mutants were used as virulent and avirulent control strains respectively, as previously reported^6^,^22^. Each group of the kinase mutant library was grown at 30 °C in YPD medium for 16 h separately and washed three times with PBS. The concentration of each mutant was adjusted to 10^7^ cells per ml and 50 μl of each sample was pooled into a tube. For preparation of the input genomic DNA of each kinase mutant pool, 200 μl of the mutant pool was spread on YPD plate, incubated at 30 °C for 2 days, and scraped. For preparation of the output genomic DNA samples, 50 μl of the mutant pool (5 × 10^5^ cells per mouse) was infected into seven-week-old female A/J mice (Jackson Laboratory) through intranasal inhalation. The infected mice were sacrificed with an overdose of Avertin 15 days post-infection, their lungs were recovered and homogenized in 4 ml PBS, spread onto the YPD plates containing 100 μg ml^−1^ of chloramphenicol, incubated at 30 °C for 2 days, and scraped. Total genomic DNA was extracted from scraped input and output cells by the cetyltrimethyl ammonium bromide DNA extraction method. Quantitative PCR was performed with the tag-specific primers listed in Supplementary Data 8 using MyiQ2 Real-Time PCR detection system (Bio-Rad). The STM score was calculated as previously described^6^. Briefly, relative changes in genomic DNA amounts were calculated by the 2^−ΔΔCT^ method to determine the STM score. The mean fold changes in input verses output samples were calculated in Log score (Log_2_ 2^−(*C*t,Target−*C*t,Actin)output−(*C*t,Target−*C*t,Actin)input^). We monitored two independent mutants for each kinase.

### Vacuole staining

To visualize vacuole morphology, the wild-type H99S strain and *vps15*Δ strains (YSB1500 and YSB1501) were cultured in liquid YPD medium at 30 °C for 16 h. FM4–64 dye (Life Technologies) was added to each culture at a final concentration of 10 μM and further incubated at 30 °C for 30 min. The cells were pelleted by centrifugation, resuspended with fresh liquid YPD medium, and further incubated at 30 °C for 30 min. The cells were pelleted again, washed three times with PBS, and resuspended in 1 ml of PBS. On the glass slide, 10 μl of the cells and 10 μl of mounting solution (Biomeda) were mixed and spotted. The glass slides were observed by confocal microscope (Olympus BX51 microscope).

### TiO_2_ enrichment-based phosphoproteomics

To identify the phosphorylated targets of Vrk1 on a genome-wide scale, the H99S and *vrk1*Δ mutant strains were incubated in YPD liquid medium at 30 °C for 16 h, sub-cultured into 1 l of fresh YPD liquid medium, and further incubated at 30 °C until it approximately reached an optical density at 600 nm (OD_600_) of 0.9. Each whole-cell lysate was prepared with lysis buffer containing 50 mM Tris-Cl (pH 7.5), 1% sodium deoxycholate, 5 mM sodium pyrophosphate, 0.2 mM sodium orthovanadate, 50 mM sodium fluoride (NaF), 0.1% sodium dodecyl sulphate, 1% Triton X-100, 0.5 mM phenylmethylsulfonyl fluoride and 2.5 × protease inhibitor cocktail solution (Merck Millipore). The protein concentration of each cell lysate was estimated using a Pierce BCA protein kit (Life Technologies). Sulfhydryl bonds between cysteine residues in protein lysates were reduced by incubating 10 mg of total protein lysate with 10 mM DTT at room temperature for 1 h and then alkylated with 50 mM iodoacetamide in the dark at room temperature for 1 h. These samples were treated again with 40 mM DTT at room temperature for 30 min and digested using trypsin (Sequencing grade trypsin, Promega) at an enzyme: substrate ratio of 1:50 (w/w) with overnight incubation at 37 °C. The trypsin-digested protein lysates were then purified with Sep-Pak C18 columns (Waters Corporation), lyophilized and stored at −80 °C. Phosphopeptides were enriched using TiO_2_Mag Sepharose beads (GE Healthcare) and lyophilized for LC–MS/MS. Mass spectrometric analyses were performed using a Q Exactive Hybrid Quadrupole-Orbitrap mass spectrometer (Thermo Scientific) equipped with Dionex U 3000 RSLC nano high-performance liquid chromatography system, a nano-electrospray ionization source and fitted with a fused silica emitter tip (New Objective). All phosphopeptide samples were reconstituted in solution A (water/acetonitrile (98:2, v/v), 0.1% formic acid), and then injected into an LC-nano electrospray ionisation-MS/MS system. Samples were first trapped on a Acclaim PepMap 100 trap column (100 μm i.d. × 2 cm, nanoViper C_18_, 5 μm particle size, 100 Å pore size, Thermo Scientific) and washed for 6 min with 98% solution A at a flow rate of 4 μl min^−1^, and then separated on an Acclaim PepMap 100 capillary column (75 μm i.d. × 15 cm, nanoViper C_18_, 3 μm particle size, 100 Å pore size, Thermo Scientific) at a flow rate of 400 nl min^−1^. Peptides were analysed with a gradient of 2 to 35% solution B (water/acetonitrile (2:98, v/v), 0.1% formic acid) over 90 min, 35 to 90% over 10 min, followed by 90% for 5 min, and finally 5% for 15 min. Resulting peptides were electrosprayed through a coated silica tip (PicoTip emitter) at an ion spray voltage of 2,000 eV. MS/MS spectra were searched against the *C. neoformans* var. *grubii* H99S protein database (http://www.uniprot.org) using the MASCOT (version 2.4, Matrixscience) search algorithms through the Proteome Discoverer platform (version 1.4, Thermo Scientific) for assigning peptides. The MS and MS/MS tolerance was set at 15 p.p.m. and 0.8 Da, respectively. The PhosphoRS node was used to calculate individual site probabilities for phosphorylated peptides. The following search parameters were applied: cysteine carbamidomethylation as fixed modifications, methionine oxidation and serine/threonine/tyrosine phosphorylation as variable modifications, and two missed trypsin cleavages were allowed to identify the peptide. Peptide identification was filtered by a 1% false discovery rate cut-off. PhosphoRS probability score ≥0.75 threshold was used to confirm the site of phosphorylation. Spectral counts were used to estimate relative phosphopeptide abundance between the wild-type and *vrk1*Δ mutant strains. The data were further normalized by dividing each ratio (*vrk1*Δ per wild-type) by the median value of the ratio of each LC–MS/MS run, resulting in normalized relative phosphopeptide abundance. The Student's *t*-test was used to assess the statistically significant difference between the samples.

### ER stress assay

To monitor the ER stress-mediated UPR induction, the H99S and *vps15*Δ mutant strains were incubated in YPD at 30 °C for 16 h, sub-cultured into fresh YPD liquid medium, and further incubated at 30 °C until they reached the early-logarithmic phase (OD_600_=0.6). The cells were treated with 0.3 μg ml^−1^ TM for 1 h. The cell pellets were immediately frozen with liquid nitrogen and then lyophilized. Total RNAs were extracted using easy-BLUE (Total RNA Extraction Kit, Intron Biotechnology) and subsequently complementary DNA (cDNA) was synthesized using an MMLV reverse transcriptase (Invitrogen). *HXL1* splicing patterns (UPR-induced spliced form of *HXL1* (*HXL1*^*S*^) and unspliced form of *HXL1* (*HXL1*^*U*^)) were analysed by PCR using cDNA samples of each strain and primers (B5251 and B5252) listed in Supplementary Data 8.

### Expression analysis

To measure the expression level of *ERG11*, the H99S strain and *bud32*Δ mutants were incubated in liquid YPD medium at 30 °C for 16 h and sub-cultured into fresh liquid YPD medium. When the cells reach the early-logarithmic phase (OD_600_=0.6), the culture was divided into two samples: one was treated with fluconazole for 90 min and the other was not treated. The cell pellets were immediately frozen with liquid nitrogen and then lyophilized. Total RNA was extracted and northern blot analysis was performed with the total RNA samples for each strain. For qRT–PCR analysis of genes involved in the calcineurin pathway, the H99S strain and *vps15*Δ mutants were incubated in liquid YPD medium at 30 °C for 16 h and were sub-cultured into fresh liquid YPD medium until they reach to the early-logarithmic phase (OD_600_=0.8). The cells were then pelleted, immediately frozen with liquid nitrogen, and lyophilized. After total RNA was extracted, cDNA was synthesized using RTase (Thermo Scientific). *CNA1*, *CNB1*, *CRZ1*, *UTR2* and *ACT1*-specific primer pairs (B7030 and B7031, B7032 and B7033, B7034 and B7035, B7036 and B7037, B679 and B680, respectively) (Supplementary Data 8) were used for qRT–PCR.

### The kinase phenome clustering

*In vitro* phenotypic traits of each kinase mutant were scored with the following qualitative scale: −3 (strongly sensitive or defective), −2 (moderately sensitive or defective), −1 (weakly sensitive or defective), 0 (wild-type-like), +1 (weakly resistant or enhanced), +2 (moderately resistant or enhanced) and +3 (strongly resistant or enhanced). The excel file containing the phenotype scores of each kinase mutant was loaded by Gene-E software (http://www.broadinstitute.org/cancer/software/GENE-E/) and then kinase phenome clustering was drawn using one minus Pearson correlation.

### *Cryptococcus* kinome web-database

For public access to the phenome and genome data for the *C. neoformans* kinase mutant library, the *Cryptococcus* Kinase Phenome Database was developed (http://kinase.cryptococcus.org/). Genome sequences of the H99 strain were downloaded from Broad Institute (http://www.broadinstitute.org/annotation/genome/cryptococcus_neoformans/MultiHome.html), and incorporated into the standardized genome data warehouse in the Comparative Fungal Genomics Platform database (CFGP 2.0; http://cfgp.snu.ac.kr/)^65^. Classification of kinases was performed by using the hidden Markov model-based sequence profiles of SUPERFAMILY (version 1.73; ref. 66). A total of 64 family identifiers belonging to 38 superfamilies were used to predict putative kinases (Supplementary Table 3). In addition, the sequence profiles of Kinomer (version 1.0; refs 11, 12) and the Microbial Kinome^67^ were used to supplement the kinase prediction. Information from genome annotation of *C. neoformans* var. *grubii* H99 and protein domain predictions of InterProScan^68^ was also adopted to capture the maximal extent of possible kinase-encoding genes. For each gene, results from the eight bioinformatics programs are also provided to suggest clues for gene annotations. In addition, results from SUPERFAMILY, Kinomer and Microbial Kinome were displayed for supporting robustness of the prediction. If a gene has an orthologue in *C. neoformans* var. *neoformans* JEC21, a link to the KEGG database was also provided. Finally, transcript expression and phenotype screening data are shown along with the above-mentioned information. To browse genomic data in context to important biological features, the Seoul National University genome browser (SNUGB; http://genomebrowser.snu.ac.kr/)^69^ was integrated into the *Cryptococcus* kinase phenome database. In kinase browser, a direct link to the SNUGB module was provided for each gene. The *Cryptococcus* kinase phenome database was developed by using MySQL 5.0.81 (source code distribution) for database management and PHP 5.2.6 for web interfaces. The web-based user interface is served through the Apache 2.2.9 web server.

### Data availability

All the data related to mutant construction were deposited in the publicly accessible *C. neoformans* kinome database (http://kinase.cryptococcus.org) that we constructed for the current study. All kinase mutant strains were deposited in the Korean Culture Collection of Microorganisms in Korea (http://www.kccm.or.kr/) and in the Center of Host-Microbial Interactions at Duke University in USA. Other data that support the findings of this study are available from the corresponding author on request.

## Additional information

**How to cite this article:** Lee, K.-T. *et al.* Systematic functional analysis of kinases in the fungal pathogen *Cryptococcus neoformans*. *Nat. Commun.* 7:12766 doi: 10.1038/ncomms12766 (2016).

## Supplementary Material

Supplementary Figures, Tables and References Supplementary Figures 1-10, Supplementary Tables 1-3 and Supplementary References

Supplementary Data 1 List of Cryptococcus neoformans kinases

Supplementary Data 2 List of the pathogenic fungal kinases predicted based on the kinase domain database search

Supplementary Data 3 Pathogenicity-related kinases in Cryptococcus neoformans.

Supplementary Data 4 Complete phenome heat map of C. neoformans kinases

Supplementary Data 5 Raw data of the network analysis from Cryptonet

Supplementary Data 6 Detailed information regarding phosphopeptides identified in wide-type and/or vrk1Δ mutant strain of Cryptococcus neoformans

Supplementary Data 7 BLAST matrix analysis of Cryptococcus neoformans kinases

Supplementary Data 8 List of primers used for constructing kinase mutant library.

## References

[b1] Park B. J. *et al.* Estimation of the current global burden of cryptococcal meningitis among persons living with HIV/AIDS. AIDS 23, 525–530 (2009). 19182676 10.1097/QAD.0b013e328322ffac

[b2] Lin X. & Heitman J. The biology of the *Cryptococcus neoformans* species complex. Annu. Rev. Microbiol. 60, 69–105 (2006). 16704346 10.1146/annurev.micro.60.080805.142102

[b3] Perfect J. R. *et al.* Clinical practice guidelines for the management of cryptococcal disease: 2010 update by the infectious diseases society of America. Clin. Infect. Dis. 50, 291–322 (2010). 20047480 10.1086/649858 PMC5826644

[b4] Idnurm A. *et al.* Deciphering the model pathogenic fungus *Cryptococcus neoformans*. Nat. Rev. Microbiol. 3, 753–764 (2005). 16132036 10.1038/nrmicro1245

[b5] Liu O. W. *et al.* Systematic genetic analysis of virulence in the human fungal pathogen *Cryptococcus neoformans*. Cell 135, 174–188 (2008). 18854164 10.1016/j.cell.2008.07.046 PMC2628477

[b6] Jung K. W. *et al.* Systematic functional profiling of transcription factor networks in *Cryptococcus neoformans*. Nat. Commun. 6, 6757 (2015). 25849373 10.1038/ncomms7757 PMC4391232

[b7] Maier E. J. *et al.* Model-driven mapping of transcriptional networks reveals the circuitry and dynamics of virulence regulation. Genome Res. 25, 690–700 (2015). 25644834 10.1101/gr.184101.114 PMC4417117

[b8] Cohen P. The regulation of protein function by multisite phosphorylation-a 25 year update. Trends Biochem. Sci. 25, 596–601 (2000). 11116185 10.1016/s0968-0004(00)01712-6

[b9] Rask-Andersen M., Masuram S. & Schioth H. B. The druggable genome: evaluation of drug targets in clinical trials suggests major shifts in molecular class and indication. Annu. Rev. Pharmacol. Toxicol. 54, 9–26 (2014). 24016212 10.1146/annurev-pharmtox-011613-135943

[b10] Cheek S., Zhang H. & Grishin N. V. Sequence and structure classification of kinases. J. Mol. Biol. 320, 855–881 (2002). 12095261 10.1016/s0022-2836(02)00538-7

[b11] Miranda-Saavedra D. & Barton G. J. Classification and functional annotation of eukaryotic protein kinases. Proteins 68, 893–914 (2007). 17557329 10.1002/prot.21444

[b12] Martin D. M., Miranda-Saavedra D. & Barton G. J. Kinomer v. 1.0: a database of systematically classified eukaryotic protein kinases. Nucleic Acids Res. 37, D244–D250 (2009). 18974176 10.1093/nar/gkn834 PMC2686601

[b13] Bahn Y. S., Geunes-Boyer S. & Heitman J. Ssk2 mitogen-activated protein kinase kinase kinase governs divergent patterns of the stress-activated Hog1 signaling pathway in *Cryptococcus neoformans*. Eukaryot. Cell 6, 2278–2289 (2007). 17951522 10.1128/EC.00349-07 PMC2168243

[b14] Bahn Y. S., Hicks J. K., Giles S. S., Cox G. M. & Heitman J. Adenylyl cyclase-associated protein Aca1 regulates virulence and differentiation of *Cryptococcus neoformans* via the cyclic AMP-protein kinase A cascade. Eukaryot. Cell 3, 1476–1491 (2004). 15590822 10.1128/EC.3.6.1476-1491.2004 PMC539029

[b15] Bahn Y. S., Kojima K., Cox G. M. & Heitman J. Specialization of the HOG pathway and its impact on differentiation and virulence of *Cryptococcus neoformans*. Mol. Biol. Cell 16, 2285–2300 (2005). 15728721 10.1091/mbc.E04-11-0987 PMC1087235

[b16] Bahn Y. S., Kojima K., Cox G. M. & Heitman J. A unique fungal two-component system regulates stress responses, drug sensitivity, sexual development, and virulence of *Cryptococcus neoformans*. Mol. Biol. Cell 17, 3122–3135 (2006). 16672377 10.1091/mbc.E06-02-0113 PMC1483045

[b17] Kim H. *et al.* Network-assisted genetic dissection of pathogenicity and drug resistance in the opportunistic human pathogenic fungus *Cryptococcus neoformans*. Sci. Rep. 5, 8767 (2015). 25739925 10.1038/srep08767 PMC4350084

[b18] Kim M. S., Kim S. Y., Yoon J. K., Lee Y. W. & Bahn Y. S. An efficient gene-disruption method in *Cryptococcus neoformans* by double-joint PCR with *NAT*-split markers. Biochem. Biophys. Res. Commun. 390, 983–988 (2009). 19852932 10.1016/j.bbrc.2009.10.089

[b19] Kim S. Y. *et al.* Hrk1 plays both Hog1-dependent and -independent roles in controlling stress response and antifungal drug resistance in *Cryptococcus neoformans*. PLoS ONE 6, e18769 (2011). 21533251 10.1371/journal.pone.0018769 PMC3076434

[b20] Kojima K., Bahn Y. S. & Heitman J. Calcineurin, Mpk1 and Hog1 MAPK pathways independently control fludioxonil antifungal sensitivity in *Cryptococcus neoformans*. Microbiology 152, 591–604 (2006). 16514140 10.1099/mic.0.28571-0

[b21] Maeng S. *et al.* Comparative transcriptome analysis reveals novel roles of the Ras and cyclic AMP signaling pathways in environmental stress response and antifungal drug sensitivity in *Cryptococcus neoformans*. Eukaryot. Cell 9, 360–378 (2010). 20097740 10.1128/EC.00309-09 PMC2837985

[b22] Cheon S. A. *et al.* Unique evolution of the UPR pathway with a novel bZIP transcription factor, Hxl1, for controlling pathogenicity of *Cryptococcus neoformans*. PLoS Pathog. 7, e1002177 (2011). 21852949 10.1371/journal.ppat.1002177 PMC3154848

[b23] Jiang R. & Carlson M. The Snf1 protein kinase and its activating subunit, Snf4, interact with distinct domains of the Sip1/Sip2/Gal83 component in the kinase complex. Mol. Cell Biol. 17, 2099–2106 (1997). 9121458 10.1128/mcb.17.4.2099 PMC232057

[b24] Schuller H. J. Transcriptional control of nonfermentative metabolism in the yeast *Saccharomyces cerevisiae*. Curr. Genet. 43, 139–160 (2003). 12715202 10.1007/s00294-003-0381-8

[b25] Hu G., Cheng P. Y., Sham A., Perfect J. R. & Kronstad J. W. Metabolic adaptation in *Cryptococcus neoformans* during early murine pulmonary infection. Mol. Microbiol. 69, 1456–1475 (2008). 18673460 10.1111/j.1365-2958.2008.06374.x PMC2730461

[b26] Davidson R. C. *et al.* A PCR-based strategy to generate integrative targeting alleles with large regions of homology. Microbiology 148, 2607–2615 (2002). 12177355 10.1099/00221287-148-8-2607

[b27] Lev S. *et al.* Fungal inositol pyrophosphate IP_7_ is crucial for metabolic adaptation to the host environment and pathogenicity. mBio 6, e00531–00515 (2015). 26037119 10.1128/mBio.00531-15 PMC4453010

[b28] Chakraborty A., Kim S. & Snyder S. H. Inositol pyrophosphates as mammalian cell signals. Sci. Signal. 4, re1 (2011). 21878680 10.1126/scisignal.2001958 PMC3667551

[b29] Vlahakis A. & Powers T. A role for TOR complex 2 signaling in promoting autophagy. Autophagy 10, 2085–2086 (2014). 25426890 10.4161/auto.36262 PMC4502789

[b30] Lee H., Khanal Lamichhane A., Garraffo H. M., Kwon-Chung K. J. & Chang Y. C. Involvement of PDK1, PKC and TOR signalling pathways in basal fluconazole tolerance in *Cryptococcus neoformans*. Mol. Microbiol. 84, 130–146 (2012). 22339665 10.1111/j.1365-2958.2012.08016.x PMC3313003

[b31] Gerik K. J., Bhimireddy S. R., Ryerse J. S., Specht C. A. & Lodge J. K. PKC1 is essential for protection against both oxidative and nitrosative stresses, cell integrity, and normal manifestation of virulence factors in the pathogenic fungus *Cryptococcus neoformans*. Eukaryot. Cell 7, 1685–1698 (2008). 18689526 10.1128/EC.00146-08 PMC2568057

[b32] Kraus P. R., Fox D. S., Cox G. M. & Heitman J. The *Cryptococcus neoformans* MAP kinase Mpk1 regulates cell integrity in response to antifungal drugs and loss of calcineurin function. Mol. Microbiol. 48, 1377–1387 (2003). 12787363 10.1046/j.1365-2958.2003.03508.x PMC1635492

[b33] D'Souza C. A. *et al.* Cyclic AMP-dependent protein kinase controls virulence of the fungal pathogen *Cryptococcus neoformans*. Mol. Cell Biol. 21, 3179–3191 (2001). 11287622 10.1128/MCB.21.9.3179-3191.2001 PMC86952

[b34] Chang Y. C., Ingavale S. S., Bien C., Espenshade P. & Kwon-Chung K. J. Conservation of the sterol regulatory element-binding protein pathway and its pathobiological importance in *Cryptococcus neoformans*. Eukaryot. Cell 8, 1770–1779 (2009). 19749173 10.1128/EC.00207-09 PMC2772393

[b35] Wang C. *et al.* Functional analysis of the kinome of the wheat scab fungus *Fusarium graminearum*. PLoS Pathog. 7, e1002460 (2011). 22216007 10.1371/journal.ppat.1002460 PMC3245316

[b36] Li C. *et al.* Identification of a major IP_5_ kinase in *Cryptococcus neoformans* confirms that PP-IP_5_/IP_7_, not IP_6_, is essential for virulence. Sci. Rep. 6, 23927 (2016). 27033523 10.1038/srep23927 PMC4817067

[b37] Diffley J. F., Cocker J. H., Dowell S. J., Harwood J. & Rowley A. Stepwise assembly of initiation complexes at budding yeast replication origins during the cell cycle. J. Cell Sci. 19, (Suppl): 67–72 (1995). 10.1242/jcs.1995.supplement_19.9 8655649

[b38] Weiss E. & Winey M. The *Saccharomyces cerevisiae* spindle pole body duplication gene *MPS1* is part of a mitotic checkpoint. J. Cell Biol. 132, 111–123 (1996). 8567717 10.1083/jcb.132.1.111 PMC2120695

[b39] Zhou T., Aumais J. P., Liu X., Yu-Lee L. Y. & Erikson R. L. A role for Plk1 phosphorylation of NudC in cytokinesis. Dev. Cell 5, 127–138 (2003). 12852857 10.1016/s1534-5807(03)00186-2

[b40] Mills K. D., Sinclair D. A. & Guarente L. MEC1-dependent redistribution of the Sir3 silencing protein from telomeres to DNA double-strand breaks. Cell 97, 609–620 (1999). 10367890 10.1016/s0092-8674(00)80772-2

[b41] Legrand M., Chan C. L., Jauert P. A. & Kirkpatrick D. T. The contribution of the S-phase checkpoint genes *MEC1* and *SGS1* to genome stability maintenance in *Candida albicans*. Fungal Genet. Biol. 48, 823–830 (2011). 21511048 10.1016/j.fgb.2011.04.005 PMC3126902

[b42] Padmanabha R., Chen-Wu J. L., Hanna D. E. & Glover C. V. Isolation, sequencing, and disruption of the yeast *CKA2* gene: casein kinase II is essential for viability in *Saccharomyces cerevisiae*. Mol. Cell Biol. 10, 4089–4099 (1990). 2196445 10.1128/mcb.10.8.4089-4099.1990 PMC360927

[b43] Walton F. J., Heitman J. & Idnurm A. Conserved elements of the RAM signaling pathway establish cell polarity in the basidiomycete *Cryptococcus neoformans* in a divergent fashion from other fungi. Mol. Biol. Cell 17, 3768–3780 (2006). 16775005 10.1091/mbc.E06-02-0125 PMC1556378

[b44] Srinivasan M. *et al.* The highly conserved KEOPS/EKC complex is essential for a universal tRNA modification, t6A. EMBO J. 30, 873–881 (2011). 21183954 10.1038/emboj.2010.343 PMC3049205

[b45] Boonchird C., Messenguy F. & Dubois E. Determination of amino acid sequences involved in the processing of the ARG5/ARG6 precursor in *Saccharomyces cerevisiae*. Eur. J. Biochem. 199, 325–335 (1991). 1649049 10.1111/j.1432-1033.1991.tb16128.x

[b46] Cherest H., Nguyen N. T. & Surdin-Kerjan Y. Transcriptional regulation of the *MET3* gene of *Saccharomyces cerevisiae*. Gene 34, 269–281 (1985). 2989110 10.1016/0378-1119(85)90136-2

[b47] Ullrich T. C., Blaesse M. & Huber R. Crystal structure of ATP sulfurylase from *Saccharomyces cerevisiae*, a key enzyme in sulfate activation. EMBO J. 20, 316–329 (2001). 11157739 10.1093/emboj/20.3.316 PMC133462

[b48] Zheng W. H. *et al.* Retromer is essential for autophagy-dependent plant infection by the rice blast Fungus. PLoS Genet. 11, e1005704 (2015). 26658729 10.1371/journal.pgen.1005704 PMC4686016

[b49] Godinho R. M. *et al.* The vacuolar-sorting protein Snf7 is required for export of virulence determinants in members of the *Cryptococcus neoformans* complex. Sci. Rep. 4, 6198 (2014). 25178636 10.1038/srep06198 PMC4151102

[b50] Hu G. *et al.* *Cryptococcus neoformans* requires the ESCRT protein Vps23 for iron acquisition from heme, for capsule formation, and for virulence. Infect. Immun. 81, 292–302 (2013). 23132495 10.1128/IAI.01037-12 PMC3536123

[b51] Liu Y. *et al.* Role of retrograde trafficking in stress response, host cell interactions, and virulence of *Candida albicans*. Eukaryot. Cell 13, 279–287 (2014). 24363364 10.1128/EC.00295-13 PMC3910971

[b52] Stack J. H., Horazdovsky B. & Emr S. D. Receptor-mediated protein sorting to the vacuole in yeast: roles for a protein kinase, a lipid kinase and GTP-binding proteins. Annu. Rev. Cell Dev. Biol. 11, 1–33 (1995). 8689553 10.1146/annurev.cb.11.110195.000245

[b53] Dowell R. D. *et al.* Genotype to phenotype: a complex problem. Science 328, 469 (2010). 20413493 10.1126/science.1189015 PMC4412269

[b54] De Souza C. P. *et al.* Functional analysis of the *Aspergillus nidulans* kinome. PLoS ONE 8, e58008 (2013). 23505451 10.1371/journal.pone.0058008 PMC3591445

[b55] Park G. *et al.* Global analysis of serine-threonine protein kinase genes in *Neurospora crassa*. Eukaryot. Cell 10, 1553–1564 (2011). 21965514 10.1128/EC.05140-11 PMC3209061

[b56] He X., Jones M. H., Winey M. & Sazer S. Mph1, a member of the Mps1-like family of dual specificity protein kinases, is required for the spindle checkpoint in *S. pombe*. J. Cell Sci. 111, 1635–1647 (1998). 9601094 10.1242/jcs.111.12.1635

[b57] Park J. S., Steinbach S. K., Desautels M. & Hemmingsen S. M. Essential role for *Schizosaccharomyces pombe* *pik1* in septation. PLoS ONE 4, e6179 (2009). 19587793 10.1371/journal.pone.0006179 PMC2704394

[b58] Lee A. *et al.* Survival defects of *Cryptococcus neoformans* mutants exposed to human cerebrospinal fluid result in attenuated virulence in an experimental model of meningitis. Infect. Immun. 78, 4213–4225 (2010). 20696827 10.1128/IAI.00551-10 PMC2950369

[b59] Dyer P. S. & O'Gorman C. M. Sexual development and cryptic sexuality in fungi: insights from *Aspergillus* species. FEMS Microbiol. Rev. 36, 165–192 (2012). 22091779 10.1111/j.1574-6976.2011.00308.x

[b60] Zhao X., Mehrabi R. & Xu J. R. Mitogen-activated protein kinase pathways and fungal pathogenesis. Eukaryot. Cell 6, 1701–1714 (2007). 17715363 10.1128/EC.00216-07 PMC2043402

[b61] Roelants F. M., Baltz A. G., Trott A. E., Fereres S. & Thorner J. A protein kinase network regulates the function of aminophospholipid flippases. Proc. Natl Acad. Sci. USA 107, 34–39 (2010). 19966303 10.1073/pnas.0912497106 PMC2806694

[b62] Gullo F. P. *et al.* Cryptococcosis: epidemiology, fungal resistance, and new alternatives for treatment. Eur. J. Clin. Microbiol. Infect. Dis. 32, 1377–1391 (2013). 24141976 10.1007/s10096-013-1915-8

[b63] Perfect J. R., Ketabchi N., Cox G. M., Ingram C. W. & Beiser C. L. Karyotyping of *Cryptococcus neoformans* as an epidemiological tool. J. Clin. Microbiol. 31, 3305–3309 (1993). 8308124 10.1128/jcm.31.12.3305-3309.1993 PMC266409

[b64] Janbon G. *et al.* Analysis of the genome and transcriptome of *Cryptococcus neoformans* var. *grubii* reveals complex RNA expression and microevolution leading to virulence attenuation. PLoS Genet. 10, e1004261 (2014). 24743168 10.1371/journal.pgen.1004261 PMC3990503

[b65] Choi J. *et al.* CFGP 2.0: a versatile web-based platform for supporting comparative and evolutionary genomics of fungi and Oomycetes. Nucleic Acids Res. 41, D714–D719 (2013). 23193288 10.1093/nar/gks1163 PMC3531191

[b66] Wilson D. *et al.* SUPERFAMILY--sophisticated comparative genomics, data mining, visualization and phylogeny. Nucleic Acids Res. 37, D380–D386 (2009). 19036790 10.1093/nar/gkn762 PMC2686452

[b67] Kannan N., Taylor S. S., Zhai Y., Venter J. C. & Manning G. Structural and functional diversity of the microbial kinome. PLoS Biol. 5, e17 (2007). 17355172 10.1371/journal.pbio.0050017 PMC1821047

[b68] Hunter S. *et al.* InterPro in 2011: new developments in the family and domain prediction database. Nucleic Acids Res. 40, D306–D312 (2012). 22096229 10.1093/nar/gkr948 PMC3245097

[b69] Jung K. *et al.* SNUGB: a versatile genome browser supporting comparative and functional fungal genomics. BMC Genomics 9, 586 (2008). 19055845 10.1186/1471-2164-9-586 PMC2649115

[b70] Inglis D. O. *et al.* Literature-based gene curation and proposed genetic nomenclature for *Cryptococcus*. Eukaryot. Cell 13, 878–883 (2014). 24813190 10.1128/EC.00083-14 PMC4135733

