# Supplementary material for: Systematic functional analysis of kinases in the fungal pathogen Cryptococcus neoformans
Source: Nat Commun. 2016 Sep 28;7:12766. doi: 10.1038/ncomms12766 (PMC5052723; doi:10.1038/ncomms12766)
Supplement: Supplementary Figures, Tables and References — Supplementary Figures 1-10, Supplementary Tables 1-3 and Supplementary References [file ncomms12766-s1.pdf]

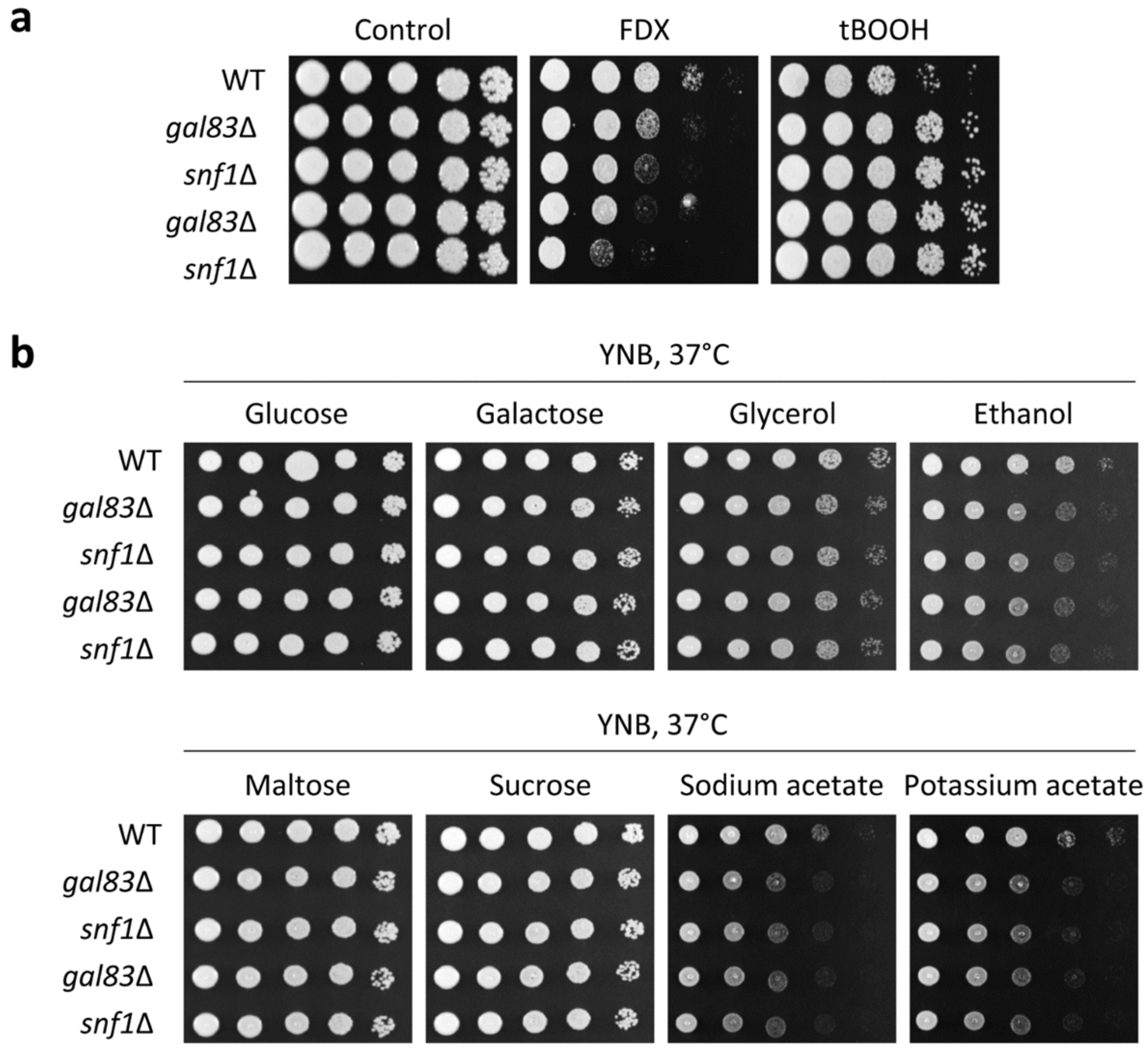

Supplementary Figure 1. *gal83*Δ and *snf1*Δ mutants share phenotypic traits in *Cryptococcus neoformans*. (**a**) WT strain (H99S) and *snf1*Δ (YSB2372 and YSB2373) and *gal83*Δ (YSB2415 and YSB2416) mutants were cultured for 16 hr in liquid YPD media at 30°C, spotted on YPD media containing 1 μg ml^{-1} fludioxonil (FDX) or 0.65 mM *tert*-butyl hydroperoxide (tBOOH), and further incubated at 30°C. Plates were photographed after 3 days. (**b**) WT (H99S), *snf1*Δ (YSB2372 and YSB2373) and *gal83*Δ (YSB2415 and YSB2416) mutants were cultured for 16 hr in liquid YPD media at 30°C, and spotted on YNB media containing 2% glucose, 2% galactose, 3% glycerol, 3% ethanol, 2% maltose, 2% sucrose, 2% sodium acetate, or 1% potassium acetate, and further incubated at 37°C. Plates were photographed after 2 days.

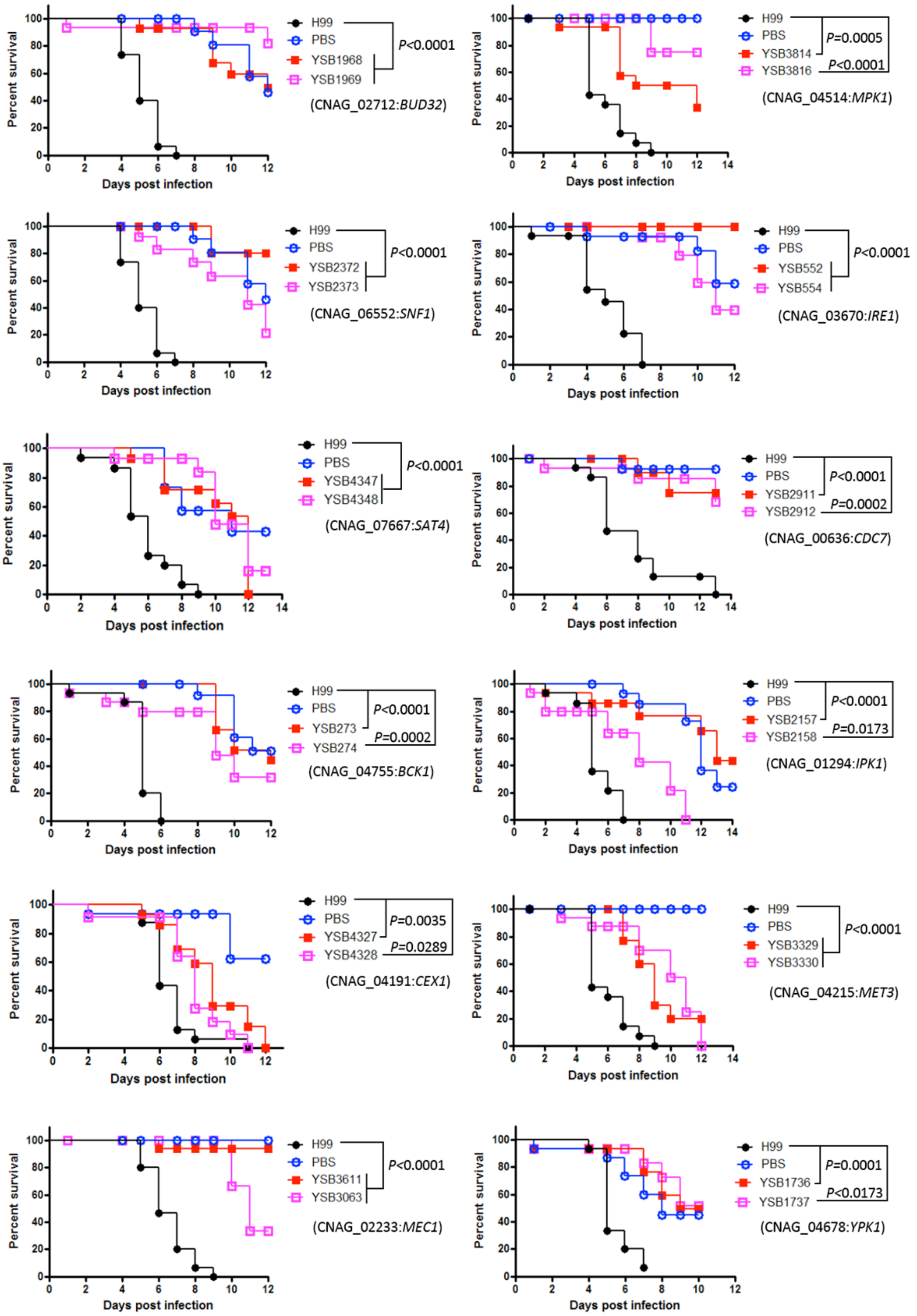

Percent survival
Days post infection
H99
PBS
YSB1968
YSB1969
P<0.0001
(CNAG_02712:BUD32)
YSB3814
YSB3816
P=0.0005
P<0.0001
(CNAG_04514:MPK1)
YSB2372
YSB2373
(CNAG_06552:SNF1)
YSB552
YSB554
(CNAG_03670:IRE1)
YSB4347
YSB4348
(CNAG_07667:SAT4)
YSB2911
YSB2912
P=0.0002
(CNAG_00636:CDC7)
YSB273
YSB274
(CNAG_04755:BCK1)
YSB2157
YSB2158
P=0.0173
(CNAG_01294:IPK1)
YSB4327
YSB4328
P=0.0035
P=0.0289
(CNAG_04191:CEX1)
YSB3329
YSB3330
(CNAG_04215:MET3)
YSB3611
YSB3063
(CNAG_02233:MEC1)
YSB1736
YSB1737
P=0.0001
P<0.0173
(CNAG_04678:YPK1)

Continued

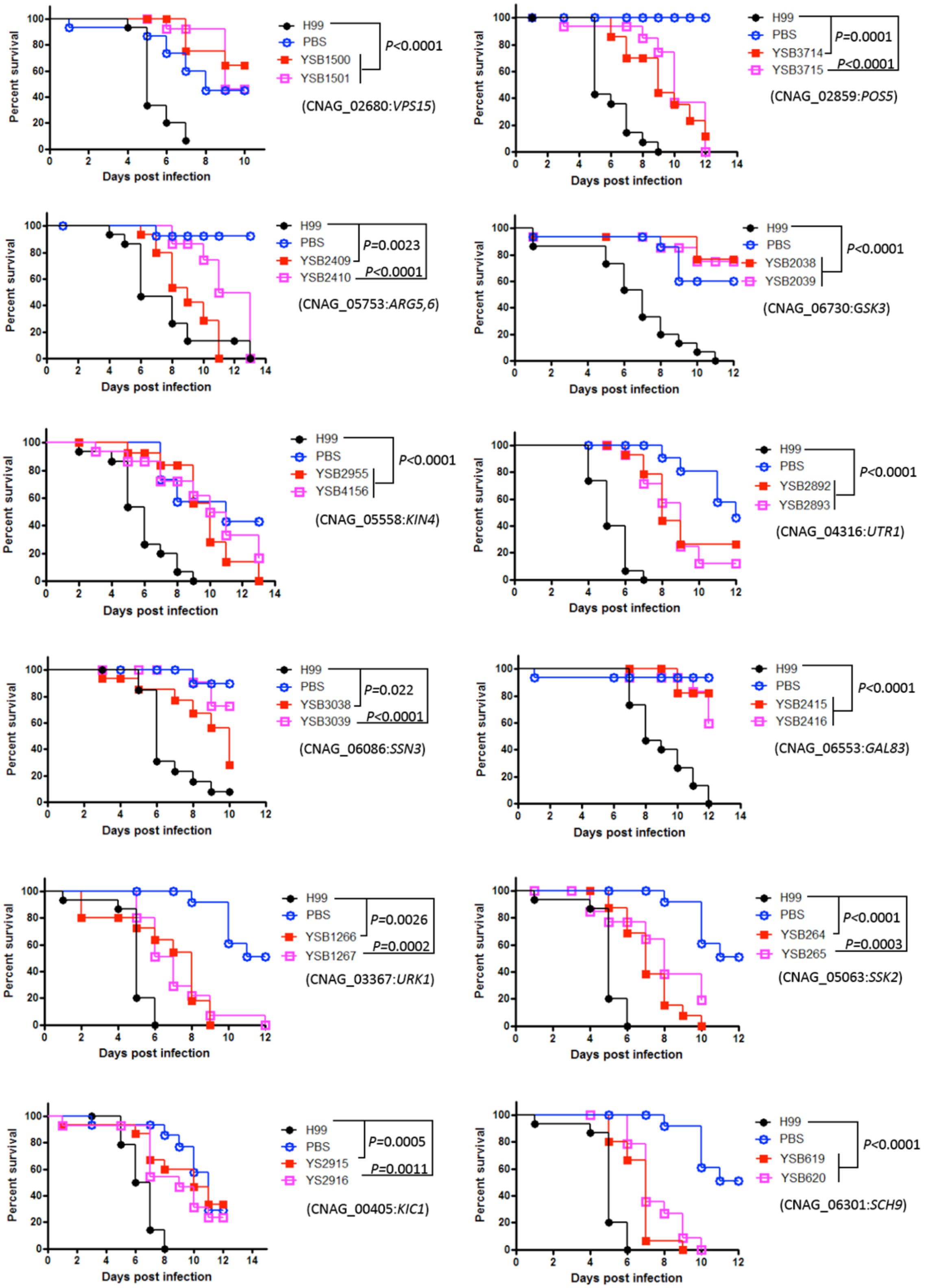
Percent survival
Days post infection
H99
PBS
YSB1500
YSB1501
P<0.0001
(CNAG_02680:VPS15)
H99
PBS
YSB3714
YSB3715
P=0.0001
P<0.0001
(CNAG_02859:POS5)
H99
PBS
YSB2409
YSB2410
P=0.0023
P<0.0001
(CNAG_05753:ARG5,6)
H99
PBS
YSB2038
YSB2039
P<0.0001
(CNAG_06730:GSK3)
H99
PBS
YSB2955
YSB4156
P<0.0001
(CNAG_05558:KIN4)
H99
PBS
YSB2892
YSB2893
P<0.0001
(CNAG_04316:UTR1)
H99
PBS
YSB3038
YSB3039
P=0.022
P<0.0001
(CNAG_06086:SSN3)
H99
PBS
YSB2415
YSB2416
P<0.0001
(CNAG_06553:GAL83)
H99
PBS
YSB1266
YSB1267
P=0.0026
P=0.0002
(CNAG_03367:URK1)
H99
PBS
YSB264
YSB265
P<0.0001
P=0.0003
(CNAG_05063:SSK2)
H99
PBS
YS2915
YS2916
P=0.0005
P=0.0011
(CNAG_00405:KIC1)
H99
PBS
YSB619
YSB620
P<0.0001
(CNAG_06301:SCH9)

Continued

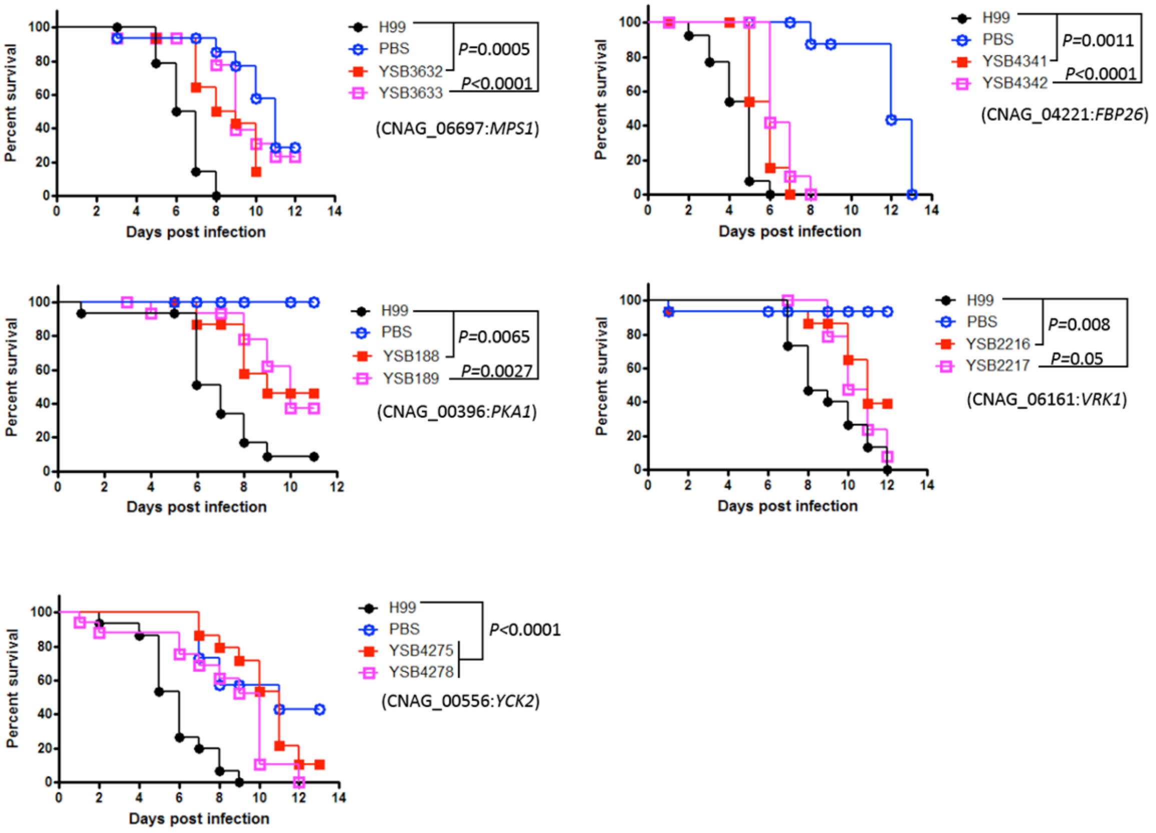

Supplementary Figure 2. Identification of the virulence-regulating kinases in *Cryptococcus neoformans* by using *Galleria mellonella* killing assay. Each tested *C. neoformans* strain was grown for 16 hr in liquid YPD medium, washed three times with phosphate-buffered saline (PBS), and inoculated into *G. mellonella* larva using 4,000 cells per larva (15 larvae per group). The infected larvae were incubated at 37°C in humidified chamber and monitored for their survival up to 14 days. Statistical analysis was performed using the Log-rank (Mantel-Cox) test. The survival data of two independent mutants for each kinase.

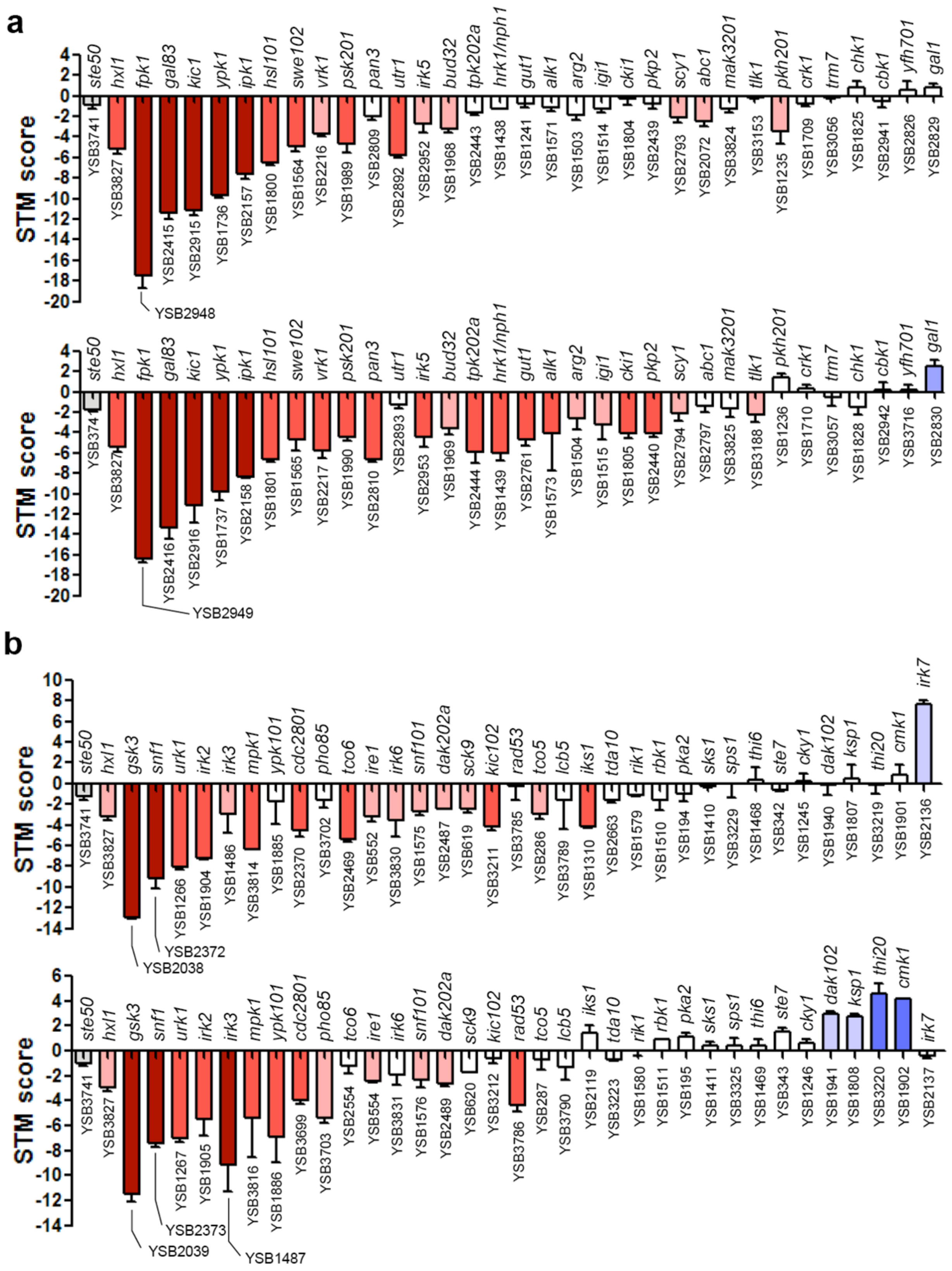
a
STM score
4 2 0 -2 -4 -6 -8 -10 -12 -14 -16 -18 -20
ste50 hxl1 fpk1 gal83 kic1 ypk1 ipk1 hsl101 swe102 vrk1 psk201 pan3 utr1 irk5 bud32 tpk202a hrk1/nph1 gut1 alk1 arg2 igi1 cki1 pkp2 scy1 abc1 mak3201 tlk1 pkh201 crk1 trm7 chk1 cbk1 yfh701 gal1
YSB3741 YSB3827 YSB2948 YSB2415 YSB2915 YSB1736 YSB2157 YSB1800 YSB1564 YSB2216 YSB1989 YSB2809 YSB2892 YSB2952 YSB1968 YSB2443 YSB1438 YSB1241 YSB1571 YSB1503 YSB1514 YSB1804 YSB2439 YSB2793 YSB2072 YSB3824 YSB3153 YSB1235 YSB1709 YSB3056 YSB1825 YSB2941 YSB2826 YSB2829
STM score
4 2 0 -2 -4 -6 -8 -10 -12 -14 -16 -18 -20
ste50 hxl1 fpk1 gal83 kic1 ypk1 ipk1 hsl101 swe102 vrk1 psk201 pan3 utr1 irk5 bud32 tpk202a hrk1/nph1 gut1 alk1 arg2 igi1 cki1 pkp2 scy1 abc1 mak3201 tlk1 pkh201 crk1 trm7 chk1 cbk1 yfh701 gal1
YSB374 YSB3827 YSB2949 YSB2416 YSB2916 YSB1737 YSB2158 YSB1801 YSB1565 YSB2217 YSB1990 YSB2810 YSB2893 YSB2953 YSB1969 YSB2444 YSB1439 YSB2761 YSB1573 YSB1504 YSB1515 YSB1805 YSB2440 YSB2794 YSB2797 YSB3825 YSB3188 YSB1236 YSB1710 YSB3057 YSB1828 YSB2942 YSB3716 YSB2830
b
STM score
10 8 6 4 2 0 -2 -4 -6 -8 -10 -12 -14
ste50 hxl1 gsk3 snf1 urk1 irk2 irk3 mpk1 ypk101 cdc2801 pho85 tco6 ire1 irk6 snf101 dak202a sck9 kic102 rad53 tco5 lcb5 iks1 tda10 rik1 rbk1 pka2 sks1 sps1 thi6 ste7 cky1 dak102 ksp1 thi20 cmk1 irk7
YSB3741 YSB3827 YSB2038 YSB2372 YSB1266 YSB1904 YSB1486 YSB3814 YSB1885 YSB2370 YSB3702 YSB2469 YSB552 YSB3830 YSB1575 YSB2487 YSB619 YSB3211 YSB3785 YSB286 YSB3789 YSB1310 YSB2663 YSB1579 YSB1510 YSB194 YSB1410 YSB3229 YSB1468 YSB342 YSB1245 YSB1940 YSB1807 YSB3219 YSB1901 YSB2136
STM score
6 4 2 0 -2 -4 -6 -8 -10 -12 -14
ste50 hxl1 gsk3 snf1 urk1 irk2 irk3 mpk1 ypk101 cdc2801 pho85 tco6 ire1 irk6 snf101 dak202a sck9 kic102 rad53 tco5 lcb5 iks1 tda10 rik1 rbk1 pka2 sks1 sps1 thi6 ste7 cky1 dak102 ksp1 thi20 cmk1 irk7
YSB3741 YSB3827 YSB2039 YSB2373 YSB1267 YSB1905 YSB1487 YSB3816 YSB1886 YSB3699 YSB3703 YSB2554 YSB554 YSB3831 YSB1576 YSB2489 YSB620 YSB3212 YSB3786 YSB287 YSB3790 YSB2119 YSB3223 YSB1580 YSB1511 YSB195 YSB1411 YSB3325 YSB1469 YSB343 YSB1246 YSB1941 YSB1808 YSB3220 YSB1902 YSB2137

Continued

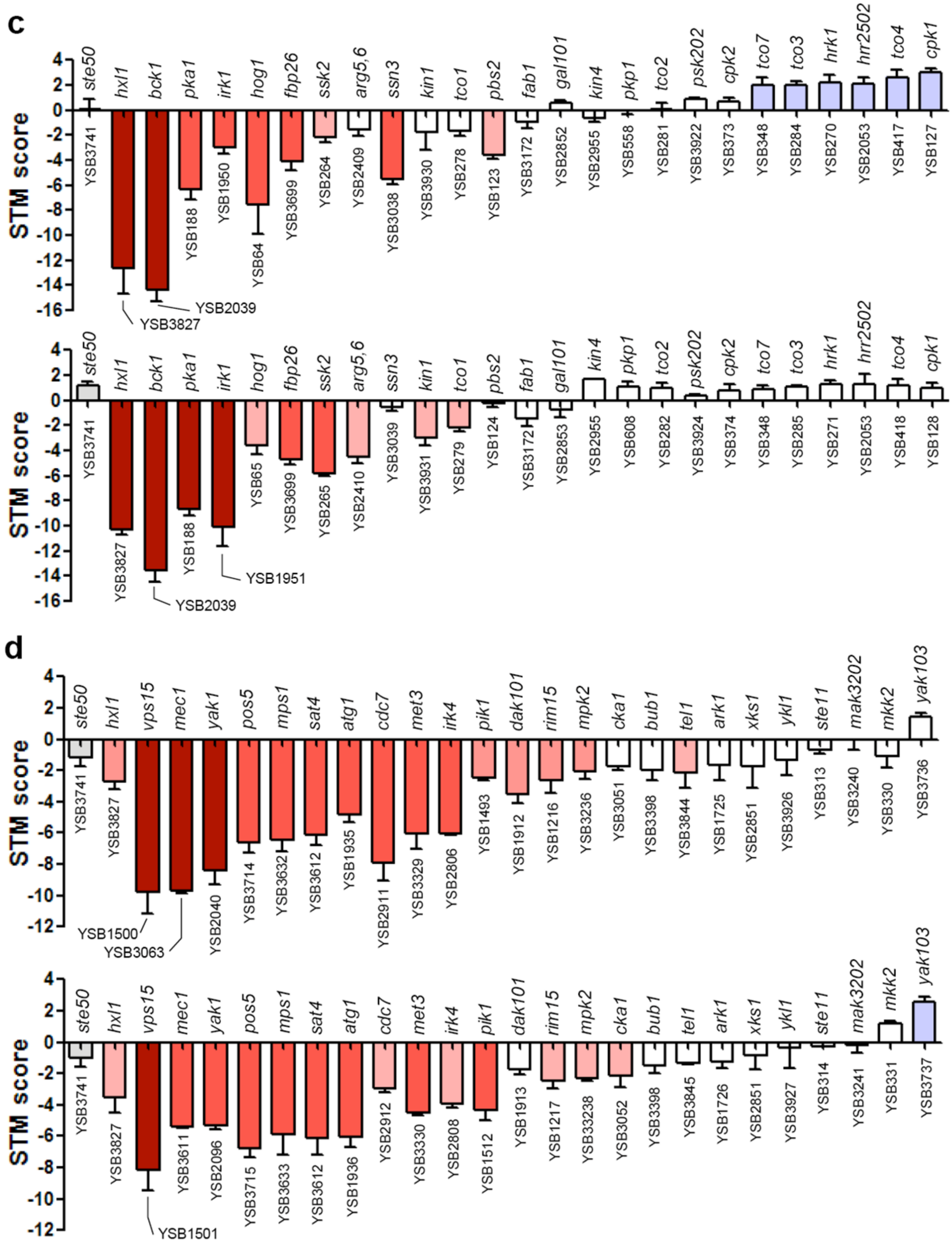
c
d
STM score
ste50
hxl1
bck1
pka1
irk1
hog1
fbp26
ssk2
arg5,6
ssn3
kin1
tco1
pbs2
fab1
gal101
kin4
pkp1
tco2
psk202
cpk2
tco7
tco3
hrk1
hrr2502
tco4
cpk1
vps15
mec1
yak1
pos5
mps1
sat4
atg1
cdc7
met3
irk4
pik1
dak101
rim15
mpk2
cka1
bub1
tel1
ark1
xks1
ykl1
ste11
mak3202
mkk2
yak103

Continued

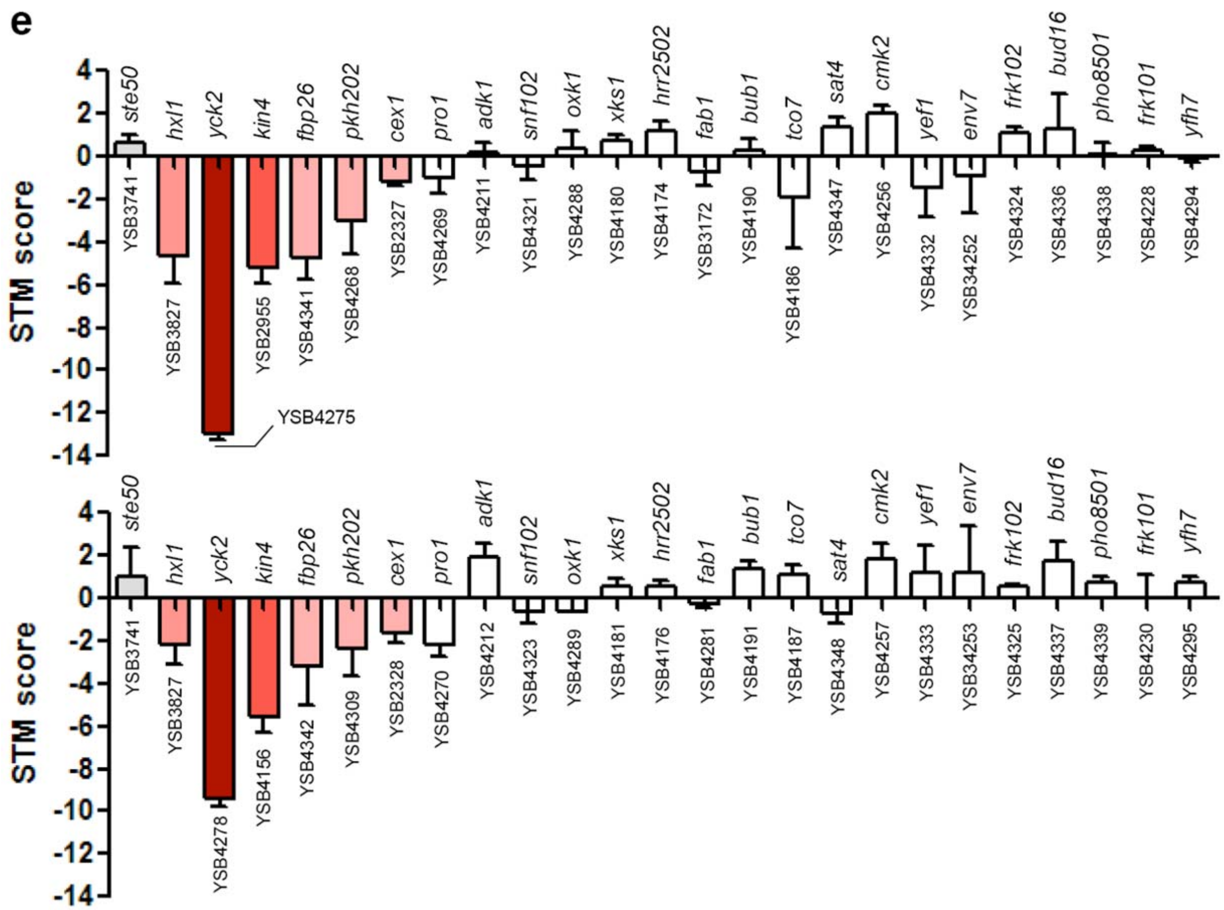

Supplementary Figure 3. Whole data for signature-tag mutagenesis (STM)-based murine model infectivity test of the *Cryptococcus neoformans* kinase mutants. STM scores were measured by using quantitative PCR analysis using a common primer and the signature tag-specific primers listed in Supplementary Data 3 for three-independent biological replicates. (**a**-**e**) All the kinase mutants were divided into five sets. Each set consisted of two-independent kinase mutant groups, which were independently assessed by STM scoring. The *y*-axis indicates the STM score. The *ste50*Δ and *hxl1*Δ mutants were used as virulent and non-virulent control strains, respectively as based on previous reports[1,2]. The colored score indicates a significant difference with respect to the control.

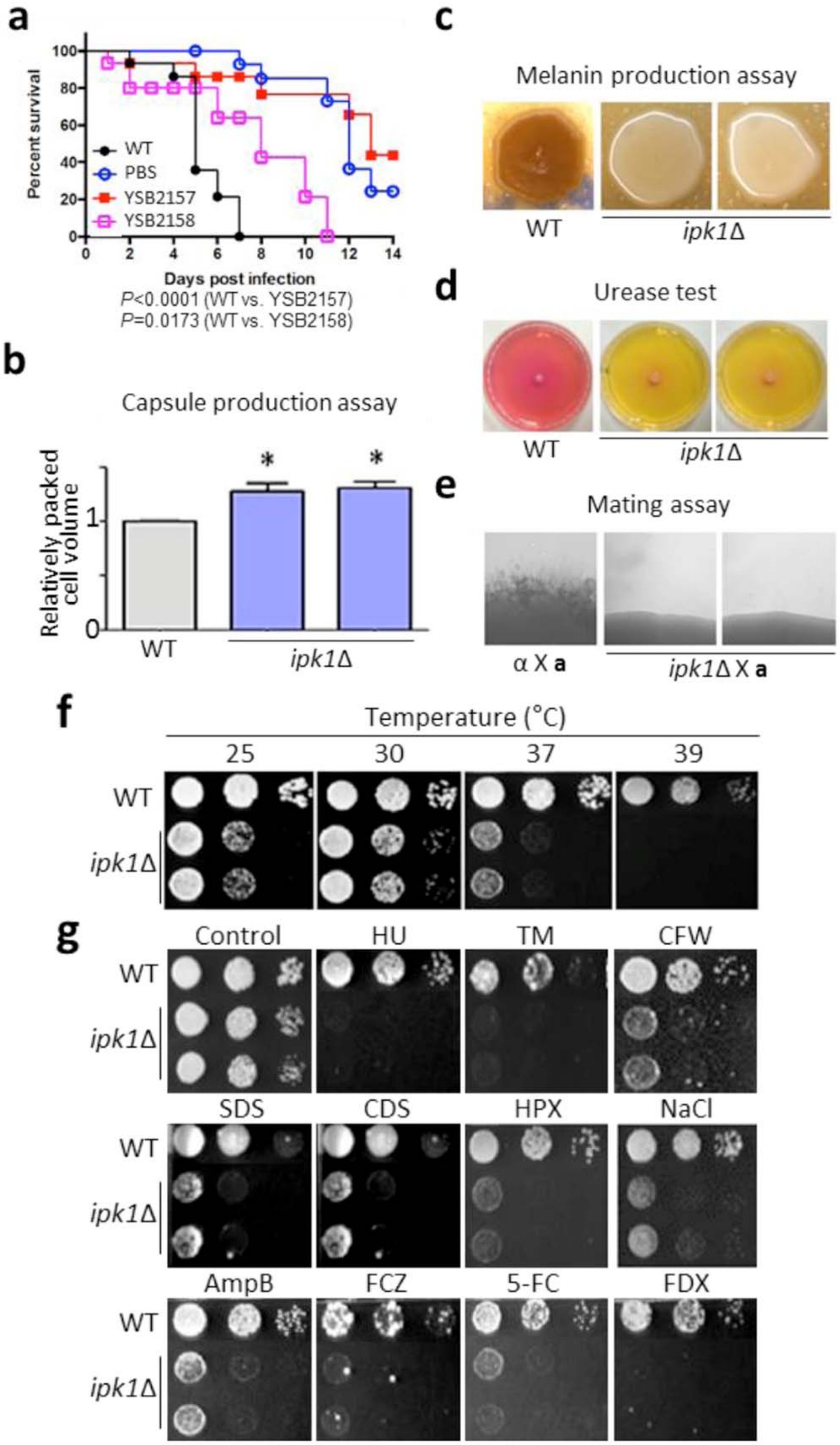
a
Percent survival
100
80
60
40
20
0
WT
PBS
YSB2157
YSB2158
0 2 4 6 8 10 12 14
Days post infection
P<0.0001 (WT vs. YSB2157)
P=0.0173 (WT vs. YSB2158)
b
Capsule production assay
Relatively packed cell volume
*
*
1
0
WT
ipk1Δ
c
Melanin production assay
WT
ipk1Δ
d
Urease test
WT
ipk1Δ
e
Mating assay
α X a
ipk1Δ X a
f
Temperature (°C)
25
30
37
39
WT
ipk1Δ
g
Control
HU
TM
CFW
WT
ipk1Δ
SDS
CDS
HPX
NaCl
WT
ipk1Δ
AmpB
FCZ
5-FC
FDX
WT
ipk1Δ

Supplementary Figure 4. Pleiotropic roles of Ipk1 in *Cryptococcus neoformans.* (**a**) *ipk1*Δ mutants (YSB2157 and YSB2158) show attenuated virulence in the insect-based *in vivo* virulence assay. WT (H99S) and PBS were used as positive and negative virulence controls. We used 15 *Galleria mellonella* caterpillars per group. *P*-values shown in the graph were calculated using the Log-rank (Mantel-Cox) test to measure statistical differences between the WT strain and *ipk1*Δ mutants strain. Survival curves were illustrated using the Prism 6 software (GraphPad). (**b**) *ipk1*Δ mutants show increased capsule production. Their relative packed cell volume ratio was calculated from three biological replicates with three technical replicates with normalization to that of the WT strain. Asterisks indicate $P < 0.05$, which is calculated by Bonferroni's multiple comparison test. Error bars indicate standard error of the mean. (**c**) *ipk1*Δ mutants show melanin-deficient phenotypes. Melanin production of WT and *ipk1*Δ strains was assayed on Niger seed plates containing 0.2 % glucose at 30°C and was photographed after 3 days. (**d**) *ipk1*Δ mutants show defects in urease production. Urease production was assayed on Christensen's agar media at 30°C and was photographed after 2 days. (**e**) *ipk1*Δ mutants display severe defects in mating. Mating was assayed on V8 media plate at room temperature in dark for 9 days and photographed. (**f, g**) WT strain and *ipk1*Δ mutants grown overnight were 10-fold serially diluted (10^2 to 10^4-fold dilution) and spotted on solid YPD medium (**f**) or YPD medium containing the following chemicals (**g**): HU, 100 mM hydroxyurea; TM, 0.3 μg/ml tunicamycin; CFW, 3 mg ml^{-1} of calcofluor white; SDS, 0.03 % sodium dodecyl sulphate; CDS, 30 μM $CdSO_4$; HPX, 3 mM of H_2O_2; 1M NaCl; AmpB, 0.9 μg ml^{-1} amphotericin B; FCZ, 14 μg ml^{-1} fluconazole; 5-FC, 300 μg ml^{-1} flucytosine; FDX, 1 μg ml^{-1} fludioxonil. Cells were incubated at 30°C (**g**) or the indicated temperature (**f**) for 3 days and photographed.

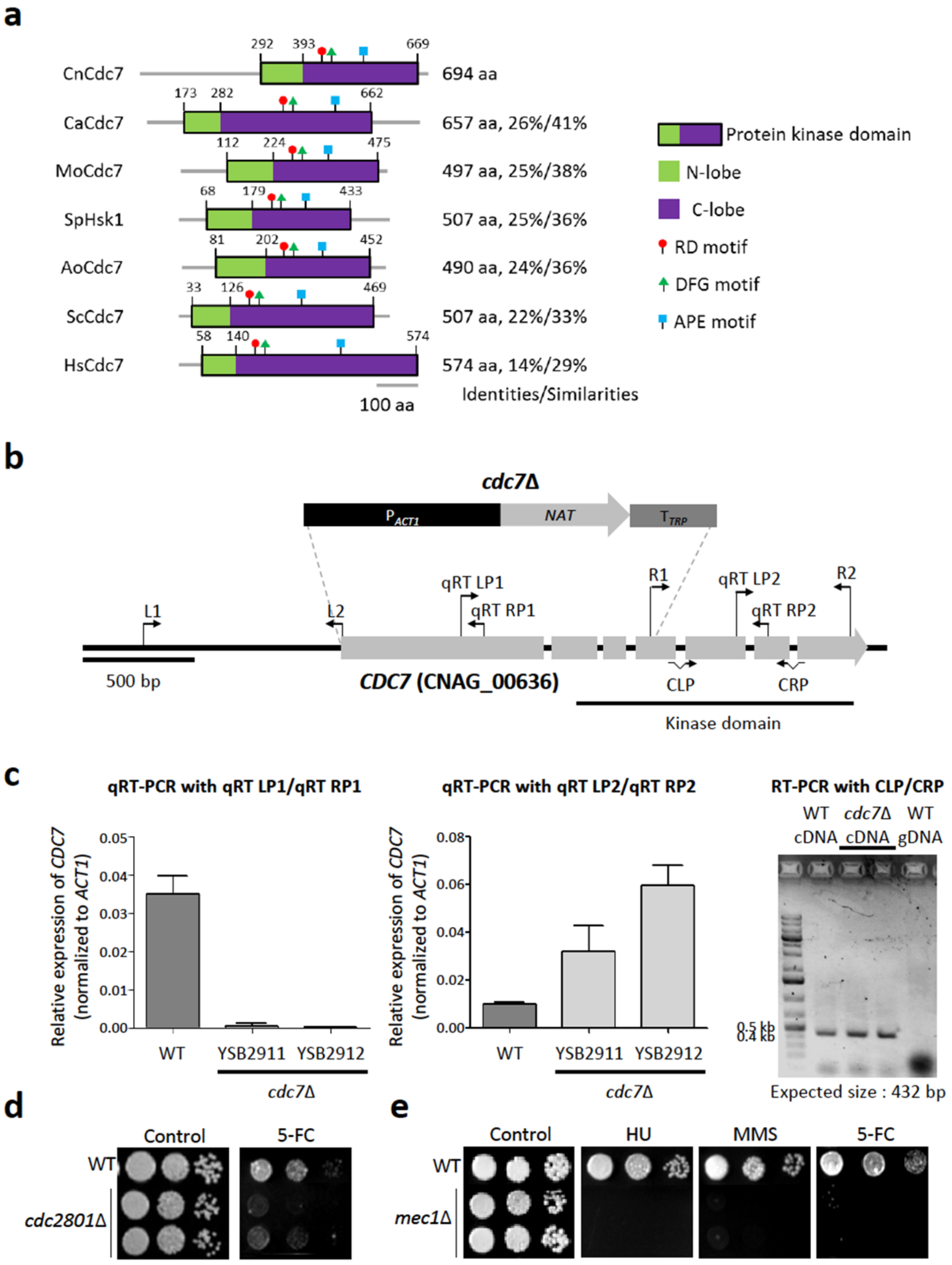
a
CnCdc7
694 aa
CaCdc7
657 aa, 26%/41%
MoCdc7
497 aa, 25%/38%
SpHsk1
507 aa, 25%/36%
AoCdc7
490 aa, 24%/36%
ScCdc7
507 aa, 22%/33%
HsCdc7
574 aa, 14%/29%
100 aa
Identities/Similarities
Protein kinase domain
N-lobe
C-lobe
RD motif
DFG motif
APE motif
b
cdc7Δ
NAT
L1
L2
qRT LP1
qRT RP1
R1
qRT LP2
qRT RP2
R2
500 bp
CDC7 (CNAG_00636)
CLP
CRP
Kinase domain
c
qRT-PCR with qRT LP1/qRT RP1
Relative expression of CDC7 (normalized to ACT1)
WT
YSB2911
YSB2912
cdc7Δ
qRT-PCR with qRT LP2/qRT RP2
RT-PCR with CLP/CRP
WT cDNA
cdc7Δ cDNA
WT gDNA
0.5 kb
0.4 kb
Expected size : 432 bp
d
Control
5-FC
WT
cdc2801Δ
e
Control
HU
MMS
5-FC
WT
mec1Δ

Supplementary Figure 5. Construction of the partial *CDC7* deletion mutants and characterisation of the role of Cdc2801 and Mec1 in genotoxic stress response in *Cryptococcus neoformans*. (**a**) Protein structure of fungal and human Cdc7 orthologues. Cn, *Cryptococcus neoformans*, CNAG_00636; Ca, *Candida albicans*, AAW28083; Mo, *Magnaporthe oryzae*, XP_003716435; Sp, *Schizosaccharomyces pombe*, NP_596328; Ao, *Aspergillus oryzae*, EIT77902; Sc, *Saccharomyces cerevisiae*, NP_010267; Hs, *Homo sapiens*, NP_003494. The protein kinase domains were predicted by UniProt (http://uniprot.org). The protein kinase domain consists of N-and C-lobes. It is known that an active site is located in a cleft between N- and C-lobes[3]. The C-lobe contains the catalytic loop having the conserved RD (ArgAsp), DFG (AspPheGly), and APE (AlaProGlu) motifs. The regions of N- and C-lobes in fungal Cdc7 orthologues were identified by aligning them with the human Cdc7 whose crystal structure was previously reported[3]. Amino acid sequence identities and similarities of fungal Cdc7 orthologues to the *C. neoformans* Cdc7 were indicated at the right side of the protein structure. (**b**) The scheme for primers used in *cdc7*Δ mutant production and confirmation of *cdc7*Δ mutants. The grey boxes indicate the exons of *CDC7*. The locations of a kinase domain on the *CDC7* gene are presented below the scheme. The primer pairs L1/L2 and R1/R2 were used for constructing the *cdc7*Δ mutants. (**c**) The remaining region of *CDC7* in the *cdc7*Δ mutant is expressed as mRNA. For the qRT-PCR and PCR to confirm *CDC7* expression in the *cdc7*Δ mutants, RNA was isolated from the WT strain (H99S) and the *cdc7*Δ mutants (YSB2911 and YSB2912). Quantitative RT-PCR (qRT-PCR) using qRT LP1/qRT RP1 and qRT LP2/qRT RP2 primer pairs was performed with three technical replicates. Error bars indicate standard deviation. PCR to confirm the expression of the remaining region of *CDC7* was performed using the CLP/CRP primer pair. The PCR products were run on the 1% agarose gel. The PCR result of the genomic DNA from the WT strain was used as the negative control. (**d-e**) *cdc2801*Δ mutants (YSB2370 and YSB3699) and *mec1*Δ mutants (YSB3063 and YSB3611) were spotted on YPD medium or on YPD medium containing the following chemical agents: (**d**) 300 μg ml^{-1} flucytosine (5-FC), (**e**) 100 mM hydroxyurea (HU), 0.06 % methyl methanesulphonate (MMS), 300 μg ml^{-1} flucytosine (5-FC), followed by further incubation at 30°C, and were photographed after 3 days.

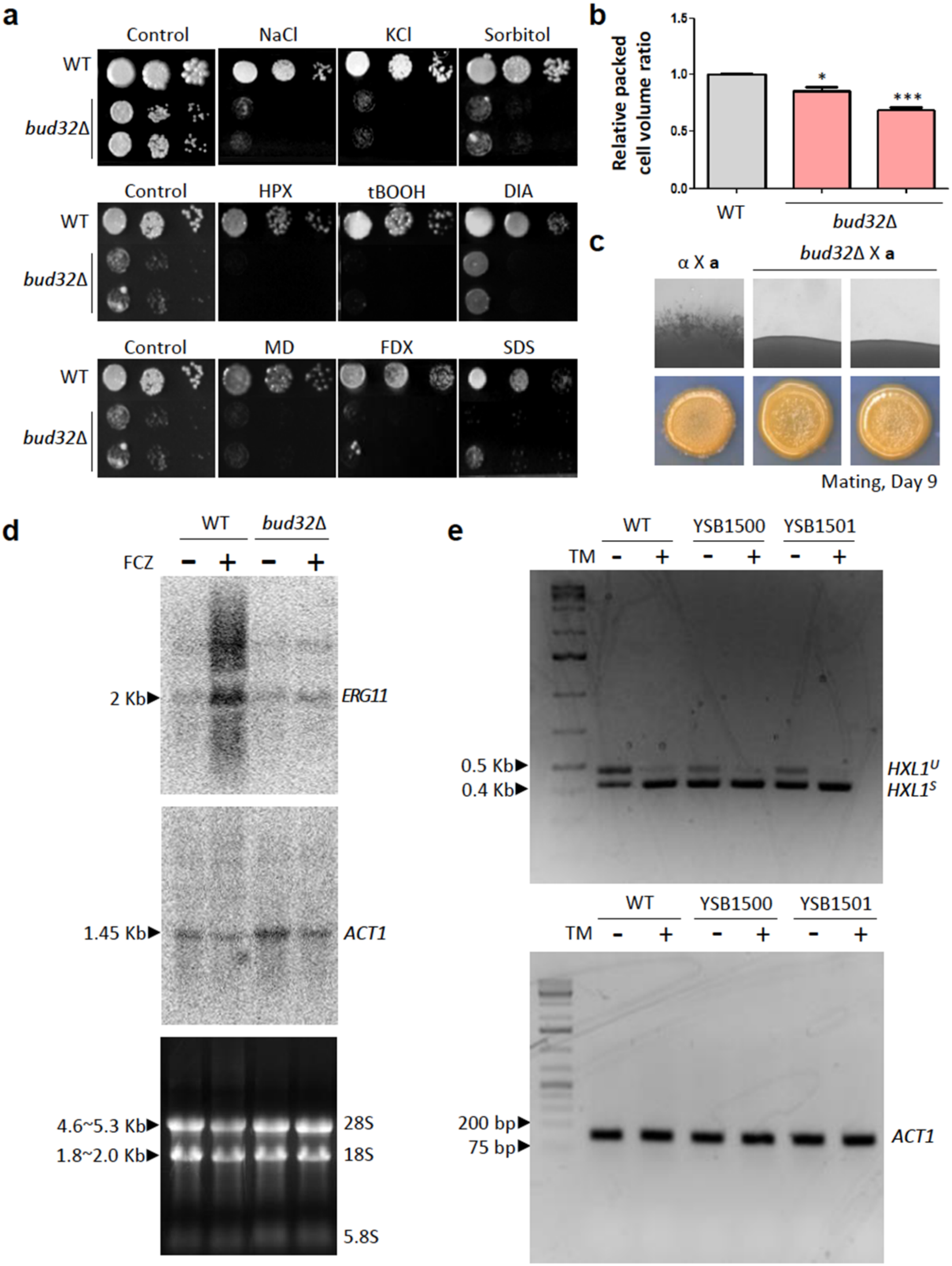

a
Control
NaCl
KCl
Sorbitol
WT
bud32Δ
Control
HPX
tBOOH
DIA
Control
MD
FDX
SDS
b
Relative packed cell volume ratio
1.5
1.0
0.5
0.0
*
***
WT
bud32Δ
c
α X a
bud32Δ X a
Mating, Day 9
d
WT
bud32Δ
FCZ
2 Kb
ERG11
1.45 Kb
ACT1
4.6~5.3 Kb
1.8~2.0 Kb
28S
18S
5.8S
e
WT
YSB1500
YSB1501
TM
0.5 Kb
0.4 Kb
HXL1U
HXL1S
200 bp
75 bp
ACT1

Supplementary Figure 6. Pleiotropic roles of Bud32 and the impact of *VPS15* deletion in activation of the UPR pathway in *Cryptococcus neoformans.* (**a**) WT strain (H99S) and *bud32*Δ mutants (YSB1968 and YSB1969) were cultured for 16 hr at 30°C in liquid YPD medium, 10-fold serially diluted (undiluted to 10^4) and spotted on solid YPD medium containing the following chemicals: 1.5 M NaCl, 1.5 M KCl, 2 M Sorbitol, 3 mM hydrogen peroxide (HPX), 0.7 mM *tert*-butyl hydroperoxide (tBOOH), 2 mM diamide (DIA), 0.02 mM menadione (MD), 1 μg ml^{-1} fludioxonil (FDX) and 0.03% sodium dodecyl sulphate (SDS). The cells were further incubated at 30°C and photographed. (**b**) To monitor the capsule production, WT and *bud32*Δ strains spotted onto DME plate were incubated for 2 days at 37°C, scraped and then injected into capillary tubes. After cells were packed by gravity, the packed cell volume ratio was calculated as described in Materials and Methods and the relative packed cell volume ratio (*y*-axis) was obtained by normalization in comparison to WT value. Three-independent experiments were performed to consider statistically significant by one-way ANOVA test in Bonferroni correction (*; 0.0205, ***; 0.0003, s.e.m.). (**c**) To examine the mating efficacy, WT and *bud32*Δ strains were spotted onto V8 mating medium and incubated at room temperature in the dark for 9 days. (**d**) WT and *bud32*Δ (YSB1968) strains grown at 30°C to the logarithmic phase were treated with (+) or without (-) 10 μ ml^{-1} fluconazole for 90 min, and total RNA was extracted. The total RNA was separated by gel-electrophoresis for northern blot analysis. *ERG11* and *ACT1* probes were amplified using each primer set listed in Supplementary Data 8. These full northern blot and gel pictures were cropped to indicate bands of *ERG11* and *ACT1* transcripts of expected size and quantified as shown in Fig. 6n. (**e**) For RT-PCR, total RNA was extracted from WT strain and *vps15*Δ mutants (YSB1500 and YSB1501) and cDNA was synthesized. Specific primer sets for RT-PCR were listed in Supplementary Data 8. The full gel pictures were cropped to indicate the bands of spliced and unspliced *HXL1* transcripts ($HXL1^s$ and $HXL1^u$, respectively) and presented in Fig. 7f.

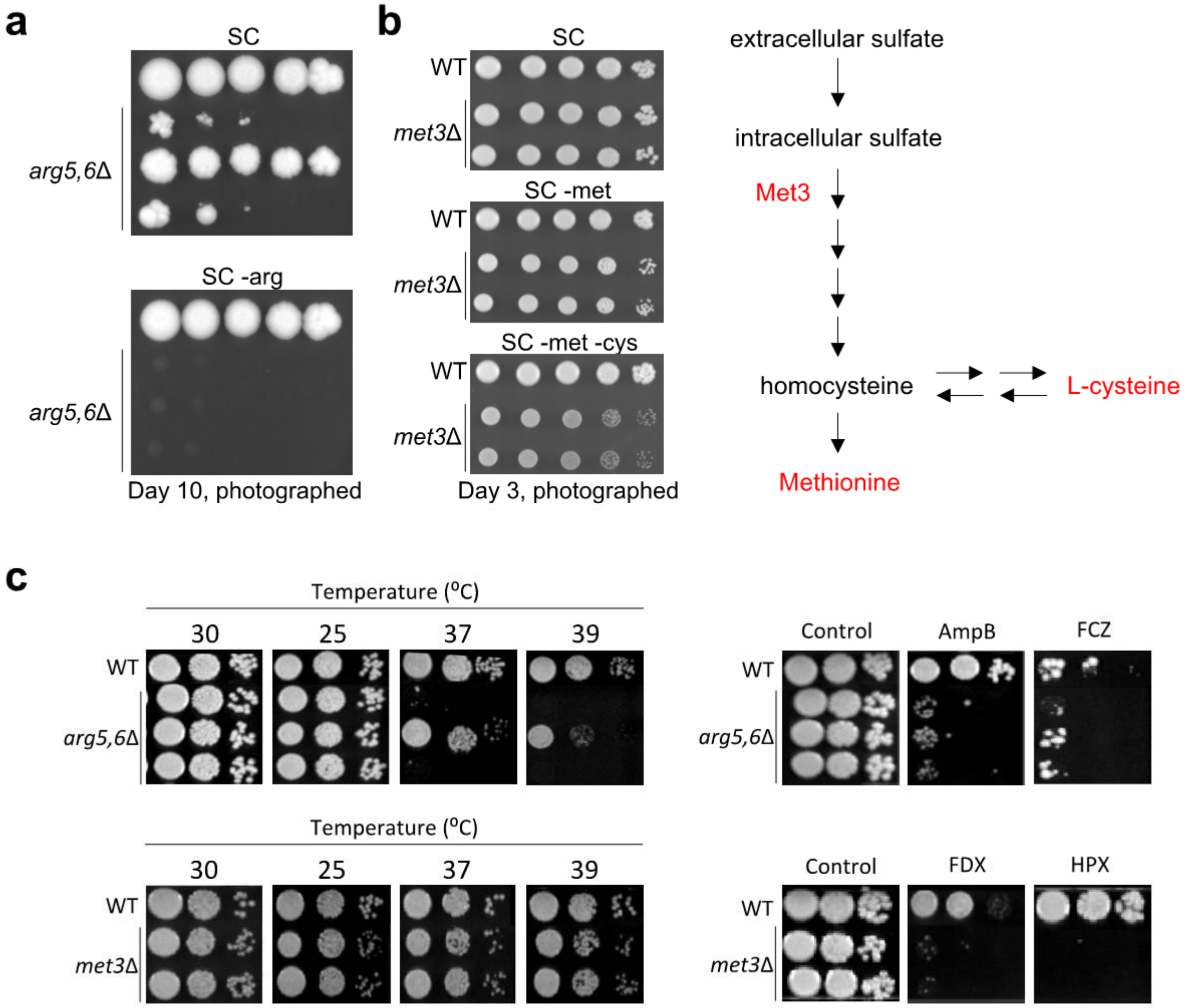

Supplementary Figure 7. Phenotypic traits of strains deleted of pathogenicity-regulating kinase genes involved in nutrient metabolism of *Cryptococcus neoformans*. (**a**,**b**) Wild-type strain (H99S) and *arg5,6*Δ (YSB2408, YSB2409, and YSB2410) and *met3*Δ (YSB3329 and YSB3330) mutants were cultured for 16 hr in liquid YPD medium, washed twice with PBS, 10-fold serially diluted (undiluted to 10^4) and spotted on solid synthetic complete medium [SC; yeast nitrogen base without amino acids (Difco) supplemented with the indicated concentration of the following amino acids and nucleotides: 30 mg l^{-1} L-isoleucine, 0.15 g l^{-1} L-valine, 20 mg l^{-1} adenine sulphate, 20 mg l^{-1} L-histidine-HCl, 0.1 g l^{-1} L-leucine, 30 mg l^{-1} L-lysine, 50 mg l^{-1} L-phenylalanine, 20 mg l^{-1} L-tryptophan, 30 mg l^{-1} uracil, 0.4 g l^{-1} L-serine, 0.1 g l^{-1} glutamic acid, 0.2 g l^{-1} L-threonine, 0.1 g l^{-1} L-aspartate, 20 mg l^{-1} L-arginine, 20 mg l^{-1} L-cysteine, and 20 mg l^{-1} L-methionine]. SC-arg (**a**), SC-met and SC-met-cys (**b**) media indicate the SC medium lacking arginine, methionine and/or cysteine supplements. The right panel in (**b**) describes the simplified biosynthetic pathway for methionine and cysteine. (**c**) WT strain (H99S) and *arg5,6*Δ and *met3*Δ mutants were cultured for 16 hr at 30°C in liquid YPD medium, 10-fold serially diluted (undiluted to 10^4), and spotted on YPD medium with or without the indicated concentration of the following chemicals: 1 μg ml^{-1} amphotericin B (AmpB), 14 μg ml^{-1} fluconazole (FCZ), 1 μg ml^{-1} fludioxonil (FDX), 3 mM hydrogen peroxide (HPX). Cells were further incubated at 30°C (control) or indicated temperature for 3 days and photographed.

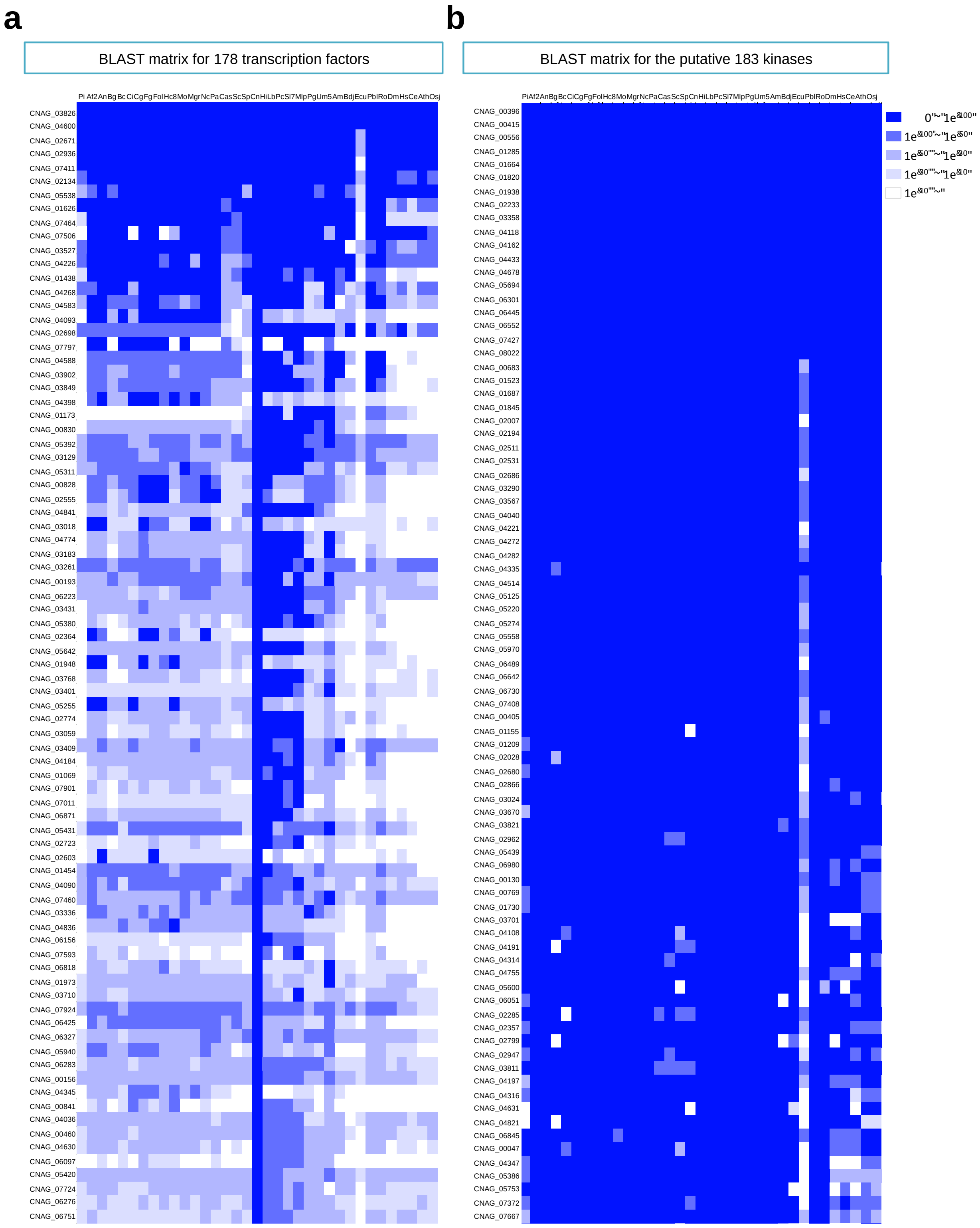
a
BLAST matrix for 178 transcription factors
Pi Af2 An Bg Bc Ci Cg Fg Fol Hc8 Mo Mgr Nc Pa Cas Sc Sp Cn Hi Lb Pc Sl7 Mlp Pg Um5 Am Bdj Ecu Pbl Ro Dm Hs Ce Ath Osj
b
BLAST matrix for the putative 183 kinases
Pi Af2 An Bg Bc Ci Cg Fg Fol Hc8 Mo Mgr Nc Pa Cas Sc Sp Cn Hi Lb Pc Sl7 Mlp Pg Um5 Am Bdj Ecu Pbl Ro Dm Hs Ce Ath Osj

Continued

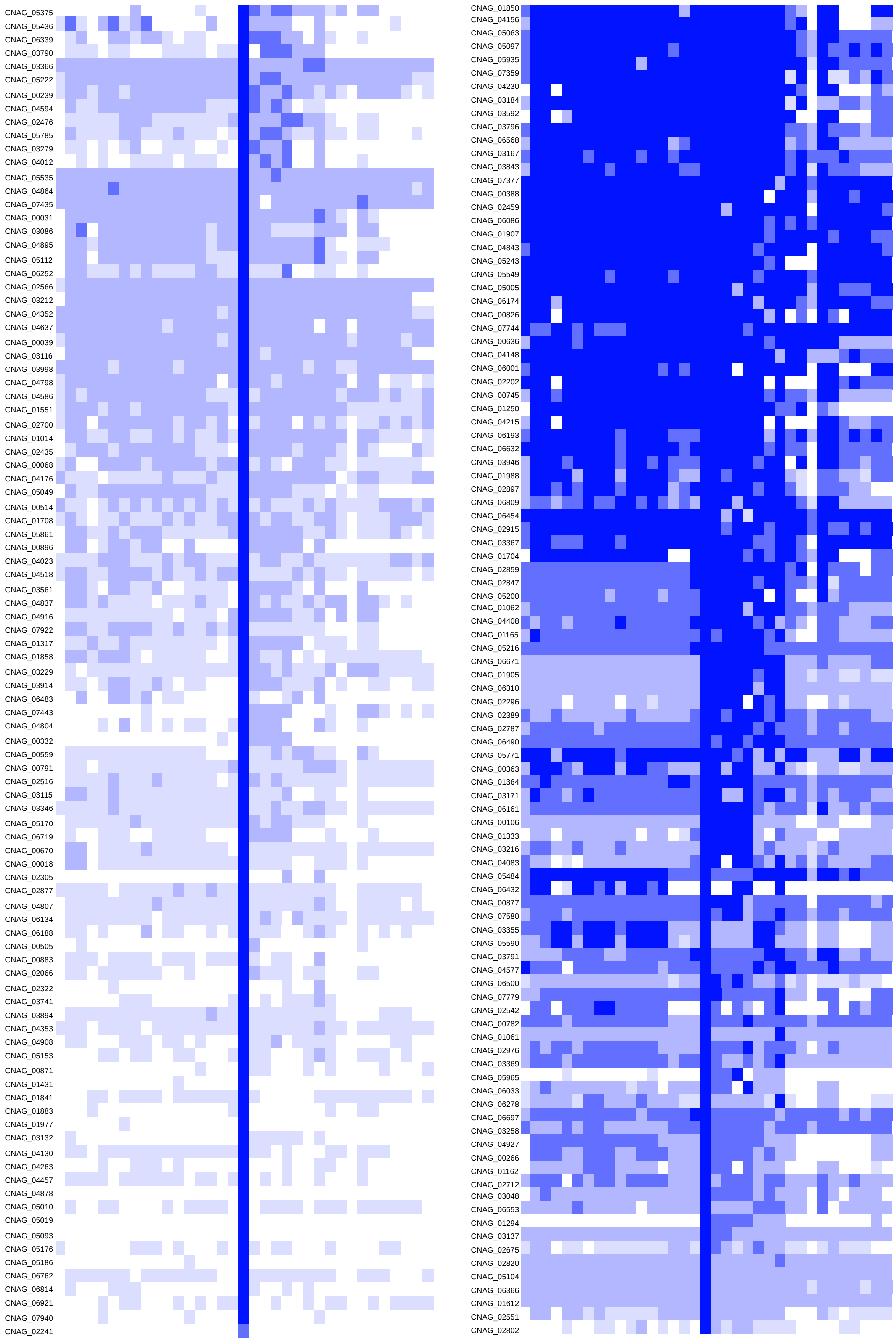
CNAG_05375
CNAG_05436
CNAG_06339
CNAG_03790
CNAG_03366
CNAG_05222
CNAG_00239
CNAG_04594
CNAG_02476
CNAG_05785
CNAG_03279
CNAG_04012
CNAG_05535
CNAG_04864
CNAG_07435
CNAG_00031
CNAG_03086
CNAG_04895
CNAG_05112
CNAG_06252
CNAG_02566
CNAG_03212
CNAG_04352
CNAG_04637
CNAG_00039
CNAG_03116
CNAG_03998
CNAG_04798
CNAG_04586
CNAG_01551
CNAG_02700
CNAG_01014
CNAG_02435
CNAG_00068
CNAG_04176
CNAG_05049
CNAG_00514
CNAG_01708
CNAG_05861
CNAG_00896
CNAG_04023
CNAG_04518
CNAG_03561
CNAG_04837
CNAG_04916
CNAG_07922
CNAG_01317
CNAG_01858
CNAG_03229
CNAG_03914
CNAG_06483
CNAG_07443
CNAG_04804
CNAG_00332
CNAG_00559
CNAG_00791
CNAG_02516
CNAG_03115
CNAG_03346
CNAG_05170
CNAG_06719
CNAG_00670
CNAG_00018
CNAG_02305
CNAG_02877
CNAG_04807
CNAG_06134
CNAG_06188
CNAG_00505
CNAG_00883
CNAG_02066
CNAG_02322
CNAG_03741
CNAG_03894
CNAG_04353
CNAG_04908
CNAG_05153
CNAG_00871
CNAG_01431
CNAG_01841
CNAG_01883
CNAG_01977
CNAG_03132
CNAG_04130
CNAG_04263
CNAG_04457
CNAG_04878
CNAG_05010
CNAG_05019
CNAG_05093
CNAG_05176
CNAG_05186
CNAG_06762
CNAG_06814
CNAG_06921
CNAG_07940
CNAG_02241
CNAG_01850
CNAG_04156
CNAG_05063
CNAG_05097
CNAG_05935
CNAG_07359
CNAG_04230
CNAG_03184
CNAG_03592
CNAG_03796
CNAG_06568
CNAG_03167
CNAG_03843
CNAG_07377
CNAG_00388
CNAG_02459
CNAG_06086
CNAG_01907
CNAG_04843
CNAG_05243
CNAG_05549
CNAG_05005
CNAG_06174
CNAG_00826
CNAG_07744
CNAG_00636
CNAG_04148
CNAG_06001
CNAG_02202
CNAG_00745
CNAG_01250
CNAG_04215
CNAG_06193
CNAG_06632
CNAG_03946
CNAG_01988
CNAG_02897
CNAG_06809
CNAG_06454
CNAG_02915
CNAG_03367
CNAG_01704
CNAG_02859
CNAG_02847
CNAG_05200
CNAG_01062
CNAG_04408
CNAG_01165
CNAG_05216
CNAG_06671
CNAG_01905
CNAG_06310
CNAG_02296
CNAG_02389
CNAG_02787
CNAG_06490
CNAG_05771
CNAG_00363
CNAG_01364
CNAG_03171
CNAG_06161
CNAG_00106
CNAG_01333
CNAG_03216
CNAG_04083
CNAG_05484
CNAG_06432
CNAG_00877
CNAG_07580
CNAG_03355
CNAG_05590
CNAG_03791
CNAG_04577
CNAG_06500
CNAG_07779
CNAG_02542
CNAG_00782
CNAG_01061
CNAG_02976
CNAG_03369
CNAG_05965
CNAG_06033
CNAG_06278
CNAG_06697
CNAG_03258
CNAG_04927
CNAG_00266
CNAG_01162
CNAG_02712
CNAG_03048
CNAG_06553
CNAG_01294
CNAG_03137
CNAG_02675
CNAG_02820
CNAG_05104
CNAG_06366
CNAG_01612
CNAG_02551
CNAG_02802

Supplementary Figure 8. BLAST matrix analysis of putative transcription factors and kinases in *Cryptococcus neoformans*. BLAST matrix analysis was performed using the Comparative Fungal Genomics Platform (CFGP, http://cfgp.riceblast.snu.ac.kr) database[4]. (**a**) BLAST matrix of 178 putative transcription factor genes in the *C. neoformans* genome against corresponding orthologs in 35 eukaryotic species. (**b**) BLAST matrix of the 183 putative kinase genes in the *C. neoformans* genome (Supplementary Data 1) against corresponding orthologs in 35 eukaryotic species. Each abbreviation for the 35 eukaryotic species indicates the following: (Pi; *Phytophthora infestans*, Af; *Aspergillus fumigatus*, An; *Aspergillus nidulans*, Bg; *Blumeria graminis*, Bc; *Botrytis cinerea*, Ci; *Coccidioides immitis*, Cg; *Colletotrichum graminicola*, Fg; *Fusarium graminearum*, Fo; *Fusarium oxysporum*, Hc; *Histoplasma capsulatum*, Mo; *Magnaporthe oryzae*, Mg; *Mycosphaerella graminicola*, Nc; *Neurospora crassa*, Pa; *Podospora anserina*, Ca; *Candida albicans*, Sc; *Saccharomyces cerevisiae*, Sp; *Schizosaccharomyces pombe*, Cn; *Cryptococcus neoformans*, Hi; Heterobasidion irregulare, Lb; *Laccaria bicolor*, Pc; *Phanerochaete chrysosporium*, Sl; *Serpula lacrymans*, Ml; *Melampsora laricis-populina*, Pg; *Puccinia graminis*, Um; *Ustilago maydis*, Am; *Allomyces macrogynus*, Bd; *Batrachochytrium dendrobatidis*, Ec; *Encephalitozoon cuniculi*, Pb; *Phycomyces blakesleeanus,* Ro; *Rhizopus oryzae,* Dm; *Drosophila melanogaster*, Hs; *Homo sapiens*, Ce; *Caenorhabditis elegans,* At; *Arabidopsis* thaliana, Os; *Oryza sativa)*

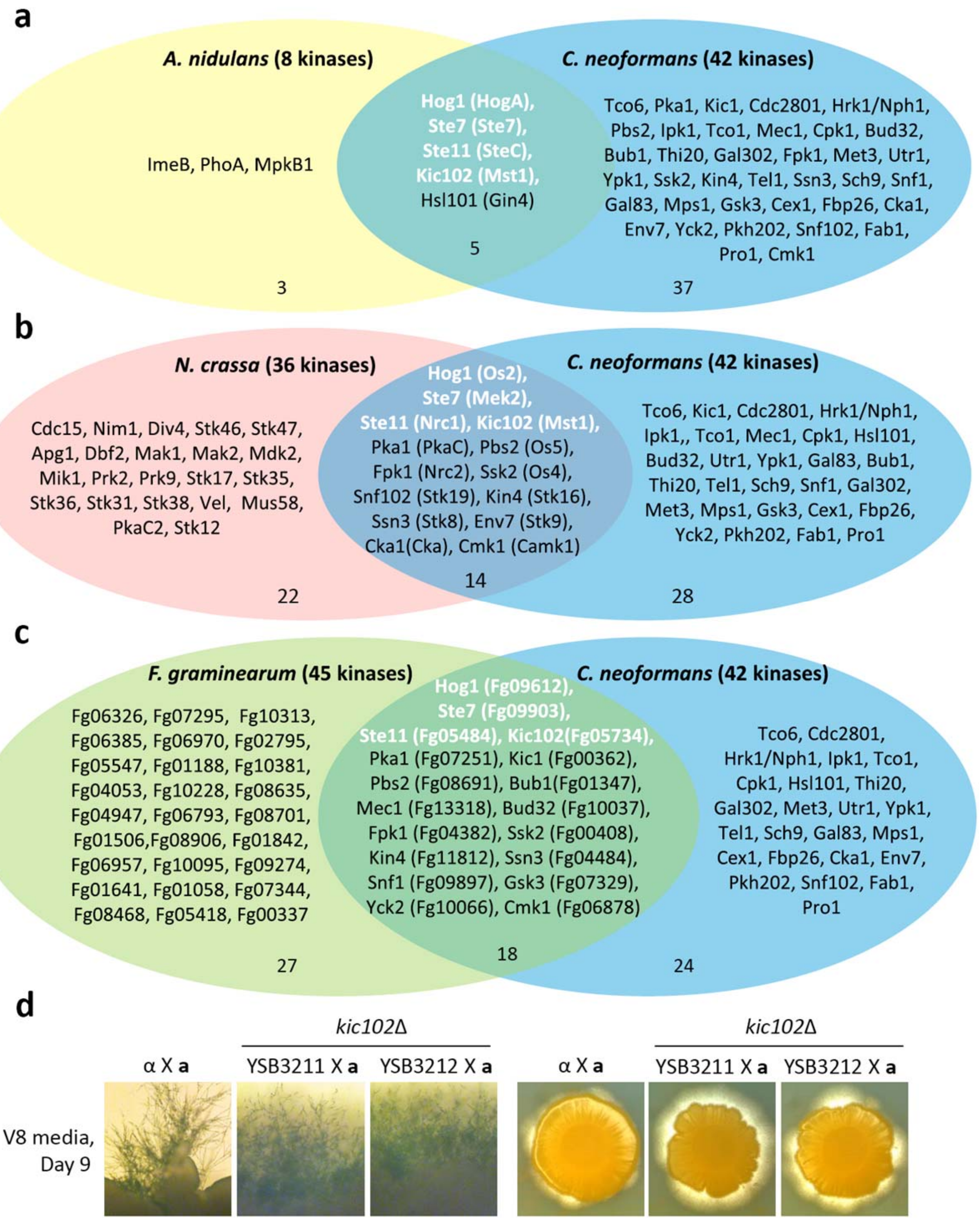

Supplementary Figure 9. Kinases regulating sexual development in fungi. (**a-c**) Venn diagram showing the distribution of kinases involved in the developmental process of the human fungal pathogen *Cryptococcus neoformans*, non-pathogenic model filamentous fungi (*Aspergillus nidulans* and *Neurospora crassa*) and a plant pathogenic filamentous fungus (*Fusarium graminearum*). (**d**) Filamentous growth of *KIC102* mutants and the wild-type strain during mating of *C. neoformans* was observed by light microscopy.

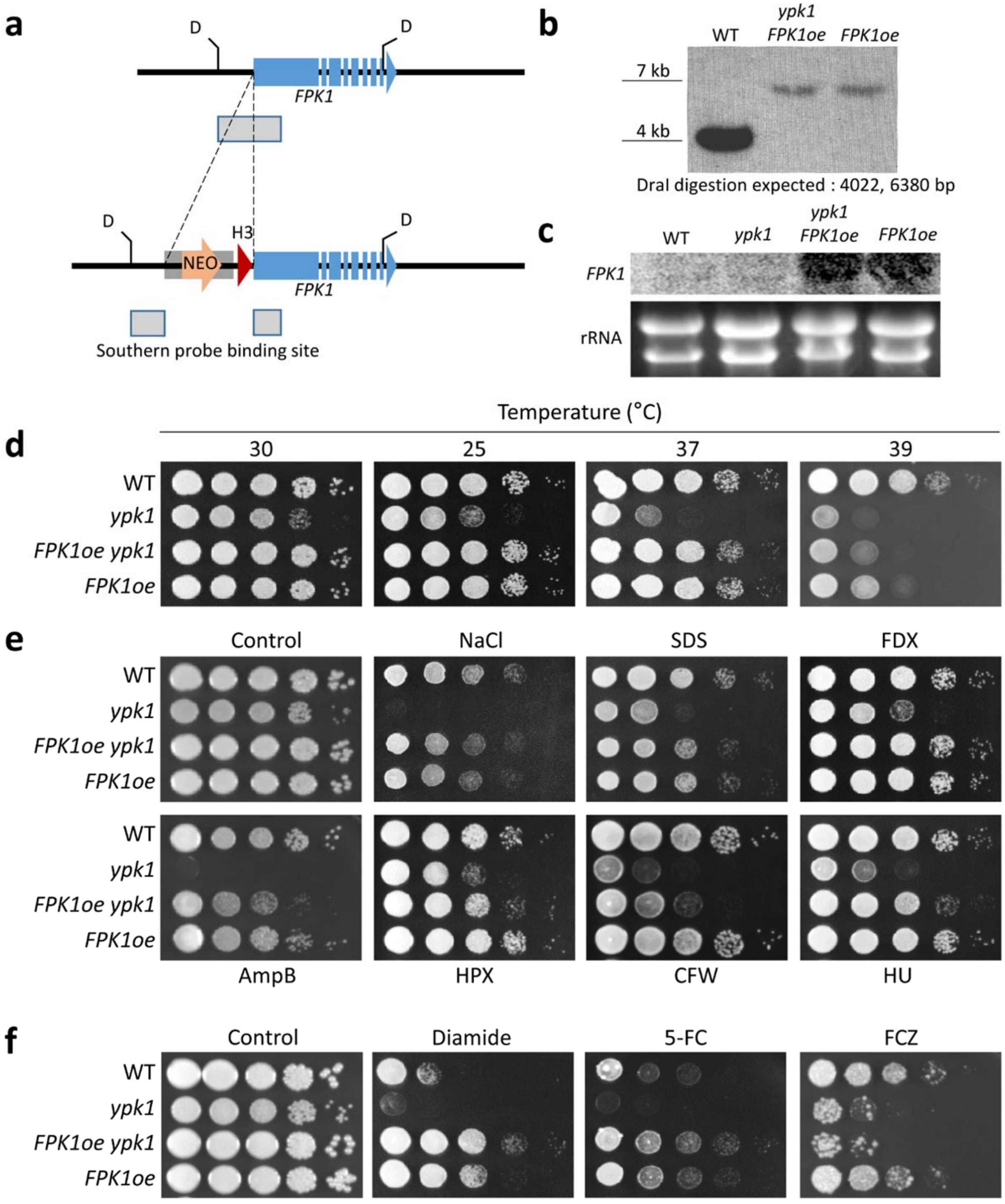
a
D
D
FPK1
D
NEO
H3
D
FPK1
Southern probe binding site
b
WT
ypk1 FPK1oe
FPK1oe
7 kb
4 kb
DraI digestion expected : 4022, 6380 bp
c
WT
ypk1
ypk1 FPK1oe
FPK1oe
FPK1
rRNA
Temperature (°C)
d
30
25
37
39
WT
ypk1
FPK1oe ypk1
FPK1oe
e
Control
NaCl
SDS
FDX
AmpB
HPX
CFW
HU
f
Control
Diamide
5-FC
FCZ

Continued

g

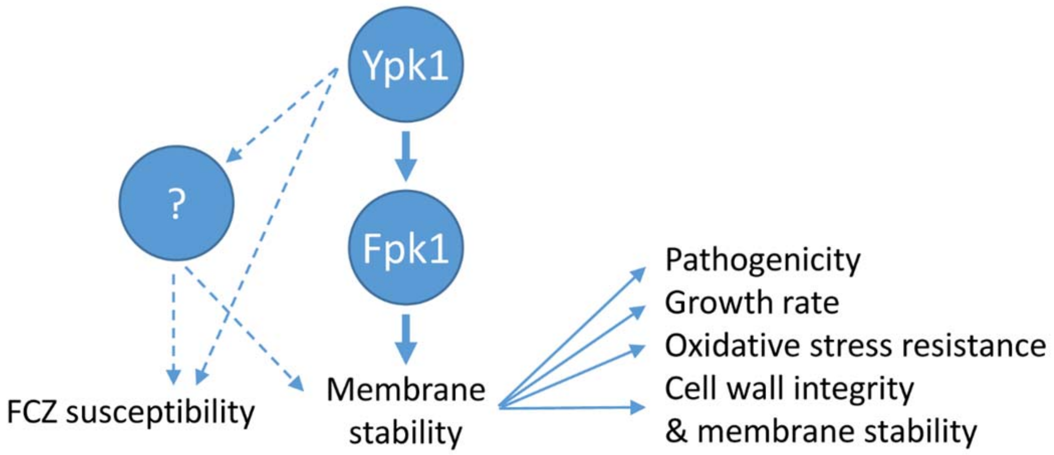

Supplementary Figure 10. Fpk1 regulates a subset of Ypk1-dependent phenotypes in the pathogenicity of *Cryptococcus neoformans*. (**a**) A scheme for the replacement of the *FPK1* promoter with histone H3 promoter for constitutive *FPK1* overexpression. (**b**) Genotyping of *FPK1* overexpression strains by Southern blot analysis. *FPK1oe* (YSB3986) and *ypk1 FPK1oe* (YSB3981) indicate *FPK1* overexpression strains in wild-type and *ypk1*Δ mutant backgrounds, respectively. (**c**) Verification of *FPK1* overexpression by northern blot analysis. rRNA was shown as a loading control. (**d**) WT strain (H99S), *ypk1*Δ (YSB1736), *FPK1* overexpression strains (YSB3986 and YSB3981) were cultured for 16 hr, spotted on YPD medium, and incubated at the indicated temperature for 3 days. (**e**,**f**) The same set of strains described in (**d**) were spotted on YPD medium containing 1.5 M NaCl, 0.04 % sodium dodecyl sulphate (SDS), 1 μg ml^{-1} fludioxonil (FDX), 1 μg ml^{-1} amphotericin B (AmpB), 3 mM hydrogen peroxide (HPX), 3 mg ml^{-1} calcofluor white (CFW), 100 mM hydroxyurea (HU), 2 mM diamide, 300 μg ml^{-1} of flucytosine (5-FC), 5 mg ml^{-1} fluconazole (FCZ). Cells were further incubated at 30°C for 3 days and photographed. (**g**) The proposed regulatory model for Ypk1 and Fpk1 in *C. neoformans*.

Supplementary Table 1. Kinases involved in the virulence of *Cryptococcus neoformans*

Gene name	Pathogenicity		Phenotypes			
	Murine model (STM score)	Insect model (RMS score)	Capsule	Melanin	Urease	Stress response
FPK1	-17.28	0.86				Ox/Cm
BCK1	**-13.94**	**2.10***	↑	↓↓↓		Th/Os/Ox/Gx/Cw/Cm
GAL83	**-12.37**	**1.50***			↑↑↑	Os/Ox/ER/Cm
GSK3	**-12.22**	**1.71***	↑↑↑	↓↓↓	↓↓	Th/Os/Ox/Gx/ER/Hm/Cm
YCK2	**-11.18**	**1.75***			↑	Th/Os/Ox/Gx/ER/Hm/Cw/Cm
KIC1	**-11.09**	**1.46***		↓↓↓	↓↓	Th/Os/Ox/Gx/ER/Hm/Cw/Cm
YPK1	**-9.74**	**1.90***	↑↑↑	↓↓↓	↓↓↓	Th/Os/Ox/Gx/ER/Cw/Cm
VPS15	**-9.00**	**1.90***		↓↓↓		Th/Os/Ox/Gx/ER/Hm/Cw/Cm
SNF1	**-8.32**	**2.30***		↓	↑	Os/Ox/Cm
IPK1	**-7.90**	**2.00***	↑↑	↓↓↓	↓↓	Th/Os/Ox/Gx/ER/Hm/Cw/Cm
MEC1	**-7.59**	**1.92***	↓	↓↓	↓	Th/Os/Ox/Gx/Hm/Cw/Cm
PKA1	**-7.55**	**1.36***	↓↓↓	↓↓↓	↑↑	Ox/Gx/ER
URK1	**-7.51**	**1.50***				Th/Ox
YAK1	-6.86	1.67			↓↓↓	Ox/ER/Cw
POS5	**-6.71**	**1.90***	↓		↓↓↓	Os/Ox/Gx/ER/Hm/Cw/Cm
IRK1	-6.57	1.20				Ox/Hm
HSL101	-6.54	1.08	↑↑↑		↓↓	Os/Gx/Hm
IRK2	-6.40	1.04			↑	
MPS1	**-6.17**	**1.38***		↓↓		Th/Os/Ox/Gx/ER/Hm/Cw
SAT4	**-6.11**	**1.50***				Os/Ox/Gx/ER/Cm
IRK3	-6.07	1.00				
MPK1	**-5.63**	**2.40***	↓↓		↑	Th/Os/Ox/Gx/ER/Hm/Cw/Cm
HOG1	-5.60	1.17	↑↑↑	↑↑↑		Th/Os/Ox/Gx/ER/Hm/Cw/Cm
ATG1	-5.44	1.00	↓			
CDC7	**-5.43**	**2.17***			↓↓	Th/Os/Ox/Gx/Cw/Cm
KIN4	**-5.39**	**1.67***				Os/Ox/ER/Hm
MET3	**-5.27**	**1.95***	↑	↓	↓↓	Th/Os/Ox/Gx/ER/Hm/Cw/Cm
IRK4	-5.18	0.93	↓			Os/Ox/Gx
SWE102	-4.80	1.40			↓↓	Th/Os/Ox/Gx/ER/Cm
VRK1	**-4.72**	**1.31***	↑↑			Th/Ox/Gx/ER
PSK201	-4.55	1.08	↑			
YPK101	-4.33	1.17	↑		↓↓	
PAN3	-4.31	1.06	↓↓			Ox/Gx/Cm
CDC2801	-4.27	1.14	↓↓	↓	↓	Os/Ox/ER/Hm
FBP26	**-4.20**	1.20*		↓↓↓		Th/Os/Ox/Gx/ER/Cw/Cm
SSK2	**-4.01**	**1.50***	↑↑↑		↓	Os/Ox/Gx/ER/Hm/Cm
UTR1	**-3.98**	**1.70***	↑↑		↓	Th/Os/Ox/Gx/ER/Hm/Cm
IRK5	-3.61	0.86	↑↑↑	↓↓↓	↑↑↑	Ox/Cm
PHO85	-3.51	0.92	↓			Os/Ox/
PIK1	-3.38	1.27			↓	Th/Os/Ox/Gx/ER/Hm/Cw/Cm
BUD32	**-3.38**	**2.40***	↓↓	↓↓	↓↓	Th/Os/Ox/Gx/ER/Cm
TCO6	-3.29	1.14	↓	↓		Th/Ox/Cm
ARG5,6	**-3.05**	**1.75***	↓		↓↓↓	Th/Os/Ox/Gx/ER/Cw/Cm
SSN3	**-3.04**	**1.67***	↑↑	↓		Os/ER/Cm
IRE1	**-2.80**	**2.30***	↓↓	↓↓		Th/Os/Ox/Gx/ER/Cw/Cm
PKH202	**-2.74**	**1.50***		↓↓↓		Th/Os/Ox/Gx/ER/Cw/Cm
IRK6	-2.74	1.20	↓↓		↑	Os
DAK101	-2.67	1.13		↓	↓↓	Ox
RIM15	-2.55	1.25				Ox
SNF101	-2.53	1.00				Ox
DAK202A	-2.51	0.79	↑	↑		Ox
KIC102	-2.39	2.20*			↑↑↑	Hm/Cw
KIN1	-2.38	1.00	↓↓	↑	↓	
SCH9	**-2.25**	**1.40***	↑↑	↓		Th/Os/Ox/Gx/Cw
MPK2	-2.18	1.17			↑↑↑	Hm/Cm
CEX1	**-1.40**	**1.42***	↓↓			Ox/ER/Hm/Cw/Cm
CKA1	-1.93	1.54*	↓↓	↓↓↓	↓↓	Th/Os/Ox/Gx/ER/Hm/Cw/Cm
BUB1	-1.76	1.40*				Ox
CBK1	-0.18	1.71*		↓↓↓	↓↓	Th/Os/Ox/Gx/ER/Hm/Cw/Cm
MKK2	0.05	2.00*			↑↑	Th/Os/Ox/Gx/ER/Hm/Cw/Cm
YAK103	2.01	1.00				Os/Ox
CMK1	2.51	1.00			↑	Th/Os/Ox/Cm
IRK7	3.63	1.00				Ox

RMS [mt/wt], Relative median survival days = [median survival day for a mutant] / [median survival day for the wild-type strain (H99S); *, $P<0.05$, calculated by Log-rank (Mantel-Cox) test between wild-type and each mutant strain. The RMS and STM scores are average value of each test of two-independent strain. Arrows exhibited enhanced or reduced production of virulence factors including capsule, melanin and urease; single arrow (weak changes), double arrows (moderate changes), triple arrows (strong changes). Abbreviation means as follow; Th, Thermal stress; Os, Osmotic stress; Ox, Oxidative stress; Gx, Genotoxic stress; ER, ER-stress; Hm, Heavy metal stress; Cw, Cell wall stress Cm; Cell membrane stress. Red or blue letters indicate enhanced or reduced sensitivity, respectively, to each stress, while black letter designates the mutants which exhibited enhanced and reduced sensitivity to a stress response depending on stress inducing agents. Bold typed kinases are significant pathogenicity-related kinases which affect the virulence in both insect and murine models of cryptococcosis.

Supplementary Table 2. Kinases involved in antifungal agent resistance in *Cryptococcus neoformans*

Antifungal agents	Kinase mutants showing increased resistance	Kinase mutants showing increased susceptibility
Polyene (Amphotericin B)	Swe102, Tco4, Sps1, Hrk1/Nph1	Hog1, Mkk2, Vps15, Mpk1, Ypk1, Ssk2, Arg5,6, Snf1, Gal83, Pka1, Ipk1, Bud32, Pkh202, Irk3, Cbk1, Irk5, Fbp26, Bck1, Cka1, Yck2, Cdc7, Kin1, Mec1, Pos5, Kic102, Ire1, Utr1, Pan3, Mps1, Gsk3, Pik1, Tco2, Crk1, Hrk1, Pbs2, Alk1, Pkh201, Tpk202a, Ark1, Pka2, Tlk1, Igi1
Azole (Fluconazole)	Ssn3, Kin4, Pka1, Ssk2, Sat4, Bub1, Bud32, Hog1, Sch9, Yak1, Pan3, Yck2, Fbp26, Gal83, Chk1, Adk1, Pbs2, Tco2, Ste11, Pro1, Cpk1, Tco1, Alk1	Mkk2, Vps15, Mpk1, Ipk1, Cbk1, Utr1, Ypk1, Bck1, Ire1, Gsk3, Mpk2, Cdc7, Kic102, Pik1, Kic1, Psk201, Irk6, Cex1, Met3, Igi1, Hrk1/Nph1, Hrk1, Mak3201, Fab1, Rad53, Snf102, Sps1
5-flucyotosine	Urk1, Sat4, Gal83, Vrk1, Irk5, Snf1, Arg5,6, Tco2, Cki1, Ste7, Fab1, Igi1	Vps15, Utr1, Ypk1, Gsk3, Pkh202, Cka1, Mec1, Sch9, Bud32, Ipk1, Ire1, Yck2, Hog1, Ssk2, Cdc7, Kic1, Met3, Pos5, Mps1, Tco6, Cdc2801, Fbp26, Pan3, Bub1, Hrk1/Nph1, Snf102, Pbs2, Adk1, Crk1, Tco1, Chk1

*Red marked gene names indicate the pathogenicity-related kinases which were revealed in this study.

Supplementary Table 3. List of Superfamilies and Families used in the prediction of kinases

SUPERFAMILY	FAMILY
**GHMP Kinase, C-terminal domain**	4-(cytidine 5'-diphospho)-2C-methyl-D-erythritol kinase IspE
**P-loop containing nucleoside triphosphate hydrolases**	**6-phosphofructo-2-kinase/fructose-2,6-bisphosphatase, kinase domain**
**Actin-like ATPase domain**	**Acetokinase-like**
**Protein kinase-like (PK-like)**	Actin-fragmin kinase, catalytic domain
**P-loop containing nucleoside triphosphate hydrolases**	**Adenosine-5'phosphosulfate kinase (APS kinase)**
**ATPase domain of HSP90 chaperone/DNA topoisomerase II/histidine kinase**	**alpha-ketoacid dehydrogenase kinase, C-terminal domain**
**alpha-ketoacid dehydrogenase kinase, N-terminal domain**	alpha-ketoacid dehydrogenase kinase, N-terminal domain
**AMPKBI-like**	**AMPKBI-like**
**Protein kinase-like (PK-like)**	**APH phosphotransferases**
**NTF2-like**	Association domain of calcium/calmodulin-dependent protein kinase type II alpha subunit, CAMK2A
**Riboflavin kinase-like**	**ATP-dependent riboflavin kinase-like**
**Carbamate kinase-like**	Carbamate kinase
**Casein kinase II beta subunit**	**Casein kinase II beta subunit**
**Protein kinase-like (PK-like)**	**Choline kinase**
**Riboflavin kinase-like**	CTP-dependent riboflavin kinase-like
**DAK1/DegV-like**	**DAK1**
**NAD kinase/diacylglycerol kinase-like**	**Diacylglycerol kinase-like**
**FAT domain of focal adhesion kinase**	FAT domain of focal adhesion kinase
**Ribosomal protein S5 domain 2-like**	**GHMP Kinase, N-terminal domain**
**P-loop containing nucleoside triphosphate hydrolases**	**Gluconate kinase**
**Glycerate kinase I**	Glycerate kinase I
**Actin-like ATPase domain**	**Glycerol kinase**
**Glutamine synthetase/guanido kinase**	Guanido kinase catalytic domain
**Guanido kinase N-terminal domain**	Guanido kinase N-terminal domain
**ATPase domain of HSP90 chaperone/DNA topoisomerase II/histidine kinase**	Histidine kinase
**Homodimeric domain of signal transducing histidine kinase**	**Homodimeric domain of signal transducing histidine kinase**
**GHMP Kinase, C-terminal domain**	Homoserine kinase
**HAD-like**	Homoserine kinase ThrH
**PEP carboxykinase-like**	HPr kinase HprK C-terminal domain
**HprK N-terminal domain-like**	HPr kinase/phoshatase HprK N-terminal domain
**SAICAR synthase-like**	**Inositol polyphosphate kinase (IPK)**
**KA1-like**	**Kinase associated domain 1, KA1**
**GHMP Kinase, C-terminal domain**	**Mevalonate kinase**
**Protein kinase-like (PK-like)**	MHCK/EF2 kinase
**Carbamate kinase-like**	**N-acetyl-l-glutamate kinase**
**NAD kinase/diacylglycerol kinase-like**	**NAD kinase-like**
**Nucleoside diphosphate kinase, NDK**	**Nucleoside diphosphate kinase, NDK**
**P-loop containing nucleoside triphosphate hydrolases**	**Nucleotide and nucleoside kinases**
**Ribokinase-like**	**PfkB-like kinase**
**ARM repeat**	**Phoshoinositide 3-kinase (PI3K) helical domain**
**Protein kinase-like (PK-like)**	**Phoshoinositide 3-kinase (PI3K), catalytic domain**

**HAD-like**	**phosphatase domain of polynucleotide kinase**
**SAICAR synthase-like**	**Phosphatidylinositol phosphate kinase IIbeta, PIPK IIbeta**
**Phosphoglycerate kinase**	**Phosphoglycerate kinase**
**AraD/HMP-PK domain-like**	Phosphomethylpyrimidine kinase C-terminal domain-like
**GHMP Kinase, C-terminal domain**	Phosphomevalonate kinase (PMK)
**P-loop containing nucleoside triphosphate hydrolases**	**Phosphoribulokinase/pantothenate kinase**
**Phospholipase D/nuclease**	**Polyphosphate kinase C-terminal domain**
**Cysteine-rich domain**	**Protein kinase cysteine-rich domain (cys2, phorbol-binding domain)**
**Protein kinase-like (PK-like)**	**Protein kinases, catalytic subunit**
**Carbamate kinase-like**	**PyrH-like**
**Phosphoenolpyruvate/pyruvate domain**	Pyruvate kinase
**PK beta-barrel domain-like**	**Pyruvate kinase beta-barrel domain**
**PK C-terminal domain-like**	Pyruvate kinase, C-terminal domain
**Ribokinase-like**	**Ribokinase-like**
**Protein kinase-like (PK-like)**	**RIO1-like kinases**
**Winged helix DNA-binding domain**	**Rio2 serine protein kinase N-terminal domain**
**Histidine-containing phosphotransfer domain, HPT domain**	Sensor-like histidine kinase YojN, C-terminal domain
**Sensory domain-like**	Sensory domain of two-component sensor kinase
**P-loop containing nucleoside triphosphate hydrolases**	**Shikimate kinase (AroK)**
**Ribokinase-like**	**Thiamin biosynthesis kinases**
**P-loop containing nucleoside triphosphate hydrolases**	Type II thymidine kinase
**Glucocorticoid receptor-like (DNA-binding domain)**	Type II thymidine kinase zinc finger
**Ribokinase-like**	**YjeF C-terminal domain-like**

* Superfamilies/Families shown in BOLD indicate the ones found in the putative kinases in *C. neoformans*.

Supplementary Reference

1 Cheon, S. A. *et al.* Unique evolution of the UPR pathway with a novel bZIP transcription factor, Hxl1, for controlling pathogenicity of *Cryptococcus neoformans*. *PLoS Pathog.* **7**, e1002177 (2011).

2 Jung, K. W. *et al.* Systematic functional profiling of transcription factor networks in *Cryptococcus neoformans*. *Nat. Comms.* **6**, 6757 (2015).

3 Hughes, S. *et al.* Crystal structure of human CDC7 kinase in complex with its activator DBF4. *Nat. Struct. Mol. Biol.* **19**, 1101-1107 (2012).

4 Choi, J. *et al.* CFGP 2.0: a versatile web-based platform for supporting comparative and evolutionary genomics of fungi and Oomycetes. *Nucleic Acids Res.* **41**, D714-719 (2013).
